# Darkening of the Global Ocean

**DOI:** 10.1111/gcb.70227

**Published:** 2025-05-27

**Authors:** Thomas W. Davies, Tim Smyth

**Affiliations:** ^1^ School of Biological and Marine Sciences University of Plymouth Plymouth UK; ^2^ Plymouth Marine Laboratory Prospect Place Plymouth UK; ^3^ Centre for Geography and Environmental Science, Department of Earth and Environmental Sciences University of Exeter Cornwall UK

**Keywords:** coastal darkening, moonlight attenuation, ocean darkening, photic zones, photobiology, sunlight attenuation

## Abstract

The photic zones of the oceans—where sunlight and moonlight drive ecological interactions—are one of the most productive habitats on the planet and fundamental to the maintenance of healthy global biogeochemical cycles. Ocean darkening occurs when changes in the optical properties of the oceans reduce the depth to which sufficient light penetrates to facilitate biological processes guided by sunlight and moonlight. We analysed a 9 km resolution annual time series of MODIS Aqua's diffuse attenuation coefficient of light at 490 nm [*K*_d_(490)] to quantify whether the oceans have darkened over the last 20 years and the impact of this on the depth of photic zones around the world. *K*_d_(490) increased across 75,341,181 km^2^ (21%) of the global ocean between 2003 and 2022, resulting in photic zone depths reducing by more than 50 m across 32,449,129 km^2^ (9%) by area. The depth of the photic zone has reduced by more than 10% across 32,446,942 km^2^ (9%) of the global ocean. Our analysis indicates that ocean darkening is not restricted to coastal regions, but affects large swathes of the open ocean. A combination of nutrient, organic material and sediment loading near the coasts and changes in global ocean circulation are probable causes of increases in primary and secondary productivity that have reduced light penetration into surface waters. The implications of ocean darkening for marine ecology and the ecosystem services provided by the surface oceans are currently unknown but likely to be severe.

## Graphical abstract

The ocean is darkening, with less sunlight reaching deeper waters over the past 20 years. Analysing satellite data, we found that 21% of the global ocean has experienced reduced light penetration, with photic zones shrinking by over 50 m in 9% of ocean areas. This darkening isn't limited to coastal waters but extends into the open ocean. Likely causes include increased nutrients, organic matter and shifts in ocean circulation. While the full impact is unknown, this trend could disrupt marine ecosystems, food chains and climate regulation, posing serious risks to ocean health.

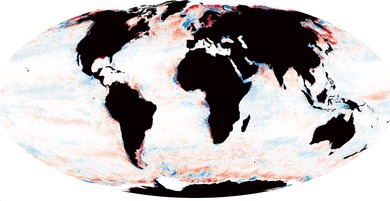

## 1 Introduction

Ninety per cent of all marine life lives in the photic zone of the oceans, where sufficient light penetrates to stimulate photobiological processes. Considered to be around 200 m deep on average, the photic zone is critical for global nutrient and carbon budgets (DeVries 2022; Iversen 2023), sustains global fish stocks (Kourantidou and Jin 2022; Proud et al. 2019) and supports aphotic marine ecosystems where light does not penetrate (Smith Jr et al. 2009; Smith et al. 2020).

Recent years have seen growing concern over the ecological impacts of greening (Cael et al. 2023) and darkening (Blain et al. 2021; Frigstad et al. 2023; Opdal et al. 2019) observed in coastal regions. The attenuation of natural light by elevating concentrations of plankton, suspended particulate matter, coloured dissolved organic matter or other optically active constituents in seawater is reducing the amount of light available for photobiology in the oceans. Conventionally, the photic zone is defined with consideration given only to the influence of sunlight on photosynthesis (Banse 2004; Kirk 1994; Marra et al. 2023; Marra et al. 2014; Ryther 1956). Numerous other photobiological life history adaptations to the moon and sun are, however, near ubiquitous in the epipelagic, such that changes in cycles and gradients of light intensity and spectra can have major ramifications for the structure of marine ecosystems. Beyond primary productivity, the moon and sunlight carry information critical for guiding biological processes essential for the survival and reproduction of marine organisms (Häfker et al. 2023; Hayes 2003; Marshall et al. 2015; Naylor 2001; Thorson 1964). These processes include diel vertical migration (Brierley 2014; Cohen and Forward 2009; Last et al. 2016)—the largest daily migration of biomass on the planet (Hayes 2003), synchronised broadcast spawning (Andreatta and Tessmar‐Raible 2020; Babcock et al. 1986; Häfker et al. 2023; Kaniewska et al. 2015; Lin et al. 2021; Zantke et al. 2013) that is critical for the maintenance of healthy marine invertebrate populations, the partitioning of marine ecosystems in space (vertically with depth) and in time (with daily circannual and circalunar light cycles) (Gerrish et al. 2009; Häfker et al. 2023; Tidau et al. 2022), and a host of visually guided processes (Marshall et al. 2015) including camouflage, predation, communication and orientation. The darkening of the global ocean represents a reduction in the depth to which photobiology driven by the moon and the sun can take place, and as such constitutes a globally widespread form of habitat loss that remains unquantified.

Here we analyse a 20‐year time series of the MODIS Aqua diffuse attenuation coefficient of downwelling irradiance at 490 nm [*K*_d_(490)] to demonstrate that the photic zone has dramatically reduced in depth across large swathes of the global ocean with potentially profound consequences for marine ecosystems.

## 2 Materials and Methods

### 2.1 Quantifying Changes in Light Attenuation

MODIS Aqua's *K*_d_(490) is an algorithm‐derived measure of the attenuation of light at 490 nm in seawater from which the depth of the photic zone can be quantified using Beers law. It is derived from an empirical model of the relationship between the ratio of normalised water‐leaving radiances at wavelengths 490 and 555 nm and in situ measurements (Mueller 2000). We quantified the rate of change in *K*_d_(490) from 2003 to 2022 using 9 km resolution annual average geotiffs downloaded from NASA's Ocean Colour Web data portal level 3 browser on 19/07/2023 (NASA Ocean Biology Processing Group 2022). NASA's MODIS Aqua *K*_d_(490) algorithm performs well in open ocean waters but underestimates *K*_d_(490) in highly turbid coastal waters. Given the global extent of the analysis, alternative algorithms that perform better in coastal waters but less well in open ocean water types were not used to ensure accuracy across the majority of the dataset. Given that we quantify rate changes in *K*_d_(490) and do not present absolute values, our choice of *K*_d_(490) algorithm will have a negligible impact on the results. Annual rather than monthly resolution data were used to remove the influence of seasonal signals in the analysis (Table S1). The 9 km resolution of the data—selected for computational efficiency—means that while our analysis presents a robust indication of the change in *K*_d_(490) at large (regional) spatial scales, caution should be exercised when drawing conclusions about individual pixels, particularly near the coasts where nutrient and sediment loading can be variable at finer spatial resolution.

Data were imported into QGIS v3.24 assuming coordinate reference system (CRS) EPSG: 4326 WGS 84 in units of degrees. A 9 km resolution point grid was created in CRS ESRI: 54004 World Mercator in units of metres and reprojected to EPSG: 4326 WGS 84. Resampling each year of *K*_d_(490) data using the 9 km point grid ensured consistency in the coordinates sampled across all time points. To improve computational efficiency and enhance the resolution of insight provided by the analysis, the global point grid was split into separate International Hydrographic Office regions (https://doi.org/10.14284/323) downloaded on 19/07/2023 (Flanders Marine Institute 2018) enabling batch processing in QGIS v3.24 and later parallel processing in R v4.3.1 following data extraction. *K*_d_(490) was extracted from each point within the grid for each International Hydrographic Office (IHO) region and year. One .csv file was exported for each IHO region containing *K*_d_(490) data across all years for point location.

The rate change in *K*_d_(490) over the 20‐year time series was quantified for each point in the 9 km grid using quantile regression to the median (CRAN: quantreg; R v4.3.1). Regression to the median is insensitive to leverage from outlying data points and requires no a priori assumptions about the underlying data distribution, making it a robust tool for determining rate changes in a property across multiple (9,858,106) locations. Serial autocorrelation between time points was removed by fitting quantile regression models to the best fitted values returned from an Autoregressive Integrated Moving Average (ARIMA) model (CRAN: forecast) (Table S3). For each location, the most parsimonious ARIMA model was selected using Akaike's Information Criterion, assuming *n* = 1 non‐seasonal differences. 95% confidence intervals for the estimates were computed using the rank inverse method and were used to determine whether *K*_d_(490) was significantly changing (not overlapping zero) or not changing (overlapping zero) over time.

### 2.2 Defining the Depth of the Photic Zone

The increasing attenuation of light in the surface oceans inevitably results in a darkening of the photic zone and reductions in the depth to which sufficient light penetrates to enable photobiological processes guided by the sun and the moon. We used Beer's law to calculate the change in photic zone depth between the years 2003 and 2022 based on the predicted values of *K*_d_(490) within each grid point. Conventionally, the depth of the photic zone is considered that at which the in‐water irradiance declines to 1% of surface irradiance (Kirk 1994; Ryther 1956). This definition, however, ignores two critical factors. Firstly, surface irradiance is spatially and temporally variable; hence, 1% of surface irradiance does not translate into the same absolute irradiance value in time and space (Banse 2004). Secondly, all organisms exhibit contrasting sensitivities to light and consequently occupy different photic niches (Häfker et al. 2022). No one value of irradiance can be used to describe the photic niche of all species. Photosensitivity minima at ~490 nm have also not been quantified for a sufficient number of marine species to derive representative averages (Tidau et al. 2021), and commonly recommended alternative measures such as the compensation depth of primary production are exclusive to autotrophic species (Table S1).

To overcome this, we defined the photic zone using the photosensitivity minima of Calanus copepods, a pelagic genus occupying the North Atlantic Ocean that is evolutionarily adapted to low light levels. Calanus spp. undergo daily vertical migrations in response to sunlight and moonlight (Last et al. 2016). During dark periods, they migrate to surface waters to graze on phytoplankton, while during light periods, they migrate to deeper waters to avoid predation, occupying a consistent photic niche as they do (Båtnes et al. 2013). The depth of the photic zone was quantified as the depth where irradiance at 490 nm is equal to the minimum irradiance of 490 nm light that elicits diel vertical migration in Calanus spp. (0.027 μW m^−2^) (Båtnes et al. 2013). The green and blue light sensitivities of Calanus reported by Båtnes et al. (2013) are particularly useful when calculating photic zone depths using remote or in situ collected *K*_d_(490) data (Davies et al. 2020) as the median value of the λ_max_ reported for the blue (455 nm) and green (525 nm) light sources used is 490 nm. The extreme sensitivity of Calanus to light means that any absolute photic zone depths quantified will be large compared to many species; however, the proportional reduction in photic zone depth between 2003 and 2022 will be the same for all species regardless of their photosensitivity. Using a highly sensitive species like Calanus also aligns our results with the precautionary principle, since for the majority of taxa, absolute reductions in photic zone depth are unlikely to be as large as reported here.

### 2.3 Quantifying Reductions in Photic Zone Depth

We used Beer's law to calculate the change in photic zone depth between the years 2003 and 2022 based on the predicted values of *K*_d_(490) within each grid point, and assuming that *K*_d_(490) is invariant with depth. Photic zone depths (*Z*_photic_) in metres were calculated separately for the maximum moonlight (full moon at zenith) and sunlight (sun at zenith) irradiances experienced at the sea surface in each month of a typical year and then averaged. Where the depth of the photic zone exceeded the bathymetric depth, the bathymetric depth was taken as the maximum depth of the photic zone.

For comparison, we also calculated photic zone depths using the traditional definition of 1% of surface irradiance (Ryther 1956). Using this definition, photic zone depths are unaffected by absolute irradiances at the sea surface and so are identical for moonlight and sunlight and do not account for latitudinal variability in surface irradiance.

Photic zone depths (*Z*_photic_) in metres were calculated separately for the maximum moonlight (full moon at zenith) and sunlight (sun at zenith) irradiances experienced at the sea surface in each month of a typical year as follows:

(1) $$Z_{\text{photic}}=-\frac{1}{K_{d}\left(490\right)}\ln\left[\frac{I_{\text{sens}}\left(490\right)}{I_{\text{osolar}}\left(490\right)}\right]\left(m\right)$$

and

(2) $$Z_{\text{photic}}=-\frac{1}{K_{d}\left(490\right)}\ln\left[\frac{I_{\text{sens}}\left(490\right)}{I_{\text{olunar}}\left(490\right)}\right]\;\left(m\right)$$

where *K*_d_(490) is the predicted value of *K*_d_(490) for either the year 2003 or 2022 at each grid point generalised to be invariant with depth (see Table S1 for justification); *I*_sens_(490) is the minimum irradiance of 490 nm light that elicits diel vertical migration in Calanus copepods, *I*_osolar_(490) is the irradiance (W m^−2^) of 490 nm sunlight at the sea surface, and *I*_olunar_ is the irradiance (W m^−2^) of 490 nm moonlight at the sea surface. Our approach does not account for complexity in the water column [i.e., mixed layer depth, chlorophyll deep water maximum and values of deep water *K*_d_(490)] due to a lack of available information.

Once calculated, the *Z*_photic_ values were corrected for the bathymetric depth (m) at each grid point using the GEBCO 2023 sub‐ice bathymetry (https://doi.org/10.5285/f98b053b‐0cbc‐6c23‐e053‐6c86abc0af7b) downloaded on 01/11/2023 (GEBCO Bathymetric Compilation Group 2023). Where *Z*_photic_ exceeded bathymetric depth, the bathymetric depth was used as the value of *Z*_photic_ for calculating Δ*Z*_photic_ below. The change in photic depth between 2003 and 2022 was then calculated separately for moonlight and sunlight at each grid point and month of the year as:

(3) $$∆Z_{\text{photic}}=Z_{\text{photic}}\left(2022\right)-Z_{\text{photic}}\left(2003\right)\left(m\right)$$

where *K*_d_(490) was significantly changing over time, the slope and intercept coefficients were used to predict its modelled value in 2003 and 2022 for the purpose of calculating *Z*_photic_. Where *K*_d_(490) was not significantly changing over time, the median value of *K*_d_(490) across years was assumed as the 2003 and 2022 values (i.e., no change in *K*_d_(490)).

### 2.4 Solar and Lunar Irradiance Models

The top of atmosphere (TOA) spectral solar irradiances, *I*_0_(λ), at 1‐nm resolution, were calculated using a look‐up table (Neckel 1984) of the solar spectral irradiance, *H*_0_(λ) and corrected for the eccentricity (ε) of Earth's orbit (function of day of year, D) using the equation:

(4) $$I_{0}\left(\lambda \right)=H_{0}\left(\lambda \right){\left(1+\varepsilon \cos\left\{\frac{2\pi \left(D-3\right)}{365}\right\}\right)}^{2}\left({\mathrm{Wm}}^{-2}\right)$$

The Gregg and Carder (1990) spectral marine atmosphere model was used to determine the spectral (just) above‐surface solar irradiance, *I*_d_(λ, 0+), assuming clear sky conditions and using the settings shown in Table S2. This model is relatively simple but does consider gaseous absorption and aerosol optical properties and allows for the partitioning of the irradiance field into direct, *I*_dd_(λ), and diffuse, *I*_ds_(λ), components. The global above‐surface spectral irradiance is the sum of these two terms:

(5) $$I_{d}\left(\lambda ,0^{+}\right)=I_{\mathrm{dd}}\left(\lambda \right)+I_{\mathrm{ds}}\left(\lambda \right)\left({\mathrm{Wm}}^{-2}\right)$$

and was integrated over the wavelength range 485–495 nm, at 9 km (0.08° latitude) resolution for the 15th day of each calendar month (of 2022) at solar noon (Table S1).

### 2.5 Lunar Spectral Model

The TOA spectral lunar irradiances were determined using the TOA spectral solar irradiances (Equation 4), spectrally varying lunar albedo (Table S2) of (Velikodsky et al. 2011) and a lunar semi‐diameter view angle of 0.26°. The lunar zenith angle was calculated as a function of location, date and time using the Python astropy (https://pypi.org/project/astropy) package. The phase curve of Lumme and Bowell (1981) was used to account for the full moon brightening, and the lunar phase was calculated as a function of latitude, date and time using the Python astroplan (https://pypi.org/project/astroplan) package. The Gregg and Carder (1990) model was then used to determine the spectral surface lunar irradiance, assuming clear sky conditions (Table S2) and was integrated over the wavelength range 485–495 nm, at 9 km (0.08° latitude) resolution for the lunar culmination date of each calendar month of 2022 (Table S3). For each 9 km grid point, the time when the moon reached its highest altitude was used to calculate the full moon irradiance for that month (Table S1).

## 3 Results

### 3.1 Changes in Light Attenuation

**FIGURE 1:**
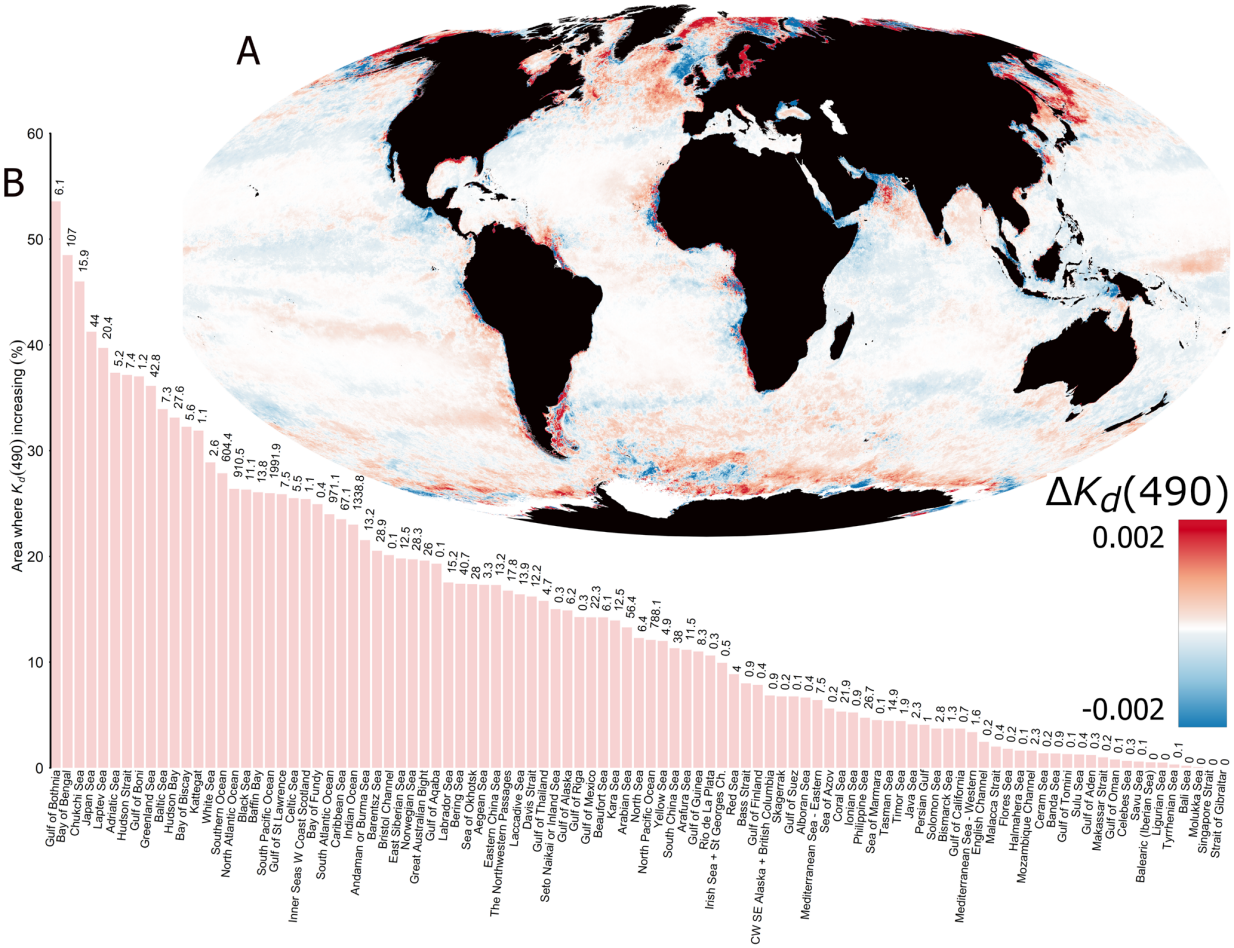
Darkening of the Global Ocean. (A) The rate change (units per year) in the diffuse attenuation coefficient for downwelling irradiance at 490 nm [Δ*K*_d_(490)] measured from MODIS Aqua between 2003 and 2022 in 9 km resolution (CRS: World Mollweide, ESRI:54009). Reds indicate regions where *K*_d_(490) is increasing (oceans are getting darker), while blues indicate regions where *K*_d_(490) is decreasing (oceans are getting lighter). White indicates regions where there was no statistically significant change in *K*_d_(490) over the period 2003–2022. Values of Δ*K*_d_(490) were derived for each 9 km resolution pixel using quantile regression to the median performed on the fitted values of an Autoregressive Integrated Moving Average (ARIMA) model of annual averaged *K*_d_(490) data. (B) International Hydrographic Office sea regions ranked according to the percentage of their area over which *K*_d_(490) is increasing. Numbers inset above bars give the absolute area where *K*_d_(490) is increasing in 10,000 km^2^. Raw data are presented in Table S4. Areas were calculated in CRS World Mercator (ESRI:54004).

*K*_d_(490) increased across 75,341,181 km^2^ (21%) and decreased across 37,269,515 km^2^ (10%) of the global ocean between 2003 and 2022 (Figure 1A), indicating that the oceans have become darker over the last two decades. Increases in *K*_d_(490) occurred over more than 50% of the area of one International Hydrographic Office (IHO) region (The Gulf of Bothnia, although the *K*_d_(490) algorithm underestimates in this region), 40% of the area of four IHO regions, 30% of the area of 13 IHO regions, 20% of the area of 29 IHO regions and 10% of the area of 56 IHO regions (Figure 1B, Table S4). Increasing attenuation of light is not restricted to coastal zones but is also widespread in the major oceans, particularly in polar regions, the North East Atlantic and the North West Pacific Oceans (Figure 1A).

### 3.2 Changes in Photic Zone Depth

**FIGURE 2:**
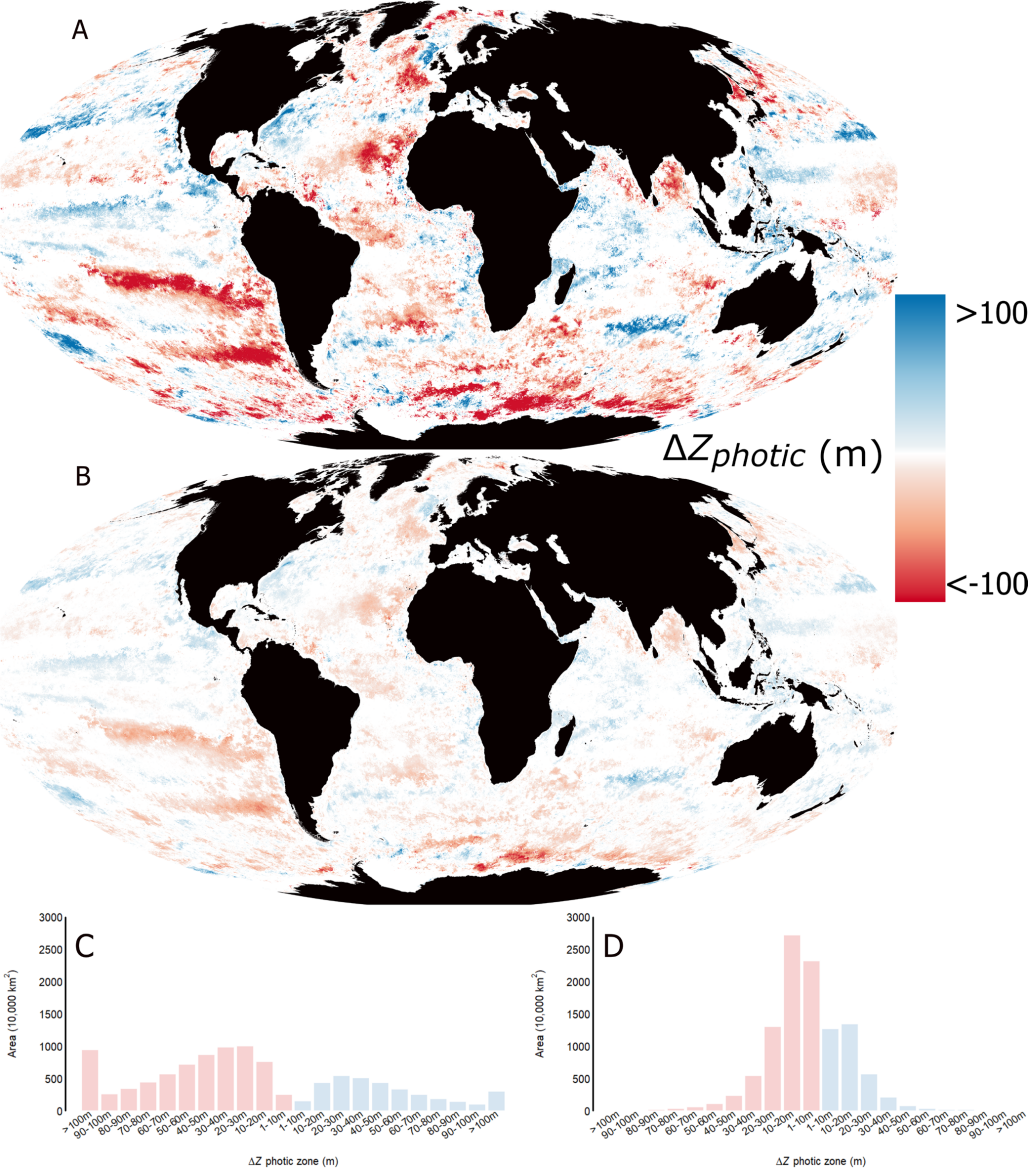
Loss of the global photic zone. Changes in photic zone depth (Δ*Z*_photic_) in metres between 2003 and 2022 under sunlight (A,C) and full moon (B,D) irradiances. Changes in Δ*Z*_photic_ expressed as a percentage difference to 2003 are given in Figure S1. The photic zone was defined as the depth at which the minimum irradiance of 490 nm light occurs that elicits diel vertical migration in Calanus copepods. Surface irradiances at zenith were modelled at 9 km resolution for the median day in each month of a typical year and used to quantify Δ*Z*_photic_ with the annual average presented. The area of the global ocean (in 10,000 km^2^) across which *Z*_photic_ is reduced by 10 m increment depths is presented in C and D. Red corresponds to reductions in *Z*_photic_ while blue indicates increases. The dashed line gives the total area of deforested land cover since year 2000 as a point of reference. A breakdown of C and D by IHO region can be found in Tables S5 and S7.

When considering photobiological processes guided by sunlight, the photic zone has reduced by more than 100 m across 9,392,219 km^2^ (2.6%) of the global ocean (Figure 2A,C, Table S5) between 2003 and 2022. The area reduced by more than 50 m depth was 32,449,129 km^2^ (9%) and the area reduced by more than 10 m depth was 68,402,842 km^2^ (19%). Between 2003 and 2022, the average depth of the photic zone during daylight was reduced by more than 50% across 369,248 km^2^ of the ocean by area and more than 10% across 32,446,942 km^2^ (9%) (Figure S1A,C, Table S5).

Due to the lower sea surface irradiances of moonlight, reductions in photic zone depth during the nighttime are much smaller (Figure 2B,D, Table S7). In this study, 45,418 km^2^ of ocean have seen the depth of the nighttime photic zone reduce by more than 100 m, 2,093,566 km^2^ (0.6%) by more than 50 m and 49,829,779 km^2^ (13.9%) by more than 10 m (Figure 2A,B, Table S7). Between 2003 and 2022, the average depth of the photic zone illuminated by moonlight reduced by more than 50% across 273,590 km^2^ of the ocean and more than 10% across 34,074,665 km^2^ (9.5%) (Figure S1B,D, Table S8).

When calculated using the 1% threshold definition of photic zone depth, the impact of coastal darkening on photic zone depths is inevitably smaller compared to when using the Calanus definition, but nonetheless equally important (Table S9). Photic zones have reduced by more than 100 m across 40,489 km^2^ (0.01%) of the global ocean, by more than 50 m across 1,415,546 km^2^ (0.39%) and by more than 10 m across 39,970,126 km^2^ (11.12%). These quantities are, however, likely to be gross underestimates of the true impact of ocean darkening on photic zones because the 1% of surface irradiance definition does not account for the high photosensitivity of many marine organisms and the impact of variability in surface irradiance in space and time.

## 4 Discussion

The surface oceans have changed colour over the last 20 years, indicating global scale shifts in ocean ecology (Cael et al. 2023; Dutkiewicz et al. 2019) caused potentially by blooms in phytoplankton and zooplankton. Our results provide evidence that the global ocean has also become darker over the last 20 years, and that this has resulted in a net loss of photic zones at a large spatial scales. Darkening of the ocean has recently been implicated as a driver of changes in the timing of phytoplankton blooms and reproductive phenology in cod (Opdal et al. 2024). Changing underwater light regimes caused by darkening could result in the mismatch of phenological events and the erosion of both the diel and vertical light niches that are critical for partitioning ocean biodiversity in time and space (Häfker et al. 2022). A key example is the vertical migration of zooplankton—the largest daily mass migration of biomass on the planet (Hayes 2003)—that will likely be compressed to shallower depths in darker waters.

Darkening has typically been associated with coastal regions. Our results indicate that increasing attenuation of light is not restricted to the coastal zones but also widespread in the major oceans, particularly in polar regions, the North East Atlantic and North West Pacific Oceans. This suggests that light attenuation across the oceans is not driven exclusively by localised nutrient loading, run‐off and upwelling in coastal regions. While these factors will clearly influence light attenuation in neritic coastal waters (Aksnes et al. 2009; Blain et al. 2021; Frigstad et al. 2023; Opdal et al. 2024), darkening in the open oceans may be driven by warming of the surface oceans and climate‐driven changes in the ocean circulation patterns (Böning et al. 2008; Cael et al. 2023; Martínez‐Moreno et al. 2021; Toggweiler and Russell 2008). Changes in ocean surface chlorophyll concentrations have been documented since the turn of the 21st century and linked to changes in sea surface temperature (Polovina et al. 2008). These regionally specific drivers and the complexity with which they interact make a comprehensive quantification of the drivers of ocean darkening a significant undertaking and one that lies beyond the scope of this study. Nonetheless, we envisage that regional analyses of darkening drivers represent a logical next step for research into this emerging global change issue.

When exploring the potential causes of ocean darkening, the research community should remain aware that while MODIS Aqua represents the longest running time series of ocean colour data collected by any single satellite sensor, 20 years is nonetheless insufficient to completely rule out the possibility that these trends reflect natural multidecadal variability (Beaulieu et al. 2013). Global changes in ocean colour detected by MODIS Aqua have recently been attributed to non‐natural causes in other studies; however (Cael et al. 2023), and the scale of the losses in photic zone depth over the last 20 years is so profound that we suggest they represent one of the largest losses of habitat on the planet and warrant further investigation. When calculating *ΔZ*_photic_ from *ΔK*_d_(490) we also assumed that the water column was fully mixed (*K*_d_(490) is invariant with depth), while in reality atmospheric conditions lead to the stratification and mixing (vertical complexity). Our results should therefore be seen as indicative of the general magnitude and direction of trend, and not predictive of any given point in time.

Changes in photic zone depth at nighttime were small compared to the daytime, but remained ecologically important. Biological adaptations to moonlight are common in marine organisms including synchronised reproductive events (Babcock et al. 1986; Kaniewska et al. 2015; Lin et al. 2021; Naylor 2001) and migrations (Hayes 2003; Ugolini et al. 2012). These organisms are adapted to detect very low light intensities and even small changes in the depth of the photic zone could have important ramifications for survival and reproduction, particularly in sessile species (e.g., corals) that are unable to adjust their position in the water column.

While the attenuation of light has clearly increased in many coastal regions (Figure 1A), this does not often translate into a net reduction in photic zone depth near shore (Figure 2A,B) as defined here, due to shallow bathymetry and the relatively high photosensitivity of Calanus spp. For many less sensitive and particularly diurnal marine species, increases in light attenuation will likely translate into losses in their fundamental photic niche space where bathymetry is shallow near the coasts. Unfortunately, photosensitivity minima close to 490 nm have not been quantified for less photosensitive species (Tidau et al. 2021). In the absence of such fundamental knowledge of species photobiology, it will be challenging to understand the scale of impact that ocean darkening is having on marine ecosystems.

Without sufficient light with which to grow, move, hunt, communicate, reproduce and photosynthesise, marine organisms will be forced to migrate vertically into an increasingly smaller belt of sufficiently lit surface waters, exposing them to higher levels of competition for resources and elevated risk of predation. The implications for marine food webs, global fisheries, carbon and nutrient budgets could be severe.

## Author Contributions

**Thomas W. Davies:** conceptualization, formal analysis, funding acquisition, investigation, methodology, project administration, visualization, writing – original draft, writing – review and editing. **Tim Smyth:** conceptualization, formal analysis, funding acquisition, methodology, writing – review and editing.

## Conflicts of Interest

The authors declare no conflicts of interest.

## Supporting information


Data S1.

## Data Availability

The data and code that support the findings of this study are openly available in Zenodo at https://doi.org/10.5281/zenodo.15224272. Kd(490) data were obtained from the NASA Ocean Biology Distributed Active Archive Center at https://doi.org/10.5067/AQUA/MODIS/L3M/KD/2022. The International Hydrographic Office regions data were obtained from the Flanders Marine Institute at https://doi.org/10.14284/323. GEBCO 2023 sub ice bathymetry data were obtained from the NERC EDS British Oceanographic Data Centre NOC at https://doi.org/10.5285/f98b053b‐0cbc‐6c23‐e053‐6c86abc0af7.
