# Supplementary material for: Darkening of the Global Ocean
Source: Glob Chang Biol. 2025 May 27;31(5):e70227. doi: 10.1111/gcb.70227 (PMC12107402; doi:10.1111/gcb.70227)
Supplement: Supplementary file 1 — Data S1. [file GCB-31-e70227-s001.docx]

Supplementary Information for Davies *et al*. (2024) “*Darkening of the Global Ocean*”

**Table S1| Known limitations of the approach used to determine Z_photic_.** Generalisations and assumptions made in the modelling.

| Parameter | Modelled generalisation | Rationale for using generalisation | | Alternative Options | Cost / **Benefit** of Alternative Options | |
| --- | --- | --- | --- | --- | --- | --- |
| K_d_(490) (t) | (Modelled, from quantile regression to the median) annual average K_d_(490) for each 9km pixel for years 2003 and 2022.  Matrix dimensions: 4096 x 2048 x 2 | | 1) Allows direct calculation of ΔK_d_(490) from an empirically derived relationship between K_d_(490) and time.  2) De-seasonalises the K_d_(490) time-series.  3) Maximises the number of clear pixels available from the dataset cf. monthly datasets. | Use monthly global K_d_(490) composite data for each month between 2003 – 2022. | **Introduce the influence of seasonality and intra-annual variability on the calculations.**  Would provide little additional insight of ΔK_d_(490) over the time-series.  Significant increase in computations (approximately 2 orders of magnitude). Matrix dimensions: 4096 x 2048 x 20 x 12 |  |
| K_d_(490) (z) | Depth invariant K_d_(490) for each 9km pixel for years 2003 and 2022 (see row above).  Matrix dimensions: 4096 x 2048 x 2 | | Simplicity of approach requiring no further models, assumptions or generalisations. | Include mixed layer depth (MLD) in calculations: this could use a two layer model with satellite derived K_d_(490) for the top optical depth within MLD. Will need to assume a value of K_d_(490) below MLD.  Use modelled chlorophyll profile, and hence K_d_(490), based on surface values of chlorophyll^[[1]](#footnote-1)^ | **Possible increase in the realism of the variability of K_d_(490) with depth.** Little scientifically available evidence to back this up at each grid point – i.e., generalisation replaced by assumption.  Matrix dimensions: 4096 x 2048 x 2 x 2 (for using modelled generalisation in row 1) Matrix dimensions: 4096 x 2048 x 20 x 12 x 2 (for using monthly data)  Modelled chlorophyll profile. Matrix dimensions: 4096 x 2048 x 20 x 12 x N depths.  Significant increase in computations. |  |
| I_osolar_ (490) | Mid-monthly value for a given latitude, applied to the annual value of K_d_(490) for 2003 and 2022, and annual average calculated.  Matrix dimensions: 4096 x 2048 x 12 x 2 | | Simplicity of approach, significantly reducing the number of calculations. Retains the all-important spectral information rather than just being broadband irradiance. | Hourly (or daily) calculations for period between 2003 – 2022. | **Captures diurnal variability in solar angle and impact on surface irradiance.**  Significant increase in computations. Matrix dimensions: 4096 x 2048 x 20 x 365 (x 24) |  |
| I_olunar_ (490) | Full moon at zenith for each month (in 2022), applied to the annual value of K_d_(490) for 2003 and 2022, and annual average calculated.  Matrix dimensions: 4096 x 2048 x 12 x 2 | | Simplicity of approach, significantly reducing the number of calculations. Retains the all-important spectral information rather than just being broadband irradiance. | Hourly (or daily) calculations which include the lunar phases, moon rise, set times relative to the solar. Would likely need to have multiple years. | **Captures nocturnal variability in lunar angle and impact on surface irradiance.**  Significant increase in computations. Matrix dimensions: 4096 x 2048 x 20 x 365 (x 24). |  |
| Cloud Cover | Clear Skies  Matrix dimensions: 4096 x 2048 x 12 x 2 | | Retains sufficient complexity to be representative of the real world (standard type atmospheric conditions for multi-spectral irradiance) without becoming computationally intractable. | Include a cloud climatology within the calculations, either at annual or monthly time resolution. | **Allows extra realism to be introduced to the model.**  Impact of clouds on surface irradiance too complex to model if only cloud cover available. Significant increase in computations for little demonstrable gain.  Matrix dimensions: 4096 x 2048 x 12 x 20 x 2 |  |
| Z_photic_ | Calanus definition | | Inclusive of all species sensitivities to celestial light in the photic zone.  Insufficient evidence to reliably calculate representative measures without introducing taxonomic bias. | Alternative definitions of photic zone depth:-   1. 1% of surface irradiance 2. Compensation depth of phytoplankton. 3. Depth of photosynthesis. | 1.Calculate and provided for comparison. The 1% threshold is not biologically relevant and is a measure of light attenuation rather than photic zone depth.  2&3. These measures are exclusive to autotrophs and do not account for heterotrophs, many of which are far more sensitive to light. |  |

**Table S2| Parameters used by the Gregg and Carder (1990) model.** Marine aerosol model utilized.

| **Parameter** | **Symbol** | **Value** |
| --- | --- | --- |
| Atmospheric pressure | p0 | 1013.25 mb |
| Ozone concentration | O_3_ | 300 Dobson Units |
| Precipitable water vapour |  | 1.5 cm |
| Relative humidity | RH | 80% |
| Wind speed |  | 5.0 ms^-1^ |
| Visibility |  | 15.0 km |

**Table S3 |** **Lunar culmination dates in 2022.** Data validated against mooncalc.org for times in UTC. The year 2022 was chosen as it contains a single full moon within each calendar month.

| **Month** | **Day** | **Time** | **Time Zone** |
| --- | --- | --- | --- |
| January | 18 | 00:15 | UTC |
| February | 15 | 23:48 | UTC |
| March | 18 | 00:01 | UTC |
| April | 17 | 00:13 | UTC |
| May | 15 | 23:46 | UTC |
| June | 13 | 23:27 | UTC |
| July | 14 | 00:24 | UTC |
| August | 12 | 00:08 | UTC |
| September | 09 | 23:43 | UTC |
| October | 08 | 23:10 | UTC |
| November | 07 | 23:20 | UTC |
| December | 07 | 23:41 | UTC |

**Table S4| Areas of the global ocean and International Hydrographic Office (IHO) regions where the diffuse attenuation coefficient for downwelling irradiance at 490nm [*k_d_*(490)] is increasing, decreasing and not changing between 2003 and 2022.** Areas were calculated from IHO region areas (km^2^) using the number of 9km resolution pixels that were classified into each category. Pixels were classified using the sign of the slope estimate from a quantile regression (on the median) model of annual average *k_d_*(490) against year where the slope estimate was significantly different from zero. Pixels were classified as not changing where the slope estimate was not significantly different from zero. Quantile regressions were modelled on the fitted values returned from an Autoregressive Integrated Moving Average (ARIMA) model with n=1 non-seasonal differences to remove serial autocorrelation from the data.

| IHO region | Area *k_d_*(490) increasing | | Area *k_d_*(490) decreasing | | Area *k_d_*(490) not changing | |
| --- | --- | --- | --- | --- | --- | --- |
|  | (km^2^) | (%) | (km^2^) | (%) | (km^2^) | (%) |
| **Global Ocean** | **75341181** | **21** | **37269515** | **10** | **246741085** | **69** |
| Adriatic Sea | 52092 | 37 | 1673 | 1 | 85689 | 61 |
| Aegean Sea | 33101 | 17 | 36852 | 19 | 121352 | 63 |
| Alboran Sea | 3633 | 7 | 12609 | 23 | 38469 | 70 |
| Andaman or Burma Sea | 132037 | 22 | 23703 | 4 | 457358 | 75 |
| Arabian Sea | 563567 | 13 | 505079 | 12 | 3172538 | 75 |
| Arafura Sea | 114598 | 11 | 275719 | 27 | 635419 | 62 |
| Baffin Bay | 138074 | 26 | 17735 | 3 | 374151 | 71 |
| Balearic (Iberian Sea) | 427 | 1 | 21896 | 27 | 57755 | 72 |
| Bali Sea | 81 | 0 | 12631 | 32 | 27204 | 68 |
| Baltic Sea | 73203 | 34 | 2308 | 1 | 140352 | 65 |
| Banda Sea | 9422 | 1 | 258546 | 37 | 425874 | 61 |
| Barentsz Sea | 288936 | 21 | 115555 | 8 | 1003938 | 71 |
| Bass Strait | 9000 | 8 | 24995 | 22 | 78703 | 70 |
| Bay of Bengal | 1070403 | 48 | 53532 | 2 | 1083630 | 49 |
| Bay of Biscay | 56260 | 32 | 7194 | 4 | 110983 | 64 |
| Bay of Fundy | 3998 | 25 | 2086 | 13 | 9951 | 62 |
| Beaufort Sea | 61312 | 14 | 50524 | 12 | 319296 | 74 |
| Bering Sea | 406855 | 17 | 312814 | 13 | 1617243 | 69 |
| Bismarck Sea | 12825 | 4 | 97291 | 28 | 234937 | 68 |
| Black Sea | 111293 | 26 | 97495 | 23 | 214238 | 51 |
| Bristol Channel | 1157 | 20 | 105 | 2 | 4486 | 78 |
| Caribbean Sea | 670856 | 24 | 285607 | 10 | 1896330 | 66 |
| Celebes Sea | 2957 | 1 | 157951 | 35 | 296434 | 65 |
| Celtic Sea | 54859 | 26 | 12762 | 6 | 147459 | 69 |
| Ceram Sea | 2227 | 1 | 73322 | 45 | 85941 | 53 |
| Chukchi Sea | 159233 | 46 | 9386 | 3 | 177641 | 51 |
| Coral Sea | 219134 | 5 | 721154 | 17 | 3185254 | 77 |
| Davis Strait | 121537 | 16 | 23218 | 3 | 604982 | 81 |
| East Siberian Sea | 125367 | 20 | 77490 | 12 | 430201 | 68 |
| Eastern China Sea | 131624 | 17 | 90868 | 12 | 538864 | 71 |
| English Channel | 1997 | 2 | 16555 | 20 | 62867 | 77 |
| Flores Sea | 1858 | 2 | 8481 | 8 | 92478 | 90 |
| Great Australian Bight | 259873 | 20 | 54889 | 4 | 1011447 | 76 |
| Greenland Sea | 428466 | 36 | 50446 | 4 | 707544 | 60 |
| Gulf of Aden | 3189 | 1 | 91392 | 35 | 169173 | 64 |
| Gulf of Alaska | 61840 | 15 | 37265 | 9 | 316398 | 76 |
| Gulf of Aqaba | 686 | 19 | 0 | 0 | 2869 | 81 |
| Gulf of Boni | 12301 | 37 | 326 | 1 | 20610 | 62 |
| Gulf of Bothnia | 60762 | 54 | 816 | 1 | 51841 | 46 |
| Gulf of California | 6695 | 4 | 36206 | 20 | 137802 | 76 |
| Gulf of Finland | 4480 | 8 | 9824 | 17 | 42773 | 75 |
| Gulf of Guinea | 83038 | 11 | 127649 | 17 | 544157 | 72 |
| Gulf of Mexico | 222882 | 14 | 254521 | 16 | 1089356 | 70 |
| Gulf of Oman | 883 | 1 | 38701 | 35 | 72309 | 65 |
| Gulf of Riga | 2664 | 14 | 505 | 3 | 15523 | 83 |
| Gulf of St Lawrence | 75277 | 26 | 19455 | 7 | 196142 | 67 |
| Gulf of Suez | 704 | 7 | 2241 | 21 | 7492 | 72 |
| Gulf of Thailand | 47041 | 16 | 27121 | 9 | 223765 | 75 |
| Gulf of Tomini | 740 | 1 | 18173 | 32 | 37826 | 67 |
| Halmahera Sea | 1224 | 2 | 54575 | 72 | 19497 | 26 |
| Hudson Bay | 275871 | 33 | 12995 | 2 | 543784 | 65 |
| Hudson Strait | 74312 | 37 | 3165 | 2 | 122562 | 61 |
| Indian Ocean | 13387687 | 23 | 4386694 | 8 | 40456573 | 69 |
| Inner Seas off the West Coast of Scotland | 11252 | 25 | 5471 | 12 | 27537 | 62 |
| Ionian Sea | 8998 | 5 | 12436 | 7 | 150344 | 88 |
| Irish Sea and St Georges Channel | 4566 | 10 | 7329 | 16 | 34027 | 74 |
| Japan Sea | 439627 | 41 | 49373 | 5 | 577306 | 54 |
| Java Sea | 23272 | 4 | 221533 | 39 | 321888 | 57 |
| Kara Sea | 125027 | 14 | 125806 | 14 | 646105 | 72 |
| Kattegat | 11305 | 32 | 77 | 0 | 24065 | 68 |
| Labrador Sea | 151931 | 18 | 30722 | 4 | 683817 | 79 |
| Laccadive Sea | 138854 | 16 | 66041 | 8 | 641456 | 76 |
| Laptev Sea | 204012 | 40 | 14263 | 3 | 295392 | 58 |
| Ligurian Sea | 85 | 1 | 9068 | 54 | 7797 | 46 |
| Makassar Strait | 1976 | 1 | 88025 | 44 | 108116 | 55 |
| Malacca Strait | 3940 | 2 | 63783 | 33 | 127811 | 65 |
| Mediterranean Sea - Eastern Basin | 75194 | 6 | 66528 | 6 | 1031918 | 88 |
| Mediterranean Sea - Western Basin | 16136 | 3 | 42334 | 9 | 418237 | 88 |
| Molukka Sea | 164 | 0 | 82356 | 37 | 139151 | 63 |
| Mozambique Channel | 22624 | 2 | 420721 | 30 | 950938 | 68 |
| North Atlantic Ocean | 9104795 | 26 | 3983993 | 12 | 21418340 | 62 |
| North Pacific Ocean | 7880862 | 12 | 8999728 | 14 | 48274091 | 74 |
| North Sea | 64392 | 12 | 70291 | 13 | 389810 | 74 |
| Norwegian Sea | 283296 | 20 | 94771 | 7 | 1059028 | 74 |
| Persian Gulf | 9942 | 4 | 117456 | 48 | 117259 | 48 |
| Philippine Sea | 267317 | 5 | 1587965 | 28 | 3786714 | 67 |
| Red Sea | 39856 | 9 | 101862 | 23 | 308094 | 68 |
| Rio de La Plata | 3377 | 11 | 828 | 3 | 27591 | 87 |
| Savu Sea | 637 | 1 | 9158 | 9 | 96439 | 91 |
| Sea of Azov | 2217 | 6 | 32976 | 83 | 4355 | 11 |
| Sea of Marmara | 526 | 5 | 5024 | 43 | 6125 | 52 |
| Sea of Okhotsk | 280104 | 17 | 166751 | 10 | 1166354 | 72 |
| Seto Naikai or Inland Sea | 2720 | 15 | 3513 | 19 | 11898 | 66 |
| Singapore Strait | 0 | 0 | 1111 | 41 | 1573 | 59 |
| Skagerrak | 2164 | 7 | 3281 | 10 | 26595 | 83 |
| Solomon Sea | 27672 | 4 | 207660 | 28 | 509003 | 68 |
| South Atlantic Ocean | 9711020 | 24 | 3489023 | 9 | 27301769 | 67 |
| South China Sea | 379998 | 11 | 684199 | 20 | 2298707 | 68 |
| South Pacific Ocean | 19918606 | 26 | 4873653 | 6 | 51888914 | 68 |
| Southern Ocean | 6043586 | 28 | 1647594 | 8 | 14003977 | 65 |
| Strait of Gibraltar | 0 | 0 | 555 | 33 | 1109 | 67 |
| Sulu Sea | 4260 | 1 | 65509 | 19 | 267260 | 79 |
| Tasman Sea | 148827 | 4 | 671509 | 20 | 2524288 | 75 |
| The Coastal Waters of Southeast Alaska and British Columbia | 8514 | 7 | 15358 | 12 | 100660 | 81 |
| The Northwestern Passages | 177956 | 17 | 180327 | 17 | 703889 | 66 |
| Timor Sea | 19209 | 4 | 182088 | 42 | 232889 | 54 |
| Tyrrhenian Sea | 713 | 0 | 51017 | 23 | 165735 | 76 |
| White Sea | 26147 | 29 | 8675 | 10 | 55708 | 62 |
| Yellow Sea | 48965 | 12 | 73686 | 18 | 285547 | 70 |

**Table S5| Absolute changes in the depth of the photic zone (*Z_photic_*) across the global ocean and International Hydrographic Office (IHO) regions between 2003 and 2022 when illuminated by sunlight.** *Z_photic_* was calculated using Beers Law (see methods). The annual average of monthly maximum zenith surface solar irradiance was modelled at 9km resolution. The depth of the photic zone was defined as the minimum irradiance at 520nm that elicits diel vertical migration in Calanus copepods. *K_d_*(490) was taken as the modelled values for 2003 and 2022 given by quantile regression on the median, with serial autocorrelation removed using ARIMA models.

| IHO region | Total Area (km^2^) | Area where *Z_photic_* is decreasing (km^2^) | | | | | | | | | | | | Area no change in *Z_photic_* (km^2^) | Area where *Z_photic_* is increasing (km^2^) | | | | | | | | | | | |
| --- | --- | --- | --- | --- | --- | --- | --- | --- | --- | --- | --- | --- | --- | --- | --- | --- | --- | --- | --- | --- | --- | --- | --- | --- | --- | --- |
|  |  | >100m | 90-100m | 80-90m | 70-80m | 60-70m | 50-60m | 40-50m | 30-40m | 20-30m | 10-20m | 1-10m | 0-1m |  | 0-1m | 1-10m | 10-20m | 20-30m | 30-40m | 40-50m | 50-60m | 60-70m | 70-80m | 80-90m | 90-100m | >100m |
| **Global Ocean** | **359351781** | **9392219** | **2557548** | **3365186** | **4369021** | **5641601** | **7123554** | **8613114** | **9765880** | **9992158** | **7582561** | **2425774** | **200191** | **254937889** | **67468** | **1434307** | **4302335** | **5382013** | **5060346** | **4252498** | **3310638** | **2480483** | **1800330** | **1350908** | **983095** | **2960665** |
| **Adriatic Sea** | 139454 | 0 | 0 | 0 | 0 | 0 | 0 | 0 | 0 | 0 | 88 | 88 | 0 | 139234 | 0 | 44 | 0 | 0 | 0 | 0 | 0 | 0 | 0 | 0 | 0 | 0 |
| **Aegean Sea** | 191305 | 51 | 0 | 0 | 0 | 51 | 355 | 608 | 862 | 862 | 2433 | 811 | 304 | 171434 | 253 | 1115 | 1622 | 1926 | 3650 | 2281 | 1217 | 963 | 507 | 0 | 0 | 0 |
| **Alboran Sea** | 54711 | 0 | 0 | 0 | 0 | 107 | 160 | 267 | 160 | 588 | 695 | 321 | 53 | 42636 | 0 | 588 | 2832 | 2939 | 2565 | 641 | 53 | 107 | 0 | 0 | 0 | 0 |
| **Andaman or Burma Sea** | 613099 | 0 | 0 | 557 | 2466 | 3977 | 10181 | 17658 | 21953 | 21078 | 15351 | 2943 | 239 | 510810 | 0 | 318 | 398 | 1034 | 1034 | 557 | 477 | 557 | 477 | 636 | 318 | 80 |
| **Arabian Sea** | 4241184 | 37479 | 19855 | 30245 | 47176 | 59566 | 74958 | 70648 | 71571 | 61105 | 42173 | 11005 | 231 | 3284436 | 1077 | 21933 | 60335 | 85501 | 89426 | 67339 | 41250 | 25473 | 17854 | 11236 | 4925 | 4387 |
| **Arafura Sea** | 1025736 | 0 | 0 | 0 | 0 | 0 | 0 | 0 | 0 | 0 | 80 | 0 | 0 | 984144 | 0 | 636 | 1193 | 1352 | 2306 | 4294 | 4533 | 5010 | 5567 | 3738 | 5090 | 7794 |
| **Baffin Bay** | 529960 | 124 | 612 | 1517 | 1999 | 5534 | 9811 | 19844 | 27709 | 28431 | 21498 | 10033 | 1094 | 388520 | 313 | 2682 | 4264 | 2780 | 1152 | 905 | 469 | 228 | 189 | 150 | 72 | 33 |
| **Balearic (Iberian Sea)** | 80078 | 0 | 0 | 0 | 0 | 0 | 0 | 0 | 0 | 0 | 190 | 142 | 0 | 64689 | 0 | 2470 | 6079 | 2802 | 1852 | 855 | 617 | 190 | 142 | 0 | 47 | 0 |
| **Bali Sea** | 39916 | 0 | 0 | 0 | 0 | 0 | 0 | 0 | 0 | 0 | 81 | 0 | 0 | 30443 | 0 | 1862 | 3967 | 2834 | 486 | 162 | 81 | 0 | 0 | 0 | 0 | 0 |
| **Baltic Sea** | 215863 | 0 | 0 | 0 | 0 | 0 | 0 | 0 | 0 | 273 | 13450 | 41043 | 4045 | 155936 | 347 | 769 | 0 | 0 | 0 | 0 | 0 | 0 | 0 | 0 | 0 | 0 |
| **Banda Sea** | 693842 | 0 | 0 | 0 | 162 | 0 | 0 | 162 | 406 | 812 | 406 | 162 | 0 | 451460 | 81 | 1868 | 10478 | 24124 | 37040 | 34846 | 30866 | 26399 | 19576 | 18357 | 12671 | 23962 |
| **Barentsz Sea** | 1408430 | 49715 | 10196 | 10377 | 11242 | 12321 | 13344 | 15108 | 14978 | 15792 | 14983 | 23654 | 6381 | 1121014 | 4380 | 13870 | 10501 | 13056 | 11129 | 8178 | 6110 | 5692 | 4448 | 4883 | 2600 | 4476 |
| **Bass Strait** | 112699 | 0 | 0 | 0 | 0 | 0 | 0 | 0 | 0 | 0 | 440 | 391 | 0 | 111867 | 0 | 0 | 0 | 0 | 0 | 0 | 0 | 0 | 0 | 0 | 0 | 0 |
| **Bay of Bengal** | 2207565 | 24899 | 39137 | 81620 | 124181 | 138265 | 161374 | 164641 | 143400 | 101150 | 43261 | 8870 | 78 | 1156225 | 0 | 1323 | 4980 | 3501 | 2645 | 2801 | 1089 | 1245 | 311 | 233 | 389 | 1945 |
| **Bay of Biscay** | 174437 | 0 | 40 | 0 | 162 | 1010 | 1334 | 3435 | 3920 | 3840 | 1657 | 687 | 81 | 151440 | 0 | 121 | 1293 | 1415 | 1495 | 970 | 808 | 121 | 283 | 242 | 81 | 0 |
| **Bay of Fundy** | 16034 | 0 | 0 | 0 | 0 | 0 | 0 | 43 | 391 | 391 | 391 | 478 | 130 | 12862 | 43 | 521 | 652 | 130 | 0 | 0 | 0 | 0 | 0 | 0 | 0 | 0 |
| **Beaufort Sea** | 431132 | 3016 | 607 | 1005 | 691 | 1508 | 1780 | 2199 | 1299 | 1026 | 482 | 943 | 461 | 393658 | 2786 | 1780 | 1927 | 2514 | 3729 | 1990 | 1445 | 1445 | 670 | 985 | 524 | 2660 |
| **Bering Sea** | 2336912 | 332 | 708 | 1460 | 3717 | 7324 | 12568 | 18321 | 26885 | 34364 | 38236 | 31200 | 12126 | 1913749 | 4160 | 13498 | 17680 | 27460 | 31996 | 33744 | 28212 | 20202 | 15445 | 13608 | 9382 | 20534 |
| **Bismarck Sea** | 345053 | 0 | 0 | 0 | 0 | 163 | 245 | 654 | 980 | 2777 | 2614 | 1470 | 0 | 242534 | 82 | 3758 | 8741 | 14459 | 17155 | 17645 | 12498 | 9313 | 4738 | 3594 | 899 | 735 |
| **Black Sea** | 423026 | 0 | 0 | 0 | 0 | 43 | 87 | 1946 | 20676 | 45676 | 33695 | 8521 | 260 | 271723 | 519 | 5580 | 11246 | 13668 | 6575 | 2249 | 346 | 216 | 0 | 0 | 0 | 0 |
| **Bristol Channel** | 5748 | 0 | 0 | 0 | 0 | 0 | 0 | 0 | 0 | 0 | 0 | 35 | 70 | 5643 | 0 | 0 | 0 | 0 | 0 | 0 | 0 | 0 | 0 | 0 | 0 | 0 |
| **Caribbean Sea** | 2852792 | 990 | 2284 | 5328 | 15605 | 32123 | 59070 | 89366 | 117531 | 118673 | 98577 | 36690 | 1446 | 2040732 | 152 | 35701 | 94999 | 67976 | 20248 | 7079 | 4187 | 2284 | 990 | 76 | 304 | 381 |
| **Celebes Sea** | 457342 | 0 | 0 | 0 | 0 | 0 | 0 | 0 | 0 | 0 | 0 | 82 | 0 | 313191 | 0 | 1561 | 15606 | 26366 | 33923 | 25791 | 19549 | 11417 | 5503 | 2464 | 1068 | 821 |
| **Celtic Sea** | 215080 | 34 | 170 | 0 | 34 | 136 | 545 | 953 | 2689 | 2212 | 1395 | 238 | 34 | 206300 | 34 | 34 | 0 | 68 | 68 | 68 | 68 | 0 | 0 | 0 | 0 | 0 |
| **Ceram Sea** | 161489 | 0 | 0 | 0 | 0 | 0 | 0 | 0 | 0 | 247 | 165 | 412 | 0 | 101694 | 82 | 247 | 3299 | 8330 | 10392 | 9155 | 8083 | 8165 | 5196 | 3217 | 1979 | 825 |
| **Chukchi Sea** | 346259 | 0 | 0 | 0 | 0 | 0 | 0 | 0 | 0 | 0 | 0 | 0 | 40 | 346078 | 141 | 0 | 0 | 0 | 0 | 0 | 0 | 0 | 0 | 0 | 0 | 0 |
| **Coral Sea** | 4125541 | 73 | 73 | 0 | 734 | 3815 | 7410 | 15479 | 25897 | 30519 | 25750 | 8143 | 73 | 3370494 | 367 | 22742 | 102561 | 133520 | 137115 | 95371 | 62285 | 38075 | 24943 | 10784 | 5355 | 3962 |
| **Davis Strait** | 749737 | 1137 | 2022 | 2790 | 5284 | 5801 | 7395 | 10008 | 16487 | 21004 | 21093 | 12591 | 1387 | 629293 | 753 | 3454 | 2849 | 1919 | 1063 | 1018 | 753 | 517 | 177 | 192 | 310 | 443 |
| **East Siberian Sea** | 633057 | 0 | 0 | 0 | 0 | 0 | 0 | 0 | 0 | 0 | 0 | 105 | 271 | 632454 | 226 | 0 | 0 | 0 | 0 | 0 | 0 | 0 | 0 | 0 | 0 | 0 |
| **Eastern China Sea** | 761356 | 0 | 0 | 0 | 0 | 0 | 64 | 192 | 1346 | 2563 | 3653 | 1987 | 64 | 722651 | 192 | 2307 | 6280 | 8907 | 6152 | 3268 | 1025 | 705 | 0 | 0 | 0 | 0 |
| **English Channel** | 81419 | 0 | 0 | 0 | 0 | 0 | 0 | 0 | 0 | 0 | 0 | 0 | 0 | 81080 | 135 | 203 | 0 | 0 | 0 | 0 | 0 | 0 | 0 | 0 | 0 | 0 |
| **Flores Sea** | 102816 | 0 | 0 | 0 | 0 | 0 | 0 | 0 | 0 | 81 | 0 | 81 | 0 | 96758 | 0 | 646 | 1292 | 2342 | 1050 | 404 | 162 | 0 | 0 | 0 | 0 | 0 |
| **Great Australian Bight** | 1326209 | 14675 | 11803 | 10654 | 12325 | 19793 | 23240 | 30552 | 27209 | 25799 | 17600 | 5170 | 261 | 1113391 | 0 | 1149 | 4909 | 4648 | 2298 | 470 | 261 | 0 | 0 | 0 | 0 | 0 |
| **Greenland Sea** | 1186456 | 144548 | 28716 | 28618 | 31902 | 31714 | 34343 | 32918 | 30215 | 25948 | 15808 | 7675 | 1581 | 727783 | 934 | 3972 | 8150 | 6610 | 5652 | 3800 | 4619 | 3997 | 2629 | 1507 | 1360 | 1458 |
| **Gulf of Aden** | 263754 | 0 | 0 | 78 | 0 | 78 | 78 | 311 | 467 | 700 | 700 | 233 | 0 | 183951 | 0 | 1167 | 2100 | 3189 | 5989 | 7078 | 6845 | 7856 | 7156 | 6378 | 6611 | 22790 |
| **Gulf of Alaska** | 415503 | 46 | 46 | 323 | 623 | 1154 | 2654 | 4130 | 5399 | 7292 | 6715 | 5376 | 1338 | 348241 | 346 | 2284 | 5515 | 6392 | 5515 | 3946 | 2792 | 1361 | 1108 | 1038 | 992 | 877 |
| **Gulf of Aqaba** | 3555 | 0 | 0 | 0 | 0 | 0 | 62 | 0 | 374 | 62 | 125 | 0 | 0 | 2931 | 0 | 0 | 0 | 0 | 0 | 0 | 0 | 0 | 0 | 0 | 0 | 0 |
| **Gulf of Boni** | 33237 | 0 | 0 | 0 | 0 | 0 | 0 | 0 | 1059 | 978 | 1629 | 978 | 0 | 28349 | 0 | 163 | 81 | 0 | 0 | 0 | 0 | 0 | 0 | 0 | 0 | 0 |
| **Gulf of Bothnia** | 113419 | 0 | 0 | 0 | 0 | 0 | 0 | 0 | 0 | 231 | 16707 | 34194 | 3352 | 58403 | 195 | 337 | 0 | 0 | 0 | 0 | 0 | 0 | 0 | 0 | 0 | 0 |
| **Gulf of California** | 180703 | 0 | 0 | 0 | 0 | 0 | 0 | 0 | 195 | 910 | 2535 | 1820 | 0 | 157108 | 715 | 2665 | 6435 | 5070 | 2210 | 780 | 130 | 65 | 65 | 0 | 0 | 0 |
| **Gulf of Finland** | 57077 | 467 | 0 | 0 | 0 | 0 | 0 | 0 | 0 | 0 | 0 | 2637 | 793 | 52013 | 723 | 443 | 0 | 0 | 0 | 0 | 0 | 0 | 0 | 0 | 0 | 0 |
| **Gulf of Guinea** | 754844 | 7421 | 1979 | 2556 | 2474 | 2969 | 4370 | 7586 | 9071 | 10390 | 9565 | 2969 | 165 | 575162 | 82 | 2309 | 8246 | 12616 | 13853 | 15585 | 13111 | 9565 | 8988 | 7504 | 5360 | 20945 |
| **Gulf of Mexico** | 1566759 | 5172 | 3627 | 6314 | 6717 | 10278 | 13972 | 15584 | 17532 | 17465 | 16525 | 4904 | 537 | 1355766 | 269 | 6583 | 19413 | 27205 | 19144 | 12024 | 5575 | 1747 | 403 | 0 | 0 | 0 |
| **Gulf of Oman** | 111892 | 0 | 0 | 0 | 0 | 0 | 0 | 0 | 0 | 68 | 0 | 68 | 0 | 78419 | 0 | 679 | 2308 | 5228 | 6518 | 6111 | 5092 | 3055 | 1562 | 1222 | 543 | 1018 |
| **Gulf of Riga** | 18692 | 0 | 0 | 0 | 0 | 0 | 0 | 0 | 0 | 0 | 0 | 1309 | 253 | 16786 | 161 | 184 | 0 | 0 | 0 | 0 | 0 | 0 | 0 | 0 | 0 | 0 |
| **Gulf of St Lawrence** | 290874 | 0 | 0 | 0 | 0 | 363 | 907 | 2722 | 6860 | 10562 | 10635 | 10272 | 1016 | 238100 | 617 | 3121 | 3049 | 1887 | 436 | 181 | 73 | 36 | 0 | 36 | 0 | 0 |
| **Gulf of Suez** | 10438 | 0 | 0 | 0 | 0 | 0 | 0 | 0 | 0 | 0 | 0 | 0 | 0 | 10438 | 0 | 0 | 0 | 0 | 0 | 0 | 0 | 0 | 0 | 0 | 0 | 0 |
| **Gulf of Thailand** | 297927 | 0 | 0 | 0 | 0 | 0 | 0 | 0 | 0 | 0 | 0 | 80 | 0 | 297767 | 0 | 80 | 0 | 0 | 0 | 0 | 0 | 0 | 0 | 0 | 0 | 0 |
| **Gulf of Tomini** | 56739 | 0 | 0 | 0 | 0 | 0 | 0 | 0 | 0 | 0 | 0 | 0 | 0 | 42678 | 0 | 82 | 82 | 2631 | 2796 | 3289 | 2714 | 411 | 905 | 740 | 247 | 164 |
| **Halmahera Sea** | 75295 | 0 | 0 | 82 | 82 | 82 | 0 | 0 | 0 | 163 | 0 | 0 | 0 | 34344 | 0 | 326 | 2692 | 4324 | 9708 | 10197 | 7097 | 3916 | 1958 | 326 | 0 | 0 |
| **Hudson Bay** | 832649 | 0 | 0 | 0 | 0 | 127 | 42 | 762 | 1249 | 1460 | 2222 | 5714 | 2095 | 816437 | 487 | 1143 | 508 | 233 | 63 | 63 | 42 | 0 | 0 | 0 | 0 | 0 |
| **Hudson Strait** | 200039 | 164 | 36 | 509 | 855 | 1128 | 2382 | 4329 | 6493 | 10967 | 10130 | 7438 | 1819 | 151862 | 55 | 382 | 655 | 400 | 109 | 182 | 73 | 36 | 36 | 0 | 0 | 0 |
| **Indian Ocean** | 58230954 | 1502907 | 428862 | 607456 | 852854 | 1172264 | 1524231 | 1880523 | 2016220 | 1854080 | 1147063 | 298086 | 10140 | 40710968 | 1740 | 117853 | 388004 | 561826 | 611134 | 569580 | 486472 | 390837 | 283174 | 215873 | 162588 | 436219 |
| **Inner Seas off the West Coast of Scotland** | 44260 | 0 | 0 | 0 | 0 | 0 | 0 | 103 | 516 | 619 | 800 | 929 | 258 | 39331 | 129 | 594 | 697 | 206 | 0 | 0 | 77 | 0 | 0 | 0 | 0 | 0 |
| **Ionian Sea** | 171778 | 0 | 0 | 0 | 0 | 0 | 0 | 404 | 809 | 2123 | 2275 | 1365 | 202 | 154843 | 0 | 556 | 2174 | 4095 | 2174 | 506 | 152 | 101 | 0 | 0 | 0 | 0 |
| **Irish Sea and St Georges Channel** | 45922 | 0 | 0 | 0 | 0 | 0 | 0 | 0 | 0 | 0 | 29 | 29 | 29 | 45544 | 29 | 174 | 87 | 0 | 0 | 0 | 0 | 0 | 0 | 0 | 0 | 0 |
| **Japan Sea** | 1066307 | 64687 | 31972 | 40464 | 42506 | 43434 | 33596 | 28167 | 21717 | 20464 | 15777 | 10255 | 3480 | 692991 | 93 | 1856 | 6404 | 4965 | 1995 | 928 | 186 | 186 | 93 | 0 | 93 | 0 |
| **Java Sea** | 566693 | 0 | 0 | 0 | 0 | 0 | 0 | 0 | 0 | 0 | 82 | 82 | 0 | 557548 | 0 | 1633 | 2041 | 2695 | 1388 | 653 | 0 | 327 | 0 | 245 | 0 | 0 |
| **Kara Sea** | 896938 | 34 | 92 | 92 | 275 | 332 | 458 | 561 | 1030 | 2495 | 4990 | 15829 | 9282 | 798692 | 6993 | 12670 | 7359 | 5368 | 4910 | 4578 | 4372 | 3834 | 4452 | 3376 | 2003 | 2861 |
| **Kattegat** | 35447 | 0 | 0 | 0 | 0 | 0 | 0 | 0 | 0 | 0 | 26 | 2144 | 1021 | 32257 | 0 | 0 | 0 | 0 | 0 | 0 | 0 | 0 | 0 | 0 | 0 | 0 |
| **Labrador Sea** | 866470 | 203 | 229 | 890 | 2950 | 6409 | 11444 | 16683 | 24160 | 24415 | 23397 | 12945 | 1907 | 715963 | 153 | 1907 | 2950 | 3764 | 2492 | 2696 | 2416 | 2340 | 1984 | 1729 | 1551 | 890 |
| **Laccadive Sea** | 846351 | 9354 | 3145 | 6773 | 7822 | 12176 | 18466 | 18788 | 18949 | 14837 | 9838 | 2984 | 564 | 680000 | 0 | 1693 | 4596 | 5564 | 9273 | 9757 | 4919 | 2338 | 1693 | 1451 | 564 | 806 |
| **Laptev Sea** | 513667 | 22 | 210 | 653 | 2270 | 2447 | 2325 | 1451 | 1772 | 864 | 532 | 10099 | 13167 | 477434 | 277 | 133 | 11 | 0 | 0 | 0 | 0 | 0 | 0 | 0 | 0 | 0 |
| **Ligurian Sea** | 16949 | 0 | 0 | 0 | 0 | 0 | 0 | 0 | 0 | 0 | 0 | 0 | 0 | 8983 | 42 | 127 | 1059 | 1695 | 2330 | 1525 | 1102 | 85 | 0 | 0 | 0 | 0 |
| **Makassar Strait** | 198117 | 0 | 0 | 0 | 0 | 0 | 0 | 0 | 0 | 165 | 576 | 0 | 0 | 137266 | 82 | 1812 | 9634 | 12681 | 15480 | 9634 | 6258 | 3541 | 823 | 165 | 0 | 0 |
| **Malacca Strait** | 195534 | 0 | 0 | 0 | 0 | 0 | 0 | 0 | 0 | 82 | 0 | 0 | 0 | 190609 | 0 | 246 | 82 | 410 | 739 | 903 | 821 | 1067 | 328 | 164 | 82 | 0 |
| **Mediterranean Sea - Eastern Basin** | 1173640 | 0 | 0 | 0 | 615 | 1006 | 3354 | 7603 | 13250 | 13977 | 14088 | 4640 | 615 | 1087992 | 168 | 1454 | 6597 | 6932 | 5255 | 3634 | 1398 | 895 | 112 | 56 | 0 | 0 |
| **Mediterranean Sea - Western Basin** | 476707 | 0 | 0 | 0 | 0 | 0 | 340 | 1458 | 2625 | 3840 | 4131 | 1069 | 0 | 437289 | 0 | 1653 | 7048 | 5881 | 6075 | 2770 | 1069 | 729 | 535 | 49 | 49 | 97 |
| **Molukka Sea** | 221671 | 0 | 0 | 0 | 0 | 0 | 0 | 0 | 0 | 82 | 0 | 0 | 0 | 149014 | 82 | 493 | 1479 | 3945 | 4603 | 8877 | 11836 | 12822 | 10192 | 7973 | 5178 | 5096 |
| **Mozambique Channel** | 1394283 | 0 | 0 | 0 | 0 | 0 | 0 | 0 | 218 | 2030 | 5729 | 2973 | 0 | 1008005 | 218 | 5438 | 30311 | 63594 | 83753 | 82810 | 59896 | 29368 | 11022 | 4641 | 2538 | 1740 |
| **North Atlantic Ocean** | 34507128 | 419127 | 346814 | 508040 | 652615 | 842828 | 1036896 | 1181779 | 1304664 | 1327946 | 873511 | 254149 | 18502 | 22125022 | 14596 | 182401 | 452123 | 534920 | 487020 | 414296 | 344655 | 268334 | 219046 | 174538 | 142776 | 380529 |
| **North Pacific Ocean** | 65154681 | 216248 | 103312 | 139199 | 205631 | 325583 | 504828 | 800050 | 1221558 | 1787603 | 1798033 | 562506 | 25021 | 48626433 | 2421 | 356751 | 1180767 | 1455252 | 1330768 | 1150096 | 920188 | 729768 | 540465 | 402384 | 285040 | 484774 |
| **North Sea** | 524493 | 0 | 0 | 0 | 0 | 0 | 99 | 197 | 444 | 913 | 963 | 1234 | 247 | 514670 | 568 | 987 | 1580 | 1654 | 518 | 296 | 74 | 49 | 0 | 0 | 0 | 0 |
| **Norwegian Sea** | 1437096 | 2824 | 2960 | 5469 | 12365 | 22809 | 34963 | 53321 | 52933 | 48902 | 30618 | 9604 | 903 | 1068538 | 703 | 4345 | 11619 | 16857 | 16626 | 15745 | 10003 | 7851 | 3453 | 1742 | 976 | 966 |
| **Persian Gulf** | 244657 | 0 | 0 | 0 | 0 | 0 | 0 | 0 | 0 | 0 | 0 | 0 | 0 | 243999 | 132 | 198 | 263 | 66 | 0 | 0 | 0 | 0 | 0 | 0 | 0 | 0 |
| **Philippine Sea** | 5641996 | 15865 | 5625 | 8870 | 10817 | 16369 | 20984 | 30215 | 40238 | 47594 | 39805 | 12764 | 2091 | 3854210 | 288 | 34037 | 173644 | 319309 | 366542 | 293061 | 182153 | 93745 | 36344 | 20912 | 10023 | 6490 |
| **Red Sea** | 449811 | 0 | 0 | 0 | 0 | 72 | 573 | 1864 | 3799 | 7025 | 9606 | 3656 | 215 | 383791 | 430 | 4229 | 4659 | 5233 | 5376 | 5663 | 4301 | 3441 | 2294 | 1290 | 789 | 1505 |
| **Rio de La Plata** | 31797 | 0 | 0 | 0 | 0 | 0 | 0 | 0 | 0 | 0 | 0 | 0 | 0 | 31797 | 0 | 0 | 0 | 0 | 0 | 0 | 0 | 0 | 0 | 0 | 0 | 0 |
| **Savu Sea** | 106234 | 0 | 0 | 0 | 0 | 0 | 0 | 0 | 0 | 0 | 0 | 239 | 0 | 97554 | 80 | 876 | 1274 | 2071 | 2708 | 1195 | 159 | 0 | 0 | 80 | 0 | 0 |
| **Sea of Azov** | 39547 | 0 | 0 | 0 | 0 | 0 | 0 | 0 | 0 | 0 | 0 | 0 | 238 | 38122 | 792 | 396 | 0 | 0 | 0 | 0 | 0 | 0 | 0 | 0 | 0 | 0 |
| **Sea of Marmara** | 11675 | 0 | 0 | 0 | 0 | 0 | 0 | 0 | 0 | 0 | 0 | 335 | 0 | 6890 | 96 | 526 | 766 | 766 | 1292 | 622 | 144 | 144 | 96 | 0 | 0 | 0 |
| **Sea of Okhotsk** | 1613208 | 5320 | 5264 | 9092 | 12976 | 17311 | 24630 | 27135 | 34003 | 32568 | 30147 | 20013 | 4813 | 1247139 | 4419 | 15144 | 23532 | 25531 | 21421 | 16045 | 12948 | 9739 | 4476 | 3322 | 1351 | 4870 |
| **Seto Naikai or Inland Sea** | 18131 | 0 | 0 | 0 | 0 | 0 | 0 | 0 | 0 | 0 | 57 | 57 | 0 | 17961 | 0 | 0 | 57 | 0 | 0 | 0 | 0 | 0 | 0 | 0 | 0 | 0 |
| **Singapore Strait** | 2684 | 0 | 0 | 0 | 0 | 0 | 0 | 0 | 0 | 0 | 0 | 0 | 0 | 2684 | 0 | 0 | 0 | 0 | 0 | 0 | 0 | 0 | 0 | 0 | 0 | 0 |
| **Skagerrak** | 32040 | 0 | 0 | 0 | 0 | 0 | 0 | 0 | 0 | 47 | 605 | 1233 | 70 | 29225 | 23 | 279 | 465 | 93 | 0 | 0 | 0 | 0 | 0 | 0 | 0 | 0 |
| **Solomon Sea** | 744334 | 0 | 0 | 80 | 722 | 882 | 2647 | 3449 | 5534 | 5294 | 5133 | 2085 | 0 | 543973 | 0 | 6818 | 26228 | 31602 | 30479 | 25827 | 20854 | 15159 | 7700 | 6417 | 2406 | 1043 |
| **South Atlantic Ocean** | 40501812 | 1221568 | 294582 | 351956 | 450299 | 622966 | 871539 | 1217862 | 1521092 | 1514766 | 1064913 | 274716 | 21645 | 27719650 | 2570 | 144301 | 454845 | 560501 | 499371 | 403055 | 304367 | 225594 | 174644 | 130316 | 91226 | 363471 |
| **South China Sea** | 3362904 | 0 | 0 | 0 | 155 | 777 | 5825 | 13125 | 20037 | 22522 | 23687 | 8154 | 0 | 2849250 | 388 | 22056 | 68575 | 78982 | 73779 | 70905 | 42947 | 27570 | 17163 | 10096 | 3883 | 3029 |
| **South Pacific Ocean** | 76681173 | 2418405 | 855418 | 1116223 | 1442441 | 1823739 | 2204300 | 2457357 | 2551179 | 2424941 | 1838076 | 555134 | 35737 | 52220665 | 1792 | 266076 | 817573 | 888519 | 710099 | 538530 | 405756 | 296489 | 223645 | 165402 | 118016 | 305661 |
| **Southern Ocean** | 21695157 | 3224742 | 356650 | 385074 | 400074 | 382687 | 366088 | 316314 | 269365 | 183197 | 103489 | 33153 | 2781 | 14033759 | 1139 | 19686 | 51350 | 72394 | 88314 | 95934 | 103205 | 102460 | 95475 | 89059 | 82840 | 835928 |
| **Strait of Gibraltar** | 1664 | 0 | 0 | 0 | 0 | 0 | 0 | 0 | 0 | 0 | 0 | 0 | 0 | 1553 | 0 | 0 | 0 | 111 | 0 | 0 | 0 | 0 | 0 | 0 | 0 | 0 |
| **Sulu Sea** | 337029 | 0 | 0 | 0 | 0 | 0 | 161 | 161 | 0 | 241 | 804 | 322 | 80 | 302546 | 0 | 402 | 3778 | 7475 | 8922 | 5707 | 3376 | 2009 | 804 | 241 | 0 | 0 |
| **Tasman Sea** | 3344624 | 48 | 95 | 334 | 1860 | 5341 | 8583 | 15021 | 22698 | 29422 | 24463 | 9966 | 48 | 2582464 | 48 | 27133 | 88171 | 126892 | 125890 | 95944 | 77155 | 49116 | 26656 | 14973 | 7916 | 4387 |
| **The Coastal Waters of Southeast Alaska and British Columbia** | 124532 | 0 | 0 | 0 | 0 | 0 | 0 | 65 | 196 | 720 | 1506 | 3831 | 589 | 104492 | 360 | 5108 | 3471 | 2358 | 1048 | 491 | 229 | 65 | 0 | 0 | 0 | 0 |
| **The Northwestern Passages** | 1062173 | 471 | 404 | 488 | 1345 | 2741 | 4490 | 8946 | 11569 | 11636 | 11064 | 7970 | 2590 | 914047 | 4826 | 18043 | 13133 | 15453 | 14259 | 10846 | 5230 | 1967 | 437 | 219 | 0 | 0 |
| **Timor Sea** | 434186 | 0 | 0 | 0 | 0 | 0 | 0 | 0 | 0 | 0 | 0 | 0 | 0 | 359811 | 159 | 1032 | 3175 | 8731 | 16590 | 19050 | 15637 | 7620 | 1349 | 953 | 79 | 0 |
| **Tyrrhenian Sea** | 217465 | 0 | 0 | 0 | 0 | 0 | 0 | 0 | 0 | 48 | 143 | 0 | 0 | 176803 | 238 | 2708 | 10023 | 11496 | 8835 | 4845 | 1663 | 523 | 48 | 95 | 0 | 0 |
| **White Sea** | 90531 | 0 | 0 | 0 | 0 | 0 | 0 | 81 | 40 | 135 | 2874 | 7259 | 1875 | 73126 | 580 | 1727 | 1511 | 769 | 351 | 175 | 13 | 0 | 0 | 0 | 0 | 13 |
| **Yellow Sea** | 408198 | 53 | 0 | 0 | 0 | 0 | 0 | 0 | 0 | 106 | 0 | 0 | 0 | 407879 | 106 | 53 | 0 | 0 | 0 | 0 | 0 | 0 | 0 | 0 | 0 | 0 |

**Table S6| Relative percentage changes in the depth of the photic zone (*Z_photic_*) across the global ocean and International Hydrographic Office (IHO) regions between 2003 and 2022 when illuminated by sunlight.** *Z_photic_* was calculated using Beers Law (see methods). The annual average of monthly maximum zenith surface solar irradiance was modelled at 9km resolution. The depth of the photic zone was defined as the minimum irradiance at 520nm that elicits diel vertical migration in Calanus copepods. *K_d_*(490) was taken as the modelled values for 2003 and 2022 given by quantile regression on the median, with serial autocorrelation removed using ARIMA models.

| IHO region | Total Area (km^2^) | Area where *Z_photic_* is decreasing (km^2^) | | | | | | | | | | | | Area no change in *Z_photic_* (km^2^) | Area where *Z_photic_* is increasing (km^2^) | | | | | | | | | | | |
| --- | --- | --- | --- | --- | --- | --- | --- | --- | --- | --- | --- | --- | --- | --- | --- | --- | --- | --- | --- | --- | --- | --- | --- | --- | --- | --- |
|  |  | 100% | 90-100% | 80-90% | 70-80% | 60-70% | 50-60% | 40-50% | 30-40% | 20-30% | 10-20% | 1-10% | 0-1% |  | 0-1% | 1-10% | 10-20% | 20-30% | 30-40% | 40-50% | 50-60% | 60-70% | 70-80% | 80-90% | 90-100% | 100% |
| **Global Ocean** | **359491235** | **1483** | **679** | **4906** | **16873** | **57811** | **287496** | **985519** | **2725789** | **7049451** | **21316935** | **37706845** | **875964** | **255077013** | **346245** | **18424326** | **8889328** | **2395263** | **1026901** | **664644** | **478773** | **308672** | **215105** | **156091** | **113515** | **365608** |
| **Adriatic Sea** | 139454 | 0 | 0 | 0 | 0 | 0 | 0 | 0 | 0 | 0 | 0 | 132 | 44 | 139234 | 0 | 44 | 0 | 0 | 0 | 0 | 0 | 0 | 0 | 0 | 0 | 0 |
| **Adriatic Sea** | 139454 | 0 | 0 | 0 | 0 | 0 | 0 | 0 | 0 | 0 | 88 | 88 | 0 | 139234 | 0 | 44 | 0 | 0 | 0 | 0 | 0 | 0 | 0 | 0 | 0 | 0 |
| **Aegean Sea** | 191305 | 0 | 0 | 0 | 0 | 0 | 0 | 0 | 0 | 0 | 304 | 5525 | 507 | 171434 | 608 | 8212 | 3802 | 912 | 0 | 0 | 0 | 0 | 0 | 0 | 0 | 0 |
| **Alboran Sea** | 54711 | 0 | 0 | 0 | 0 | 0 | 0 | 0 | 0 | 107 | 534 | 1603 | 107 | 42636 | 160 | 5824 | 3526 | 107 | 53 | 53 | 0 | 0 | 0 | 0 | 0 | 0 |
| **Andaman or Burma Sea** | 613099 | 0 | 0 | 0 | 0 | 0 | 0 | 0 | 0 | 0 | 14238 | 81768 | 398 | 510810 | 0 | 2386 | 1909 | 1511 | 0 | 0 | 0 | 80 | 0 | 0 | 0 | 0 |
| **Arabian Sea** | 4241184 | 0 | 0 | 0 | 0 | 0 | 462 | 7773 | 27474 | 87579 | 218947 | 181699 | 2078 | 3284436 | 4310 | 245729 | 106818 | 31938 | 15853 | 10312 | 6465 | 2924 | 2001 | 1077 | 847 | 2463 |
| **Arafura Sea** | 1025736 | 0 | 0 | 0 | 0 | 0 | 0 | 0 | 80 | 0 | 0 | 0 | 0 | 984144 | 239 | 3976 | 12247 | 12009 | 9384 | 3261 | 398 | 0 | 0 | 0 | 0 | 0 |
| **Baffin Bay** | 529960 | 0 | 0 | 0 | 0 | 0 | 20 | 39 | 3861 | 16517 | 58510 | 47051 | 2207 | 388520 | 612 | 5241 | 4238 | 1126 | 723 | 508 | 469 | 130 | 130 | 7 | 0 | 52 |
| **Balearic (Iberian Sea)** | 80078 | 0 | 0 | 0 | 0 | 0 | 0 | 0 | 0 | 0 | 0 | 332 | 0 | 64689 | 190 | 12776 | 1995 | 95 | 0 | 0 | 0 | 0 | 0 | 0 | 0 | 0 |
| **Bali Sea** | 39916 | 0 | 0 | 0 | 0 | 0 | 0 | 0 | 0 | 0 | 0 | 81 | 0 | 30443 | 162 | 8906 | 243 | 81 | 0 | 0 | 0 | 0 | 0 | 0 | 0 | 0 |
| **Baltic Sea** | 215863 | 0 | 0 | 0 | 0 | 0 | 0 | 298 | 6353 | 18859 | 22284 | 9479 | 1539 | 155936 | 74 | 769 | 273 | 0 | 0 | 0 | 0 | 0 | 0 | 0 | 0 | 0 |
| **Banda Sea** | 693842 | 0 | 0 | 0 | 0 | 0 | 0 | 0 | 0 | 81 | 569 | 1462 | 0 | 451460 | 244 | 76516 | 107463 | 45000 | 8854 | 1625 | 81 | 81 | 0 | 244 | 0 | 162 |
| **Barentsz Sea** | 1408430 | 0 | 0 | 0 | 0 | 79 | 14667 | 33974 | 37021 | 36371 | 30673 | 35071 | 10236 | 1121014 | 7698 | 16673 | 12960 | 11287 | 9055 | 7766 | 5584 | 4205 | 3386 | 2764 | 2300 | 5646 |
| **Bass Strait** | 112699 | 0 | 0 | 0 | 0 | 0 | 0 | 0 | 0 | 0 | 0 | 832 | 0 | 111867 | 0 | 0 | 0 | 0 | 0 | 0 | 0 | 0 | 0 | 0 | 0 | 0 |
| **Bay of Bengal** | 2207565 | 0 | 0 | 0 | 0 | 0 | 0 | 0 | 0 | 9337 | 480230 | 539831 | 1478 | 1156225 | 156 | 12527 | 4591 | 934 | 389 | 467 | 545 | 233 | 78 | 233 | 311 | 0 |
| **Bay of Biscay** | 174437 | 0 | 0 | 0 | 0 | 0 | 0 | 0 | 0 | 647 | 8124 | 7235 | 162 | 151440 | 0 | 2506 | 3193 | 768 | 364 | 0 | 0 | 0 | 0 | 0 | 0 | 0 |
| **Bay of Fundy** | 16034 | 0 | 0 | 0 | 0 | 0 | 0 | 43 | 0 | 348 | 826 | 478 | 130 | 12862 | 0 | 261 | 565 | 304 | 174 | 43 | 0 | 0 | 0 | 0 | 0 | 0 |
| **Beaufort Sea** | 431132 | 0 | 0 | 0 | 0 | 0 | 63 | 1341 | 4231 | 3414 | 4022 | 1550 | 398 | 393658 | 1864 | 3121 | 2116 | 1969 | 1634 | 2158 | 2158 | 2011 | 754 | 1026 | 314 | 3331 |
| **Bering Sea** | 2336912 | 0 | 0 | 22 | 0 | 22 | 44 | 509 | 4160 | 23809 | 64745 | 82535 | 11396 | 1913749 | 3894 | 26265 | 46512 | 48547 | 34452 | 23831 | 16905 | 9493 | 6837 | 5842 | 4160 | 9183 |
| **Bismarck Sea** | 345053 | 0 | 0 | 0 | 0 | 0 | 0 | 0 | 0 | 0 | 654 | 7760 | 490 | 242534 | 408 | 57591 | 34473 | 1144 | 0 | 0 | 0 | 0 | 0 | 0 | 0 | 0 |
| **Black Sea** | 423026 | 0 | 0 | 0 | 0 | 0 | 0 | 0 | 0 | 173 | 37934 | 72364 | 433 | 271723 | 822 | 17604 | 16610 | 4109 | 779 | 260 | 216 | 0 | 0 | 0 | 0 | 0 |
| **Bristol Channel** | 5748 | 0 | 0 | 0 | 0 | 0 | 0 | 0 | 0 | 0 | 0 | 35 | 70 | 5643 | 0 | 0 | 0 | 0 | 0 | 0 | 0 | 0 | 0 | 0 | 0 | 0 |
| **Caribbean Sea** | 2852792 | 0 | 0 | 0 | 0 | 76 | 457 | 1675 | 3425 | 8145 | 93705 | 460000 | 10200 | 2040732 | 13321 | 213215 | 6851 | 533 | 228 | 0 | 76 | 0 | 76 | 0 | 0 | 76 |
| **Celebes Sea** | 457342 | 0 | 0 | 0 | 0 | 0 | 0 | 0 | 0 | 0 | 0 | 82 | 0 | 313191 | 329 | 102754 | 38523 | 2136 | 329 | 0 | 0 | 0 | 0 | 0 | 0 | 0 |
| **Celtic Sea** | 215080 | 0 | 0 | 0 | 0 | 0 | 0 | 0 | 136 | 238 | 4901 | 3063 | 102 | 206300 | 68 | 68 | 136 | 68 | 0 | 0 | 0 | 0 | 0 | 0 | 0 | 0 |
| **Ceram Sea** | 161489 | 0 | 0 | 0 | 0 | 0 | 0 | 0 | 0 | 0 | 165 | 577 | 82 | 101694 | 82 | 17650 | 26228 | 13691 | 1237 | 82 | 0 | 0 | 0 | 0 | 0 | 0 |
| **Chukchi Sea** | 346259 | 0 | 0 | 0 | 0 | 0 | 0 | 0 | 0 | 0 | 0 | 10 | 30 | 346078 | 40 | 101 | 0 | 0 | 0 | 0 | 0 | 0 | 0 | 0 | 0 | 0 |
| **Coral Sea** | 4125541 | 0 | 0 | 0 | 0 | 0 | 0 | 0 | 0 | 0 | 2421 | 110997 | 4548 | 3370494 | 7997 | 560856 | 67640 | 587 | 0 | 0 | 0 | 0 | 0 | 0 | 0 | 0 |
| **Davis Strait** | 749737 | 0 | 0 | 0 | 30 | 89 | 517 | 5063 | 12059 | 13299 | 30111 | 42805 | 3026 | 629293 | 1092 | 5506 | 1993 | 1284 | 1314 | 708 | 472 | 310 | 384 | 44 | 74 | 266 |
| **East Siberian Sea** | 633057 | 0 | 0 | 0 | 0 | 0 | 0 | 0 | 15 | 45 | 30 | 105 | 181 | 632454 | 121 | 105 | 0 | 0 | 0 | 0 | 0 | 0 | 0 | 0 | 0 | 0 |
| **Eastern China Sea** | 761356 | 0 | 0 | 0 | 0 | 0 | 0 | 0 | 0 | 192 | 320 | 8907 | 449 | 722651 | 513 | 26658 | 1602 | 0 | 0 | 0 | 64 | 0 | 0 | 0 | 0 | 0 |
| **English Channel** | 81419 | 0 | 0 | 0 | 0 | 0 | 0 | 0 | 0 | 0 | 0 | 0 | 0 | 81080 | 68 | 203 | 34 | 34 | 0 | 0 | 0 | 0 | 0 | 0 | 0 | 0 |
| **Flores Sea** | 102816 | 0 | 0 | 0 | 0 | 0 | 0 | 0 | 0 | 0 | 0 | 81 | 81 | 96758 | 162 | 5573 | 162 | 0 | 0 | 0 | 0 | 0 | 0 | 0 | 0 | 0 |
| **Great Australian Bight** | 1326209 | 0 | 0 | 0 | 0 | 0 | 0 | 0 | 0 | 10654 | 93431 | 93953 | 1045 | 1113391 | 157 | 11333 | 2246 | 0 | 0 | 0 | 0 | 0 | 0 | 0 | 0 | 0 |
| **Greenland Sea** | 1186456 | 0 | 0 | 786 | 3448 | 8821 | 32558 | 77778 | 92922 | 95314 | 71340 | 28659 | 2359 | 727783 | 1155 | 9526 | 9796 | 7044 | 6192 | 4267 | 3203 | 1679 | 672 | 541 | 205 | 410 |
| **Gulf of Aden** | 263754 | 0 | 0 | 0 | 0 | 0 | 0 | 0 | 0 | 233 | 856 | 1556 | 0 | 183951 | 156 | 3889 | 13923 | 15867 | 14778 | 9256 | 7778 | 3422 | 2411 | 1789 | 933 | 2956 |
| **Gulf of Alaska** | 415503 | 0 | 0 | 0 | 0 | 46 | 185 | 600 | 2584 | 7061 | 13614 | 9368 | 1638 | 348241 | 346 | 4707 | 11145 | 6692 | 3484 | 2146 | 1384 | 1200 | 392 | 277 | 46 | 346 |
| **Gulf of Aqaba** | 3555 | 0 | 0 | 0 | 0 | 0 | 0 | 0 | 0 | 0 | 0 | 624 | 0 | 2931 | 0 | 0 | 0 | 0 | 0 | 0 | 0 | 0 | 0 | 0 | 0 | 0 |
| **Gulf of Boni** | 33237 | 0 | 0 | 0 | 0 | 0 | 0 | 0 | 0 | 0 | 0 | 4643 | 0 | 28349 | 81 | 163 | 0 | 0 | 0 | 0 | 0 | 0 | 0 | 0 | 0 | 0 |
| **Gulf of Bothnia** | 113419 | 0 | 0 | 0 | 0 | 0 | 71 | 1915 | 9293 | 22311 | 15838 | 4168 | 887 | 58403 | 0 | 124 | 195 | 106 | 89 | 18 | 0 | 0 | 0 | 0 | 0 | 0 |
| **Gulf of California** | 180703 | 0 | 0 | 0 | 0 | 0 | 0 | 0 | 65 | 260 | 585 | 4550 | 0 | 157108 | 845 | 8450 | 6955 | 1560 | 260 | 0 | 0 | 0 | 65 | 0 | 0 | 0 |
| **Gulf of Finland** | 57077 | 467 | 0 | 0 | 0 | 0 | 0 | 0 | 397 | 1400 | 1027 | 513 | 93 | 52013 | 47 | 373 | 490 | 187 | 23 | 23 | 23 | 0 | 0 | 0 | 0 | 0 |
| **Gulf of Guinea** | 754844 | 0 | 0 | 165 | 412 | 1237 | 1484 | 3711 | 3958 | 6762 | 12781 | 30758 | 247 | 575162 | 82 | 38921 | 36777 | 16657 | 7092 | 4453 | 2309 | 1814 | 1484 | 1237 | 495 | 6844 |
| **Gulf of Mexico** | 1566759 | 0 | 0 | 0 | 0 | 0 | 537 | 1881 | 2485 | 5038 | 31236 | 75973 | 1478 | 1355766 | 2150 | 85445 | 4299 | 0 | 134 | 202 | 67 | 0 | 0 | 0 | 0 | 67 |
| **Gulf of Oman** | 111892 | 0 | 0 | 0 | 0 | 0 | 0 | 0 | 0 | 0 | 68 | 68 | 0 | 78419 | 0 | 679 | 2308 | 4345 | 5635 | 6111 | 5160 | 3327 | 1969 | 1222 | 679 | 1901 |
| **Gulf of Riga** | 18692 | 0 | 0 | 0 | 0 | 0 | 0 | 0 | 0 | 138 | 1056 | 321 | 46 | 16786 | 0 | 115 | 184 | 46 | 0 | 0 | 0 | 0 | 0 | 0 | 0 | 0 |
| **Gulf of St Lawrence** | 290874 | 0 | 0 | 0 | 0 | 36 | 73 | 181 | 2650 | 8457 | 20471 | 10744 | 726 | 238100 | 617 | 3775 | 3049 | 1089 | 327 | 436 | 0 | 73 | 0 | 0 | 0 | 73 |
| **Gulf of Suez** | 10438 | 0 | 0 | 0 | 0 | 0 | 0 | 0 | 0 | 0 | 0 | 0 | 0 | 10438 | 0 | 0 | 0 | 0 | 0 | 0 | 0 | 0 | 0 | 0 | 0 | 0 |
| **Gulf of Thailand** | 297927 | 0 | 0 | 0 | 0 | 0 | 0 | 0 | 0 | 80 | 0 | 0 | 0 | 297767 | 0 | 0 | 0 | 80 | 0 | 0 | 0 | 0 | 0 | 0 | 0 | 0 |
| **Gulf of Tomini** | 56739 | 0 | 0 | 0 | 0 | 0 | 0 | 0 | 0 | 0 | 0 | 0 | 0 | 42678 | 0 | 9621 | 3783 | 658 | 0 | 0 | 0 | 0 | 0 | 0 | 0 | 0 |
| **Halmahera Sea** | 75295 | 0 | 0 | 0 | 0 | 0 | 0 | 0 | 245 | 0 | 82 | 82 | 0 | 34344 | 163 | 13868 | 24310 | 2203 | 0 | 0 | 0 | 0 | 0 | 0 | 0 | 0 |
| **Hudson Bay** | 832649 | 0 | 0 | 0 | 0 | 0 | 0 | 190 | 741 | 1735 | 4063 | 4423 | 2518 | 816437 | 614 | 550 | 466 | 317 | 127 | 169 | 106 | 63 | 42 | 0 | 42 | 42 |
| **Hudson Strait** | 200039 | 0 | 0 | 0 | 0 | 73 | 0 | 436 | 1564 | 6347 | 18296 | 16587 | 2946 | 151862 | 109 | 709 | 709 | 255 | 73 | 73 | 0 | 0 | 0 | 0 | 0 | 0 |
| **Indian Ocean** | 58230954 | 0 | 0 | 0 | 0 | 0 | 0 | 28979 | 313048 | 1003960 | 4522491 | 7346929 | 79281 | 40710968 | 26891 | 2346466 | 1409112 | 334918 | 74062 | 16602 | 6561 | 4076 | 2535 | 1044 | 1392 | 1640 |
| **Inner Seas off the West Coast of Scotland** | 44260 | 0 | 0 | 0 | 0 | 0 | 0 | 0 | 361 | 826 | 1161 | 594 | 284 | 39331 | 129 | 516 | 439 | 181 | 232 | 52 | 0 | 0 | 52 | 0 | 0 | 103 |
| **Ionian Sea** | 171778 | 0 | 0 | 0 | 0 | 0 | 0 | 51 | 101 | 51 | 51 | 6420 | 506 | 154843 | 101 | 9554 | 101 | 0 | 0 | 0 | 0 | 0 | 0 | 0 | 0 | 0 |
| **Irish Sea and St Georges Channel** | 45922 | 0 | 0 | 0 | 0 | 0 | 0 | 0 | 0 | 0 | 29 | 29 | 29 | 45544 | 58 | 174 | 58 | 0 | 0 | 0 | 0 | 0 | 0 | 0 | 0 | 0 |
| **Japan Sea** | 1066307 | 0 | 0 | 0 | 0 | 46 | 974 | 13550 | 46589 | 140788 | 108167 | 42320 | 4084 | 692991 | 232 | 11137 | 4316 | 696 | 93 | 186 | 0 | 0 | 93 | 46 | 0 | 0 |
| **Java Sea** | 566693 | 0 | 0 | 0 | 0 | 0 | 0 | 0 | 0 | 0 | 0 | 163 | 0 | 557548 | 327 | 7431 | 735 | 327 | 163 | 0 | 0 | 0 | 0 | 0 | 0 | 0 |
| **Kara Sea** | 896938 | 0 | 0 | 0 | 0 | 0 | 69 | 721 | 1545 | 5711 | 9374 | 12498 | 5551 | 798692 | 5265 | 9557 | 7600 | 6627 | 5471 | 4578 | 4143 | 4074 | 2667 | 2117 | 1717 | 8962 |
| **Kattegat** | 35447 | 0 | 0 | 0 | 0 | 0 | 0 | 51 | 179 | 868 | 1046 | 817 | 230 | 32257 | 0 | 0 | 0 | 0 | 0 | 0 | 0 | 0 | 0 | 0 | 0 | 0 |
| **Labrador Sea** | 866470 | 0 | 0 | 0 | 25 | 25 | 76 | 483 | 2441 | 20066 | 56739 | 42904 | 2874 | 715963 | 280 | 4858 | 6434 | 4552 | 3789 | 2976 | 1221 | 203 | 102 | 178 | 51 | 229 |
| **Laccadive Sea** | 846351 | 0 | 0 | 0 | 0 | 0 | 161 | 726 | 2016 | 10321 | 51123 | 58058 | 1290 | 680000 | 242 | 27497 | 11289 | 1129 | 726 | 968 | 403 | 161 | 81 | 0 | 81 | 81 |
| **Laptev Sea** | 513667 | 0 | 0 | 0 | 0 | 0 | 0 | 687 | 6445 | 6445 | 6157 | 8770 | 7309 | 477434 | 177 | 144 | 78 | 22 | 0 | 0 | 0 | 0 | 0 | 0 | 0 | 0 |
| **Ligurian Sea** | 16949 | 0 | 0 | 0 | 0 | 0 | 0 | 0 | 0 | 0 | 0 | 0 | 0 | 8983 | 169 | 4958 | 2797 | 42 | 0 | 0 | 0 | 0 | 0 | 0 | 0 | 0 |
| **Makassar Strait** | 198117 | 0 | 0 | 0 | 0 | 0 | 0 | 0 | 0 | 0 | 0 | 741 | 0 | 137266 | 329 | 37384 | 21409 | 906 | 82 | 0 | 0 | 0 | 0 | 0 | 0 | 0 |
| **Malacca Strait** | 195534 | 0 | 0 | 0 | 0 | 0 | 0 | 0 | 0 | 0 | 0 | 82 | 0 | 190609 | 0 | 903 | 3201 | 739 | 0 | 0 | 0 | 0 | 0 | 0 | 0 | 0 |
| **Mediterranean Sea - Eastern Basin** | 1173640 | 0 | 0 | 0 | 0 | 0 | 0 | 0 | 0 | 0 | 0 | 56130 | 3019 | 1087992 | 1118 | 24431 | 895 | 56 | 0 | 0 | 0 | 0 | 0 | 0 | 0 | 0 |
| **Mediterranean Sea - Western Basin** | 476707 | 0 | 0 | 0 | 0 | 0 | 0 | 0 | 0 | 0 | 1264 | 12005 | 194 | 437289 | 243 | 20171 | 5055 | 437 | 49 | 0 | 0 | 0 | 0 | 0 | 0 | 0 |
| **Molukka Sea** | 221671 | 0 | 0 | 0 | 0 | 0 | 0 | 0 | 0 | 0 | 0 | 82 | 0 | 149014 | 247 | 13397 | 38301 | 16274 | 4192 | 164 | 0 | 0 | 0 | 0 | 0 | 0 |
| **Mozambique Channel** | 1394283 | 0 | 0 | 0 | 0 | 0 | 0 | 0 | 0 | 0 | 0 | 10224 | 725 | 1008005 | 1378 | 294476 | 76284 | 2901 | 218 | 0 | 73 | 0 | 0 | 0 | 0 | 0 |
| **North Atlantic Ocean** | 34507128 | 0 | 0 | 0 | 103 | 308 | 1696 | 12489 | 223363 | 1392704 | 2837266 | 4221901 | 77041 | 22125022 | 66762 | 1826941 | 753401 | 247313 | 190573 | 188157 | 144626 | 86498 | 47283 | 23950 | 14853 | 24875 |
| **North Pacific Ocean** | 65154681 | 0 | 0 | 0 | 1863 | 14342 | 44392 | 106727 | 192159 | 328687 | 1391117 | 5335118 | 275169 | 48626433 | 71896 | 5073484 | 3029028 | 465340 | 103995 | 45944 | 23220 | 11176 | 6519 | 3166 | 1801 | 3104 |
| **North Sea** | 524493 | 0 | 0 | 0 | 0 | 0 | 25 | 123 | 444 | 790 | 1357 | 1185 | 173 | 514670 | 197 | 1333 | 1925 | 1308 | 469 | 247 | 0 | 25 | 0 | 0 | 0 | 222 |
| **Norwegian Sea** | 1437096 | 0 | 0 | 0 | 73 | 157 | 430 | 2288 | 8051 | 61729 | 144587 | 59042 | 1312 | 1068538 | 903 | 15933 | 30786 | 22630 | 11389 | 4440 | 2026 | 1291 | 714 | 252 | 168 | 357 |
| **Persian Gulf** | 244657 | 0 | 0 | 0 | 0 | 0 | 0 | 0 | 0 | 0 | 0 | 0 | 0 | 243999 | 132 | 198 | 132 | 198 | 0 | 0 | 0 | 0 | 0 | 0 | 0 | 0 |
| **Philippine Sea** | 5641996 | 0 | 0 | 0 | 0 | 0 | 0 | 0 | 865 | 9951 | 43267 | 189148 | 8004 | 3854210 | 15288 | 1410355 | 99153 | 9663 | 1010 | 216 | 433 | 433 | 0 | 0 | 0 | 0 |
| **Red Sea** | 449811 | 0 | 0 | 0 | 0 | 0 | 0 | 0 | 0 | 72 | 72 | 24802 | 1864 | 383791 | 430 | 11326 | 13190 | 7455 | 2366 | 1434 | 932 | 573 | 430 | 502 | 215 | 358 |
| **Rio de La Plata** | 31797 | 0 | 0 | 0 | 0 | 0 | 0 | 0 | 0 | 0 | 0 | 0 | 0 | 31797 | 0 | 0 | 0 | 0 | 0 | 0 | 0 | 0 | 0 | 0 | 0 | 0 |
| **Savu Sea** | 106234 | 0 | 0 | 0 | 0 | 0 | 0 | 0 | 0 | 0 | 0 | 239 | 0 | 97554 | 80 | 4937 | 3345 | 0 | 80 | 0 | 0 | 0 | 0 | 0 | 0 | 0 |
| **Sea of Azov** | 39547 | 0 | 0 | 0 | 0 | 0 | 0 | 0 | 0 | 0 | 0 | 198 | 40 | 38122 | 158 | 435 | 317 | 158 | 40 | 40 | 40 | 0 | 0 | 0 | 0 | 0 |
| **Sea of Marmara** | 11675 | 0 | 0 | 0 | 0 | 0 | 0 | 0 | 96 | 48 | 191 | 0 | 0 | 6890 | 96 | 287 | 96 | 383 | 478 | 718 | 622 | 574 | 383 | 335 | 0 | 478 |
| **Sea of Okhotsk** | 1613208 | 0 | 0 | 0 | 0 | 84 | 1689 | 11822 | 36846 | 64206 | 68682 | 36030 | 3913 | 1247139 | 3800 | 27923 | 34482 | 24123 | 15482 | 10330 | 9345 | 6136 | 4194 | 2477 | 1999 | 2505 |
| **Seto Naikai or Inland Sea** | 18131 | 0 | 0 | 0 | 0 | 0 | 0 | 57 | 0 | 0 | 0 | 57 | 0 | 17961 | 0 | 57 | 0 | 0 | 0 | 0 | 0 | 0 | 0 | 0 | 0 | 0 |
| **Singapore Strait** | 2684 | 0 | 0 | 0 | 0 | 0 | 0 | 0 | 0 | 0 | 0 | 0 | 0 | 2684 | 0 | 0 | 0 | 0 | 0 | 0 | 0 | 0 | 0 | 0 | 0 | 0 |
| **Skagerrak** | 32040 | 0 | 0 | 0 | 0 | 0 | 0 | 23 | 140 | 93 | 1094 | 558 | 47 | 29225 | 23 | 326 | 326 | 116 | 23 | 23 | 0 | 0 | 0 | 0 | 0 | 23 |
| **Solomon Sea** | 744334 | 0 | 0 | 0 | 0 | 0 | 0 | 0 | 0 | 0 | 1845 | 23581 | 401 | 543973 | 1364 | 119109 | 52055 | 2005 | 0 | 0 | 0 | 0 | 0 | 0 | 0 | 0 |
| **South Atlantic Ocean** | 40501812 | 0 | 0 | 0 | 0 | 1532 | 6968 | 69581 | 452274 | 1044204 | 2287562 | 5475571 | 90188 | 27719582 | 20805 | 1490054 | 942254 | 369202 | 177065 | 101999 | 72002 | 41314 | 29750 | 22930 | 16950 | 70025 |
| **South China Sea** | 3362904 | 0 | 0 | 0 | 0 | 0 | 0 | 0 | 0 | 0 | 777 | 90476 | 3029 | 2849250 | 5825 | 311112 | 93971 | 6679 | 932 | 311 | 78 | 78 | 78 | 78 | 155 | 78 |
| **South Pacific Ocean** | 76681173 | 0 | 0 | 105 | 369 | 580 | 2319 | 8275 | 33945 | 880139 | 6622335 | 11943278 | 231604 | 52220665 | 54027 | 3101253 | 1061406 | 356420 | 105998 | 33312 | 9804 | 4691 | 3584 | 2530 | 1318 | 3215 |
| **Southern Ocean** | 21695157 | 964 | 679 | 3810 | 10533 | 30044 | 177241 | 589554 | 1183049 | 1672662 | 1732640 | 615393 | 7839 | 14033716 | 2628 | 115861 | 209365 | 223795 | 197540 | 171722 | 148511 | 115489 | 95321 | 79664 | 62080 | 215058 |
| **Strait of Gibraltar** | 1664 | 0 | 0 | 0 | 0 | 0 | 0 | 0 | 0 | 0 | 0 | 0 | 0 | 1553 | 0 | 111 | 0 | 0 | 0 | 0 | 0 | 0 | 0 | 0 | 0 | 0 |
| **Sulu Sea** | 337029 | 0 | 0 | 0 | 0 | 0 | 0 | 0 | 0 | 0 | 241 | 1366 | 161 | 302546 | 161 | 24194 | 7958 | 402 | 0 | 0 | 0 | 0 | 0 | 0 | 0 | 0 |
| **Tasman Sea** | 3344624 | 0 | 0 | 0 | 0 | 48 | 0 | 95 | 48 | 2670 | 38625 | 75153 | 1240 | 2582464 | 2337 | 334658 | 269233 | 35335 | 2480 | 191 | 0 | 0 | 0 | 48 | 0 | 0 |
| **The Coastal Waters of Southeast Alaska and British Columbia** | 124532 | 0 | 0 | 0 | 0 | 131 | 98 | 524 | 1277 | 1867 | 1703 | 1244 | 65 | 104492 | 98 | 1801 | 3373 | 2587 | 1867 | 851 | 786 | 426 | 393 | 360 | 262 | 327 |
| **The Northwestern Passages** | 1062173 | 0 | 0 | 17 | 17 | 34 | 34 | 1143 | 3952 | 12040 | 22364 | 20683 | 3430 | 914047 | 8744 | 32941 | 27997 | 11703 | 2438 | 404 | 135 | 17 | 17 | 17 | 0 | 0 |
| **Timor Sea** | 434186 | 0 | 0 | 0 | 0 | 0 | 0 | 0 | 0 | 0 | 0 | 0 | 0 | 359811 | 476 | 37624 | 35799 | 476 | 0 | 0 | 0 | 0 | 0 | 0 | 0 | 0 |
| **Tyrrhenian Sea** | 217465 | 0 | 0 | 0 | 0 | 0 | 0 | 0 | 0 | 0 | 0 | 190 | 0 | 176803 | 808 | 36529 | 3088 | 48 | 0 | 0 | 0 | 0 | 0 | 0 | 0 | 0 |
| **White Sea** | 90531 | 0 | 0 | 0 | 0 | 0 | 81 | 162 | 837 | 3602 | 4533 | 2334 | 715 | 73126 | 162 | 526 | 1214 | 837 | 567 | 553 | 351 | 391 | 229 | 94 | 67 | 148 |
| **Yellow Sea** | 408198 | 53 | 0 | 0 | 0 | 0 | 106 | 0 | 0 | 0 | 0 | 0 | 0 | 407879 | 106 | 53 | 0 | 0 | 0 | 0 | 0 | 0 | 0 | 0 | 0 | 0 |

**Table S7| Absolute changes in the depth of the photic zone (*Z_photic_*) across the global ocean and International Hydrographic Office (IHO) regions between 2003 and 2022 when illuminated by moonlight.** *Z_photic_* was calculated using Beers Law (see methods). The annual average of monthly maximum zenith surface lunar irradiance was modelled at 9km resolution. The depth of the photic zone was defined as the minimum irradiance at 520nm that elicits diel vertical migration in Calanus copepods. *K_d_*(490) was taken as the modelled values for 2003 and 2022 given by quantile regression on the median, with serial autocorrelation removed using ARIMA models.

| IHO region | Total Area (km^2^) | Area where *Z_photic_* is decreasing (km^2^) | | | | | | | | | | | | Area no change in *Z_photic_* (km^2^) | Area where *Z_photic_* is increasing (km^2^) | | | | | | | | | | | |
| --- | --- | --- | --- | --- | --- | --- | --- | --- | --- | --- | --- | --- | --- | --- | --- | --- | --- | --- | --- | --- | --- | --- | --- | --- | --- | --- |
|  |  | >100m | 90-100m | 80-90m | 70-80m | 60-70m | 50-60m | 40-50m | 30-40m | 20-30m | 10-20m | 1-10m | 0-1m |  | 0-1m | 1-10m | 10-20m | 20-30m | 30-40m | 40-50m | 50-60m | 60-70m | 70-80m | 80-90m | 90-100m | >100m |
| **Global Ocean** | **359351781** | **45418** | **57503** | **142951** | **296442** | **524856** | **1026396** | **2294271** | **5341359** | **12960588** | **27139996** | **23115095** | **710909** | **250429108** | **282185** | **12602493** | **13357865** | **5645480** | **2003352** | **718527** | **306124** | **160820** | **87327** | **43828** | **20843** | **38044** |
| **Adriatic Sea** | 139454 | 0 | 0 | 0 | 0 | 0 | 0 | 0 | 0 | 220 | 2246 | 8895 | 3743 | 123910 | 132 | 264 | 44 | 0 | 0 | 0 | 0 | 0 | 0 | 0 | 0 | 0 |
| **Aegean Sea** | 191305 | 0 | 0 | 0 | 0 | 0 | 0 | 0 | 101 | 963 | 6792 | 10544 | 659 | 143910 | 1014 | 12520 | 12825 | 1977 | 0 | 0 | 0 | 0 | 0 | 0 | 0 | 0 |
| **Alboran Sea** | 54711 | 0 | 0 | 0 | 0 | 0 | 0 | 0 | 0 | 107 | 801 | 2458 | 53 | 40552 | 267 | 7373 | 2992 | 107 | 0 | 0 | 0 | 0 | 0 | 0 | 0 | 0 |
| **Andaman or Burma Sea** | 613099 | 0 | 0 | 0 | 0 | 0 | 0 | 0 | 0 | 7318 | 54485 | 45100 | 1511 | 494981 | 875 | 4057 | 2545 | 1989 | 239 | 0 | 0 | 0 | 0 | 0 | 0 | 0 |
| **Arabian Sea** | 4241184 | 0 | 0 | 0 | 0 | 154 | 1462 | 9928 | 43097 | 134908 | 225412 | 127828 | 5079 | 3204707 | 4925 | 196244 | 220178 | 58258 | 8312 | 693 | 0 | 0 | 0 | 0 | 0 | 0 |
| **Arafura Sea** | 1025736 | 0 | 0 | 0 | 0 | 0 | 0 | 0 | 0 | 0 | 477 | 875 | 477 | 901992 | 7555 | 48670 | 32367 | 20041 | 11054 | 1909 | 318 | 0 | 0 | 0 | 0 | 0 |
| **Baffin Bay** | 529960 | 0 | 0 | 0 | 0 | 0 | 0 | 0 | 20 | 1634 | 36914 | 95737 | 2240 | 376085 | 1198 | 13861 | 1986 | 286 | 0 | 0 | 0 | 0 | 0 | 0 | 0 | 0 |
| **Balearic (Iberian Sea)** | 80078 | 0 | 0 | 0 | 0 | 0 | 0 | 0 | 0 | 0 | 0 | 332 | 0 | 62932 | 380 | 12349 | 3420 | 665 | 0 | 0 | 0 | 0 | 0 | 0 | 0 | 0 |
| **Bali Sea** | 39916 | 0 | 0 | 0 | 0 | 0 | 0 | 0 | 0 | 0 | 0 | 81 | 0 | 28419 | 81 | 9797 | 1457 | 81 | 0 | 0 | 0 | 0 | 0 | 0 | 0 | 0 |
| **Baltic Sea** | 215863 | 0 | 0 | 0 | 0 | 0 | 0 | 0 | 0 | 0 | 0 | 58612 | 13797 | 141320 | 1414 | 720 | 0 | 0 | 0 | 0 | 0 | 0 | 0 | 0 | 0 | 0 |
| **Banda Sea** | 693842 | 0 | 0 | 0 | 0 | 0 | 0 | 0 | 81 | 162 | 1137 | 1787 | 0 | 440738 | 325 | 38664 | 107951 | 66687 | 28348 | 6011 | 1625 | 325 | 0 | 0 | 0 | 0 |
| **Barentsz Sea** | 1408430 | 989 | 497 | 1436 | 3216 | 6477 | 12333 | 25818 | 41836 | 52287 | 61228 | 67417 | 12649 | 1007951 | 1328 | 52920 | 45804 | 12174 | 1763 | 220 | 40 | 45 | 0 | 0 | 0 | 0 |
| **Bass Strait** | 112699 | 0 | 0 | 0 | 0 | 0 | 0 | 0 | 49 | 196 | 293 | 2054 | 342 | 109617 | 98 | 49 | 0 | 0 | 0 | 0 | 0 | 0 | 0 | 0 | 0 | 0 |
| **Bay of Bengal** | 2207565 | 0 | 0 | 0 | 0 | 0 | 0 | 1556 | 56644 | 344455 | 486221 | 161529 | 1167 | 1129537 | 856 | 12371 | 8559 | 2256 | 1478 | 545 | 311 | 78 | 0 | 0 | 0 | 0 |
| **Bay of Biscay** | 174437 | 0 | 0 | 0 | 0 | 0 | 0 | 0 | 121 | 5295 | 23482 | 21744 | 2021 | 114742 | 40 | 3395 | 2991 | 606 | 0 | 0 | 0 | 0 | 0 | 0 | 0 | 0 |
| **Bay of Fundy** | 16034 | 0 | 0 | 0 | 0 | 0 | 0 | 0 | 0 | 0 | 565 | 2694 | 304 | 10516 | 174 | 1738 | 43 | 0 | 0 | 0 | 0 | 0 | 0 | 0 | 0 | 0 |
| **Beaufort Sea** | 431132 | 0 | 0 | 0 | 0 | 0 | 0 | 63 | 922 | 3372 | 7290 | 21785 | 19271 | 329497 | 4546 | 29452 | 10934 | 2367 | 503 | 880 | 251 | 0 | 0 | 0 | 0 | 0 |
| **Bering Sea** | 2336912 | 0 | 0 | 0 | 0 | 22 | 774 | 7081 | 18941 | 37262 | 108601 | 186312 | 34319 | 1637799 | 12458 | 96232 | 117164 | 54433 | 18985 | 4602 | 686 | 664 | 89 | 288 | 89 | 111 |
| **Bismarck Sea** | 345053 | 0 | 0 | 0 | 0 | 0 | 0 | 0 | 0 | 163 | 2451 | 8005 | 327 | 238776 | 82 | 28101 | 47788 | 17808 | 1552 | 0 | 0 | 0 | 0 | 0 | 0 | 0 |
| **Black Sea** | 423026 | 0 | 0 | 0 | 0 | 0 | 0 | 0 | 0 | 43 | 16264 | 93905 | 908 | 224359 | 7137 | 54846 | 23098 | 2422 | 43 | 0 | 0 | 0 | 0 | 0 | 0 | 0 |
| **Bristol Channel** | 5748 | 0 | 0 | 0 | 0 | 0 | 0 | 0 | 0 | 0 | 0 | 981 | 105 | 4591 | 0 | 70 | 0 | 0 | 0 | 0 | 0 | 0 | 0 | 0 | 0 | 0 |
| **Caribbean Sea** | 2852792 | 0 | 0 | 0 | 76 | 0 | 0 | 0 | 3349 | 51838 | 279288 | 272894 | 5328 | 1994222 | 2131 | 205984 | 33037 | 3730 | 837 | 76 | 0 | 0 | 0 | 0 | 0 | 0 |
| **Celebes Sea** | 457342 | 0 | 0 | 0 | 0 | 0 | 0 | 0 | 0 | 0 | 0 | 82 | 82 | 309659 | 164 | 45997 | 80988 | 18399 | 1889 | 82 | 0 | 0 | 0 | 0 | 0 | 0 |
| **Celtic Sea** | 215080 | 0 | 0 | 0 | 0 | 0 | 0 | 0 | 68 | 1157 | 18990 | 34066 | 272 | 147868 | 238 | 8338 | 3607 | 476 | 0 | 0 | 0 | 0 | 0 | 0 | 0 | 0 |
| **Ceram Sea** | 161489 | 0 | 0 | 0 | 0 | 0 | 0 | 0 | 0 | 0 | 82 | 1072 | 0 | 96992 | 247 | 13691 | 29856 | 16578 | 2969 | 0 | 0 | 0 | 0 | 0 | 0 | 0 |
| **Chukchi Sea** | 346259 | 0 | 0 | 0 | 0 | 0 | 0 | 0 | 0 | 60 | 9688 | 89461 | 44337 | 191634 | 4526 | 6321 | 232 | 0 | 0 | 0 | 0 | 0 | 0 | 0 | 0 | 0 |
| **Coral Sea** | 4125541 | 0 | 0 | 0 | 0 | 0 | 0 | 0 | 147 | 4402 | 48713 | 69034 | 2201 | 3318480 | 807 | 284793 | 313111 | 75197 | 8217 | 440 | 0 | 0 | 0 | 0 | 0 | 0 |
| **Davis Strait** | 749737 | 0 | 0 | 0 | 0 | 30 | 0 | 89 | 354 | 7395 | 29594 | 78259 | 2583 | 610414 | 1830 | 13698 | 4162 | 1063 | 236 | 30 | 0 | 0 | 0 | 0 | 0 | 0 |
| **East Siberian Sea** | 633057 | 0 | 0 | 0 | 0 | 0 | 0 | 0 | 0 | 0 | 3511 | 63505 | 41563 | 465299 | 45210 | 13970 | 0 | 0 | 0 | 0 | 0 | 0 | 0 | 0 | 0 | 0 |
| **Eastern China Sea** | 761356 | 0 | 0 | 0 | 0 | 0 | 0 | 64 | 641 | 13137 | 32169 | 66389 | 10702 | 562574 | 2948 | 51009 | 19481 | 2115 | 128 | 0 | 0 | 0 | 0 | 0 | 0 | 0 |
| **English Channel** | 81419 | 0 | 0 | 0 | 0 | 0 | 0 | 0 | 0 | 0 | 68 | 1388 | 237 | 63747 | 1049 | 12594 | 2268 | 68 | 0 | 0 | 0 | 0 | 0 | 0 | 0 | 0 |
| **Flores Sea** | 102816 | 0 | 0 | 0 | 0 | 0 | 0 | 0 | 0 | 0 | 0 | 162 | 0 | 95466 | 81 | 4442 | 2342 | 242 | 81 | 0 | 0 | 0 | 0 | 0 | 0 | 0 |
| **Great Australian Bight** | 1326209 | 0 | 0 | 0 | 0 | 0 | 0 | 1097 | 21569 | 41362 | 87007 | 54210 | 1932 | 1095895 | 1776 | 16503 | 4805 | 52 | 0 | 0 | 0 | 0 | 0 | 0 | 0 | 0 |
| **Greenland Sea** | 1186456 | 1548 | 541 | 1114 | 1409 | 1802 | 4554 | 13015 | 55074 | 110491 | 137143 | 95338 | 3555 | 711426 | 1327 | 28012 | 15865 | 3596 | 467 | 115 | 66 | 0 | 0 | 0 | 0 | 0 |
| **Gulf of Aden** | 263754 | 0 | 0 | 0 | 0 | 0 | 0 | 0 | 0 | 233 | 1089 | 1789 | 0 | 177806 | 467 | 7622 | 21234 | 22790 | 17034 | 8478 | 3656 | 1011 | 389 | 78 | 78 | 0 |
| **Gulf of Alaska** | 415503 | 0 | 0 | 0 | 0 | 0 | 0 | 46 | 185 | 2838 | 17606 | 36227 | 2746 | 319213 | 669 | 20375 | 11768 | 3184 | 554 | 92 | 0 | 0 | 0 | 0 | 0 | 0 |
| **Gulf of Aqaba** | 3555 | 0 | 0 | 0 | 0 | 0 | 0 | 0 | 0 | 0 | 437 | 187 | 0 | 2931 | 0 | 0 | 0 | 0 | 0 | 0 | 0 | 0 | 0 | 0 | 0 | 0 |
| **Gulf of Boni** | 33237 | 0 | 0 | 0 | 0 | 0 | 0 | 0 | 0 | 0 | 1140 | 4155 | 81 | 27616 | 81 | 163 | 0 | 0 | 0 | 0 | 0 | 0 | 0 | 0 | 0 | 0 |
| **Gulf of Bothnia** | 113419 | 0 | 0 | 0 | 0 | 0 | 0 | 0 | 0 | 0 | 0 | 47354 | 12929 | 52373 | 763 | 0 | 0 | 0 | 0 | 0 | 0 | 0 | 0 | 0 | 0 | 0 |
| **Gulf of California** | 180703 | 0 | 0 | 0 | 0 | 0 | 0 | 0 | 0 | 0 | 260 | 5590 | 65 | 145082 | 1040 | 20540 | 7670 | 390 | 65 | 0 | 0 | 0 | 0 | 0 | 0 | 0 |
| **Gulf of Finland** | 57077 | 187 | 47 | 47 | 0 | 47 | 140 | 0 | 0 | 0 | 0 | 513 | 3150 | 51687 | 1260 | 0 | 0 | 0 | 0 | 0 | 0 | 0 | 0 | 0 | 0 | 0 |
| **Gulf of Guinea** | 754844 | 330 | 0 | 165 | 577 | 825 | 2226 | 2639 | 5113 | 10143 | 24903 | 24326 | 907 | 558505 | 495 | 25975 | 44116 | 27047 | 13359 | 5442 | 1979 | 1814 | 1319 | 742 | 742 | 1154 |
| **Gulf of Mexico** | 1566759 | 0 | 0 | 0 | 0 | 403 | 940 | 873 | 6113 | 24048 | 54545 | 51052 | 7658 | 1293967 | 1948 | 68047 | 50850 | 5441 | 873 | 0 | 0 | 0 | 0 | 0 | 0 | 0 |
| **Gulf of Oman** | 111892 | 0 | 0 | 0 | 0 | 0 | 0 | 0 | 0 | 0 | 0 | 611 | 68 | 74278 | 272 | 9098 | 20165 | 5703 | 1426 | 272 | 0 | 0 | 0 | 0 | 0 | 0 |
| **Gulf of Riga** | 18692 | 0 | 0 | 0 | 0 | 0 | 0 | 0 | 0 | 0 | 0 | 459 | 2021 | 15776 | 436 | 0 | 0 | 0 | 0 | 0 | 0 | 0 | 0 | 0 | 0 | 0 |
| **Gulf of St Lawrence** | 290874 | 0 | 0 | 0 | 0 | 0 | 0 | 0 | 0 | 109 | 10598 | 54081 | 5481 | 202966 | 2069 | 13502 | 1416 | 581 | 73 | 0 | 0 | 0 | 0 | 0 | 0 | 0 |
| **Gulf of Suez** | 10438 | 0 | 0 | 0 | 0 | 0 | 0 | 0 | 0 | 0 | 0 | 0 | 0 | 10438 | 0 | 0 | 0 | 0 | 0 | 0 | 0 | 0 | 0 | 0 | 0 | 0 |
| **Gulf of Thailand** | 297927 | 0 | 0 | 0 | 0 | 0 | 0 | 0 | 0 | 0 | 0 | 560 | 160 | 296087 | 400 | 720 | 0 | 0 | 0 | 0 | 0 | 0 | 0 | 0 | 0 | 0 |
| **Gulf of Tomini** | 56739 | 0 | 0 | 0 | 0 | 0 | 0 | 0 | 0 | 0 | 164 | 0 | 0 | 40786 | 0 | 3289 | 9703 | 2302 | 493 | 0 | 0 | 0 | 0 | 0 | 0 | 0 |
| **Halmahera Sea** | 75295 | 0 | 0 | 0 | 0 | 0 | 0 | 0 | 0 | 326 | 0 | 245 | 0 | 27736 | 326 | 7994 | 31733 | 6934 | 0 | 0 | 0 | 0 | 0 | 0 | 0 | 0 |
| **Hudson Bay** | 832649 | 0 | 0 | 0 | 0 | 0 | 0 | 0 | 127 | 3979 | 84613 | 143173 | 24148 | 563445 | 2286 | 9503 | 1376 | 0 | 0 | 0 | 0 | 0 | 0 | 0 | 0 | 0 |
| **Hudson Strait** | 200039 | 0 | 0 | 0 | 0 | 0 | 0 | 0 | 36 | 1091 | 15423 | 48250 | 2583 | 128910 | 1128 | 2128 | 491 | 0 | 0 | 0 | 0 | 0 | 0 | 0 | 0 | 0 |
| **Indian Ocean** | 58230954 | 0 | 1740 | 13619 | 58901 | 117952 | 203794 | 332185 | 771734 | 2392494 | 5665130 | 3749316 | 30768 | 40570698 | 6810 | 1158495 | 1715201 | 886057 | 344710 | 130130 | 49060 | 23362 | 7903 | 795 | 0 | 99 |
| **Inner Seas off the West Coast of Scotland** | 44260 | 0 | 0 | 0 | 0 | 0 | 0 | 0 | 0 | 0 | 1342 | 8671 | 594 | 28517 | 310 | 3045 | 1600 | 155 | 26 | 0 | 0 | 0 | 0 | 0 | 0 | 0 |
| **Ionian Sea** | 171778 | 0 | 0 | 0 | 0 | 0 | 0 | 0 | 0 | 0 | 1011 | 6167 | 506 | 152922 | 51 | 8038 | 2983 | 101 | 0 | 0 | 0 | 0 | 0 | 0 | 0 | 0 |
| **Irish Sea and St Georges Channel** | 45922 | 0 | 0 | 0 | 0 | 0 | 0 | 0 | 0 | 0 | 0 | 3664 | 320 | 35074 | 1018 | 5613 | 233 | 0 | 0 | 0 | 0 | 0 | 0 | 0 | 0 | 0 |
| **Japan Sea** | 1066307 | 0 | 0 | 0 | 93 | 278 | 3341 | 17448 | 70487 | 156566 | 120788 | 59397 | 3063 | 592016 | 1717 | 32854 | 7610 | 510 | 139 | 0 | 0 | 0 | 0 | 0 | 0 | 0 |
| **Java Sea** | 566693 | 0 | 0 | 0 | 0 | 0 | 0 | 0 | 0 | 0 | 0 | 898 | 327 | 543666 | 327 | 15106 | 5716 | 653 | 0 | 0 | 0 | 0 | 0 | 0 | 0 | 0 |
| **Kara Sea** | 896938 | 0 | 0 | 0 | 0 | 0 | 0 | 57 | 172 | 1019 | 13242 | 69324 | 27537 | 662197 | 7588 | 48665 | 32138 | 21918 | 9122 | 3216 | 641 | 103 | 0 | 0 | 0 | 0 |
| **Kattegat** | 35447 | 0 | 0 | 0 | 0 | 0 | 0 | 0 | 0 | 0 | 51 | 5793 | 4517 | 25035 | 26 | 26 | 0 | 0 | 0 | 0 | 0 | 0 | 0 | 0 | 0 | 0 |
| **Labrador Sea** | 866470 | 0 | 0 | 0 | 0 | 0 | 0 | 51 | 0 | 7274 | 55111 | 85375 | 2594 | 686233 | 585 | 12436 | 10453 | 5570 | 738 | 51 | 0 | 0 | 0 | 0 | 0 | 0 |
| **Laccadive Sea** | 846351 | 0 | 0 | 81 | 81 | 968 | 1371 | 2580 | 9999 | 29351 | 56606 | 29190 | 726 | 664760 | 403 | 14514 | 27819 | 6290 | 1290 | 323 | 0 | 0 | 0 | 0 | 0 | 0 |
| **Laptev Sea** | 513667 | 0 | 0 | 0 | 0 | 0 | 0 | 55 | 498 | 2647 | 18305 | 146373 | 28349 | 304262 | 3090 | 9977 | 111 | 0 | 0 | 0 | 0 | 0 | 0 | 0 | 0 | 0 |
| **Ligurian Sea** | 16949 | 0 | 0 | 0 | 0 | 0 | 0 | 0 | 0 | 0 | 0 | 85 | 0 | 8008 | 42 | 3941 | 4788 | 85 | 0 | 0 | 0 | 0 | 0 | 0 | 0 | 0 |
| **Makassar Strait** | 198117 | 0 | 0 | 0 | 0 | 0 | 0 | 0 | 0 | 0 | 82 | 988 | 0 | 132984 | 412 | 27008 | 32031 | 4611 | 0 | 0 | 0 | 0 | 0 | 0 | 0 | 0 |
| **Malacca Strait** | 195534 | 0 | 0 | 0 | 0 | 0 | 0 | 0 | 0 | 0 | 0 | 164 | 0 | 159087 | 2134 | 16253 | 13791 | 3940 | 164 | 0 | 0 | 0 | 0 | 0 | 0 | 0 |
| **Mediterranean Sea - Eastern Basin** | 1173640 | 0 | 0 | 0 | 0 | 0 | 56 | 112 | 335 | 2236 | 24711 | 35948 | 1342 | 1073903 | 224 | 20126 | 13753 | 839 | 56 | 0 | 0 | 0 | 0 | 0 | 0 | 0 |
| **Mediterranean Sea - Western Basin** | 476707 | 0 | 0 | 0 | 0 | 0 | 0 | 0 | 0 | 0 | 3888 | 10887 | 194 | 429075 | 1312 | 19442 | 10644 | 1118 | 146 | 0 | 0 | 0 | 0 | 0 | 0 | 0 |
| **Molukka Sea** | 221671 | 0 | 0 | 0 | 0 | 0 | 0 | 0 | 0 | 0 | 0 | 164 | 0 | 144740 | 247 | 6658 | 28027 | 31562 | 9945 | 329 | 0 | 0 | 0 | 0 | 0 | 0 |
| **Mozambique Channel** | 1394283 | 0 | 0 | 0 | 0 | 0 | 0 | 0 | 0 | 0 | 435 | 11167 | 0 | 995171 | 870 | 106232 | 232622 | 44015 | 3553 | 218 | 0 | 0 | 0 | 0 | 0 | 0 |
| **North Atlantic Ocean** | 34507128 | 103 | 51 | 463 | 1799 | 5756 | 16138 | 65426 | 454281 | 1775700 | 3728251 | 2903823 | 42144 | 21669867 | 20558 | 1334679 | 1398666 | 704318 | 257489 | 77041 | 25543 | 13928 | 6116 | 2159 | 1131 | 1696 |
| **North Pacific Ocean** | 65154681 | 0 | 0 | 124 | 559 | 3415 | 15956 | 53270 | 184460 | 617205 | 2459444 | 4350111 | 56561 | 48503129 | 25331 | 3142274 | 3434082 | 1633131 | 545681 | 108838 | 18254 | 2608 | 248 | 0 | 0 | 0 |
| **North Sea** | 524493 | 0 | 0 | 0 | 0 | 0 | 0 | 0 | 0 | 74 | 7207 | 36898 | 12069 | 401385 | 5479 | 47116 | 12957 | 1234 | 74 | 0 | 0 | 0 | 0 | 0 | 0 | 0 |
| **Norwegian Sea** | 1437096 | 0 | 0 | 0 | 0 | 10 | 10 | 0 | 220 | 9888 | 121946 | 149069 | 1658 | 1059994 | 913 | 52702 | 36254 | 3768 | 630 | 31 | 0 | 0 | 0 | 0 | 0 | 0 |
| **Persian Gulf** | 244657 | 0 | 0 | 0 | 0 | 0 | 0 | 0 | 0 | 0 | 0 | 329 | 132 | 229382 | 2107 | 4740 | 5991 | 1843 | 132 | 0 | 0 | 0 | 0 | 0 | 0 | 0 |
| **Philippine Sea** | 5641996 | 0 | 0 | 0 | 72 | 721 | 1442 | 3533 | 14062 | 35118 | 91221 | 106364 | 3966 | 3824068 | 1875 | 556988 | 845867 | 140834 | 14567 | 1010 | 216 | 0 | 72 | 0 | 0 | 0 |
| **Red Sea** | 449811 | 0 | 0 | 0 | 0 | 0 | 0 | 0 | 0 | 143 | 8602 | 23369 | 215 | 352680 | 2366 | 18638 | 26594 | 13548 | 2724 | 932 | 0 | 0 | 0 | 0 | 0 | 0 |
| **Rio de La Plata** | 31797 | 0 | 0 | 0 | 0 | 0 | 0 | 0 | 0 | 0 | 0 | 573 | 446 | 30586 | 191 | 0 | 0 | 0 | 0 | 0 | 0 | 0 | 0 | 0 | 0 | 0 |
| **Savu Sea** | 106234 | 0 | 0 | 0 | 0 | 0 | 0 | 0 | 0 | 0 | 80 | 398 | 0 | 96757 | 80 | 4460 | 4380 | 80 | 0 | 0 | 0 | 0 | 0 | 0 | 0 | 0 |
| **Sea of Azov** | 39547 | 0 | 0 | 0 | 0 | 0 | 0 | 0 | 0 | 0 | 0 | 713 | 1188 | 7403 | 5027 | 25217 | 0 | 0 | 0 | 0 | 0 | 0 | 0 | 0 | 0 | 0 |
| **Sea of Marmara** | 11675 | 0 | 0 | 0 | 0 | 0 | 0 | 0 | 0 | 0 | 0 | 287 | 144 | 6316 | 48 | 2297 | 2392 | 191 | 0 | 0 | 0 | 0 | 0 | 0 | 0 | 0 |
| **Sea of Okhotsk** | 1613208 | 0 | 0 | 0 | 0 | 0 | 0 | 760 | 7403 | 42729 | 103079 | 114761 | 6727 | 1172434 | 2984 | 79857 | 57507 | 16636 | 6052 | 1661 | 619 | 0 | 0 | 0 | 0 | 0 |
| **Seto Naikai or Inland Sea** | 18131 | 0 | 0 | 0 | 0 | 0 | 0 | 0 | 0 | 0 | 57 | 963 | 340 | 14108 | 397 | 1870 | 397 | 0 | 0 | 0 | 0 | 0 | 0 | 0 | 0 | 0 |
| **Singapore Strait** | 2684 | 0 | 0 | 0 | 0 | 0 | 0 | 0 | 0 | 0 | 0 | 0 | 0 | 1944 | 93 | 648 | 0 | 0 | 0 | 0 | 0 | 0 | 0 | 0 | 0 | 0 |
| **Skagerrak** | 32040 | 0 | 0 | 0 | 0 | 0 | 0 | 0 | 0 | 0 | 0 | 1466 | 652 | 26828 | 628 | 2466 | 0 | 0 | 0 | 0 | 0 | 0 | 0 | 0 | 0 | 0 |
| **Solomon Sea** | 744334 | 0 | 0 | 0 | 0 | 0 | 0 | 0 | 0 | 1765 | 11791 | 12833 | 160 | 532103 | 401 | 67535 | 83738 | 30239 | 3770 | 0 | 0 | 0 | 0 | 0 | 0 | 0 |
| **South Atlantic Ocean** | 40501812 | 99 | 890 | 2421 | 11465 | 46206 | 143510 | 343506 | 704802 | 1451165 | 3787953 | 3095308 | 40770 | 27449727 | 9686 | 1259274 | 1244844 | 519583 | 191693 | 84703 | 45959 | 24956 | 13887 | 9241 | 4843 | 15320 |
| **South China Sea** | 3362904 | 0 | 0 | 0 | 0 | 0 | 0 | 0 | 78 | 1165 | 40695 | 63217 | 3106 | 2779354 | 5048 | 195785 | 208832 | 57780 | 6679 | 932 | 155 | 0 | 0 | 0 | 78 | 0 |
| **South Pacific Ocean** | 76681173 | 0 | 0 | 0 | 1318 | 28885 | 180740 | 766814 | 1878768 | 4183689 | 7500259 | 5170617 | 101676 | 52057530 | 13335 | 2093558 | 1691070 | 655228 | 230076 | 86970 | 30044 | 8539 | 2056 | 0 | 0 | 0 |
| **Southern Ocean** | 21695157 | 42110 | 53737 | 123482 | 216876 | 310906 | 437607 | 646205 | 989038 | 1380462 | 1342141 | 494147 | 3000 | 14009561 | 1818 | 196008 | 366986 | 316555 | 243548 | 191562 | 126701 | 83387 | 55248 | 30526 | 13883 | 19664 |
| **Strait of Gibraltar** | 1664 | 0 | 0 | 0 | 0 | 0 | 0 | 0 | 0 | 0 | 0 | 0 | 0 | 1331 | 0 | 277 | 55 | 0 | 0 | 0 | 0 | 0 | 0 | 0 | 0 | 0 |
| **Sulu Sea** | 337029 | 0 | 0 | 0 | 0 | 0 | 0 | 0 | 0 | 0 | 322 | 1527 | 0 | 293946 | 402 | 15835 | 20899 | 3939 | 161 | 0 | 0 | 0 | 0 | 0 | 0 | 0 |
| **Tasman Sea** | 3344624 | 0 | 0 | 0 | 0 | 0 | 0 | 0 | 334 | 6581 | 52073 | 79158 | 1717 | 2539738 | 2432 | 272667 | 300658 | 80875 | 7964 | 429 | 0 | 0 | 0 | 0 | 0 | 0 |
| **The Coastal Waters of Southeast Alaska and British Columbia** | 124532 | 0 | 0 | 0 | 0 | 0 | 0 | 0 | 0 | 0 | 295 | 5960 | 1867 | 101544 | 2259 | 10937 | 1637 | 33 | 0 | 0 | 0 | 0 | 0 | 0 | 0 | 0 |
| **The Northwestern Passages** | 1062173 | 0 | 0 | 0 | 0 | 0 | 0 | 0 | 101 | 2321 | 25845 | 73819 | 44056 | 752923 | 17707 | 104675 | 35615 | 3918 | 942 | 252 | 0 | 0 | 0 | 0 | 0 | 0 |
| **Timor Sea** | 434186 | 0 | 0 | 0 | 0 | 0 | 0 | 0 | 0 | 0 | 318 | 4445 | 794 | 325679 | 1508 | 18018 | 67787 | 15399 | 238 | 0 | 0 | 0 | 0 | 0 | 0 | 0 |
| **Tyrrhenian Sea** | 217465 | 0 | 0 | 0 | 0 | 0 | 0 | 0 | 0 | 0 | 0 | 285 | 48 | 171293 | 428 | 29831 | 14916 | 665 | 0 | 0 | 0 | 0 | 0 | 0 | 0 | 0 |
| **White Sea** | 90531 | 0 | 0 | 0 | 0 | 0 | 0 | 0 | 0 | 0 | 121 | 17189 | 7825 | 57138 | 2078 | 5545 | 445 | 108 | 67 | 13 | 0 | 0 | 0 | 0 | 0 | 0 |
| **Yellow Sea** | 408198 | 53 | 0 | 0 | 0 | 0 | 0 | 0 | 0 | 0 | 5901 | 20787 | 5051 | 331800 | 8985 | 32165 | 3349 | 106 | 0 | 0 | 0 | 0 | 0 | 0 | 0 | 0 |

**Table S8| Relative % changes in the depth of the photic zone (*Z_photic_*) across the global ocean and International Hydrographic Office (IHO) regions between 2003 and 2022 when illuminated by moonlight.** *Z_photic_* was calculated using Beers Law (see methods). The annual average of monthly maximum zenith surface lunar irradiance was modelled at 9km resolution. The depth of the photic zone was defined as the minimum irradiance at 520nm that elicits diel vertical migration in Calanus copepods. *K_d_*(490) was taken as the modelled values for 2003 and 2022 given by quantile regression on the median, with serial autocorrelation removed using ARIMA models.

| IHO region | Total Area (km^2^) | Area where *Z_photic_* is decreasing (km^2^) | | | | | | | | | | | | Area no change in *Z_photic_* (km^2^) | | Area where *Z_photic_* is increasing (km^2^) | | | | | | | | | | | |
| --- | --- | --- | --- | --- | --- | --- | --- | --- | --- | --- | --- | --- | --- | --- | --- | --- | --- | --- | --- | --- | --- | --- | --- | --- | --- | --- | --- |
|  |  | 100% | 90-100% | 80-90% | 70-80% | 60-70% | 50-60% | 40-50% | 30-40% | 20-30% | 10-20% | 1-10% | 0-1% | |  | 0-1% | 1-10% | 10-20% | 20-30% | 30-40% | 40-50% | 50-60% | 60-70% | 70-80% | 80-90% | 90-100% | 100% |
| **Global Ocean** | **359351781** | **1483** | **22** | **740** | **9617** | **57719** | **204008** | **936552** | **2955418** | **7580808** | **22328297** | **38688711** | **897762** | | **250429041** | **353748** | **19069911** | **9400103** | **2641555** | **1175996** | **771532** | **535396** | **357984** | **245541** | **174580** | **121101** | **414156** |
| **Adriatic Sea** | 139454 | 0 | 0 | 0 | 0 | 0 | 0 | 0 | 352 | 484 | 1673 | 9203 | 3391 | | 123910 | 88 | 308 | 44 | 0 | 0 | 0 | 0 | 0 | 0 | 0 | 0 | 0 |
| **Aegean Sea** | 191305 | 0 | 0 | 0 | 0 | 51 | 0 | 101 | 0 | 253 | 1977 | 15866 | 811 | | 143910 | 1115 | 18198 | 7604 | 1369 | 0 | 51 | 0 | 0 | 0 | 0 | 0 | 0 |
| **Alboran Sea** | 54711 | 0 | 0 | 0 | 0 | 0 | 0 | 0 | 53 | 107 | 748 | 2458 | 53 | | 40552 | 374 | 6251 | 3847 | 160 | 53 | 53 | 0 | 0 | 0 | 0 | 0 | 0 |
| **Andaman or Burma Sea** | 613099 | 0 | 0 | 0 | 0 | 0 | 0 | 0 | 0 | 0 | 16067 | 91233 | 1114 | | 494981 | 477 | 5409 | 2148 | 1511 | 0 | 80 | 0 | 80 | 0 | 0 | 0 | 0 |
| **Arabian Sea** | 4241184 | 0 | 0 | 0 | 0 | 0 | 462 | 7773 | 27705 | 88810 | 226181 | 191396 | 5541 | | 3204707 | 4694 | 251116 | 113899 | 39480 | 24319 | 17854 | 10082 | 8081 | 4694 | 3001 | 2309 | 9081 |
| **Arafura Sea** | 1025736 | 0 | 0 | 0 | 0 | 0 | 0 | 0 | 80 | 80 | 239 | 1272 | 159 | | 901992 | 5328 | 37457 | 31572 | 23858 | 15667 | 5646 | 1511 | 477 | 398 | 0 | 0 | 0 |
| **Baffin Bay** | 529960 | 0 | 0 | 0 | 0 | 0 | 0 | 20 | 1504 | 14095 | 59825 | 60548 | 755 | | 376085 | 449 | 9011 | 4356 | 1413 | 1074 | 332 | 306 | 130 | 7 | 7 | 20 | 26 |
| **Balearic (Iberian Sea)** | 80078 | 0 | 0 | 0 | 0 | 0 | 0 | 0 | 0 | 0 | 0 | 332 | 0 | | 62932 | 332 | 13631 | 2470 | 380 | 0 | 0 | 0 | 0 | 0 | 0 | 0 | 0 |
| **Bali Sea** | 39916 | 0 | 0 | 0 | 0 | 0 | 0 | 0 | 0 | 0 | 0 | 81 | 0 | | 28419 | 81 | 10364 | 405 | 162 | 162 | 243 | 0 | 0 | 0 | 0 | 0 | 0 |
| **Baltic Sea** | 215863 | 0 | 0 | 0 | 0 | 0 | 0 | 596 | 8511 | 26849 | 27371 | 8983 | 99 | | 141320 | 50 | 1241 | 670 | 124 | 25 | 0 | 0 | 0 | 0 | 25 | 0 | 0 |
| **Banda Sea** | 693842 | 0 | 0 | 0 | 0 | 0 | 0 | 0 | 0 | 162 | 1056 | 1949 | 0 | | 440738 | 406 | 79359 | 110794 | 48005 | 9179 | 1543 | 81 | 81 | 0 | 244 | 0 | 244 |
| **Barentsz Sea** | 1408430 | 0 | 0 | 0 | 0 | 328 | 18505 | 50077 | 57961 | 55904 | 56124 | 45290 | 1854 | | 1007951 | 243 | 16815 | 29905 | 21794 | 13384 | 9185 | 6641 | 5036 | 3725 | 2159 | 1922 | 3629 |
| **Bass Strait** | 112699 | 0 | 0 | 0 | 0 | 0 | 98 | 49 | 293 | 196 | 734 | 1321 | 245 | | 109617 | 49 | 98 | 0 | 0 | 0 | 0 | 0 | 0 | 0 | 0 | 0 | 0 |
| **Bay of Bengal** | 2207565 | 0 | 0 | 0 | 0 | 0 | 78 | 0 | 545 | 12683 | 489412 | 547145 | 1712 | | 1129537 | 545 | 14083 | 6380 | 2179 | 778 | 700 | 934 | 233 | 78 | 233 | 311 | 0 |
| **Bay of Biscay** | 174437 | 0 | 0 | 0 | 0 | 0 | 0 | 121 | 121 | 8851 | 26554 | 16167 | 849 | | 114742 | 40 | 2546 | 3274 | 768 | 364 | 0 | 0 | 0 | 40 | 0 | 0 | 0 |
| **Bay of Fundy** | 16034 | 0 | 0 | 0 | 0 | 0 | 0 | 43 | 43 | 695 | 1304 | 1390 | 87 | | 10516 | 130 | 391 | 565 | 348 | 174 | 217 | 0 | 0 | 43 | 87 | 0 | 0 |
| **Beaufort Sea** | 431132 | 0 | 0 | 0 | 0 | 0 | 0 | 168 | 2283 | 5781 | 12799 | 25388 | 6850 | | 329497 | 1278 | 6913 | 8902 | 9887 | 7331 | 4818 | 3289 | 1655 | 901 | 566 | 440 | 2388 |
| **Bering Sea** | 2336912 | 0 | 22 | 22 | 22 | 4957 | 17945 | 29828 | 42529 | 65165 | 101033 | 127962 | 4005 | | 1637799 | 1660 | 32018 | 56535 | 58306 | 43923 | 31509 | 21574 | 13741 | 10909 | 8497 | 6395 | 20556 |
| **Bismarck Sea** | 345053 | 0 | 0 | 0 | 0 | 0 | 0 | 0 | 0 | 0 | 817 | 9476 | 654 | | 238776 | 245 | 58979 | 34799 | 1307 | 0 | 0 | 0 | 0 | 0 | 0 | 0 | 0 |
| **Black Sea** | 423026 | 0 | 0 | 0 | 0 | 43 | 130 | 43 | 43 | 260 | 37934 | 72321 | 346 | | 224359 | 3244 | 29456 | 26212 | 9473 | 6056 | 5104 | 2898 | 1946 | 1211 | 952 | 389 | 606 |
| **Bristol Channel** | 5748 | 0 | 0 | 0 | 0 | 0 | 0 | 105 | 386 | 315 | 105 | 175 | 0 | | 4591 | 0 | 0 | 0 | 35 | 35 | 0 | 0 | 0 | 0 | 0 | 0 | 0 |
| **Caribbean Sea** | 2852792 | 0 | 0 | 0 | 0 | 228 | 1751 | 3502 | 5024 | 12408 | 98805 | 480172 | 10885 | | 1994222 | 14082 | 220980 | 8449 | 1370 | 533 | 76 | 76 | 0 | 76 | 0 | 0 | 152 |
| **Celebes Sea** | 457342 | 0 | 0 | 0 | 0 | 0 | 0 | 0 | 0 | 0 | 82 | 0 | 82 | | 309659 | 411 | 104725 | 39590 | 2300 | 411 | 0 | 0 | 0 | 0 | 0 | 0 | 82 |
| **Celtic Sea** | 215080 | 0 | 0 | 0 | 0 | 0 | 0 | 0 | 306 | 4594 | 29914 | 19534 | 204 | | 147868 | 136 | 5275 | 4764 | 1531 | 715 | 238 | 0 | 0 | 0 | 0 | 0 | 0 |
| **Ceram Sea** | 161489 | 0 | 0 | 0 | 0 | 0 | 0 | 0 | 0 | 0 | 247 | 907 | 0 | | 96992 | 82 | 19135 | 28372 | 14268 | 1402 | 82 | 0 | 0 | 0 | 0 | 0 | 0 |
| **Chukchi Sea** | 346259 | 0 | 0 | 0 | 0 | 0 | 0 | 353 | 3649 | 15515 | 30314 | 87908 | 8025 | | 191634 | 736 | 4849 | 1754 | 897 | 161 | 323 | 81 | 0 | 30 | 0 | 30 | 0 |
| **Coral Sea** | 4125541 | 0 | 0 | 0 | 0 | 0 | 0 | 73 | 220 | 147 | 2861 | 114666 | 6529 | | 3318480 | 8510 | 601279 | 72042 | 734 | 0 | 0 | 0 | 0 | 0 | 0 | 0 | 0 |
| **Davis Strait** | 749737 | 0 | 0 | 0 | 30 | 89 | 546 | 4930 | 11985 | 14155 | 32989 | 52901 | 679 | | 610414 | 620 | 9978 | 3336 | 2066 | 1668 | 1314 | 753 | 443 | 354 | 192 | 89 | 207 |
| **East Siberian Sea** | 633057 | 0 | 0 | 0 | 0 | 0 | 0 | 1537 | 5847 | 10805 | 22997 | 55985 | 11408 | | 465299 | 9735 | 33787 | 7354 | 3918 | 1869 | 1281 | 377 | 286 | 121 | 136 | 90 | 226 |
| **Eastern China Sea** | 761356 | 0 | 0 | 0 | 0 | 0 | 0 | 320 | 6857 | 18391 | 29542 | 57994 | 9997 | | 562574 | 2307 | 45242 | 14226 | 5831 | 2820 | 2179 | 1282 | 769 | 449 | 449 | 128 | 0 |
| **English Channel** | 81419 | 0 | 0 | 0 | 0 | 0 | 0 | 0 | 68 | 609 | 576 | 305 | 135 | | 63747 | 203 | 5586 | 5044 | 1219 | 609 | 372 | 576 | 339 | 440 | 237 | 271 | 1083 |
| **Flores Sea** | 102816 | 0 | 0 | 0 | 0 | 0 | 0 | 0 | 0 | 0 | 0 | 162 | 0 | | 95466 | 162 | 6058 | 888 | 0 | 0 | 81 | 0 | 0 | 0 | 0 | 0 | 0 |
| **Great Australian Bight** | 1326209 | 0 | 0 | 0 | 0 | 0 | 0 | 52 | 0 | 10706 | 95729 | 98027 | 2663 | | 1095895 | 1567 | 16555 | 4491 | 209 | 104 | 209 | 0 | 0 | 0 | 0 | 0 | 0 |
| **Greenland Sea** | 1186456 | 0 | 0 | 0 | 147 | 4931 | 12409 | 60324 | 97075 | 110155 | 94315 | 44983 | 1499 | | 711426 | 278 | 14137 | 11246 | 8068 | 6389 | 3891 | 2621 | 1089 | 680 | 213 | 180 | 401 |
| **Gulf of Aden** | 263754 | 0 | 0 | 0 | 0 | 0 | 0 | 0 | 0 | 311 | 1089 | 1711 | 0 | | 177806 | 389 | 3656 | 14312 | 16801 | 15634 | 10189 | 8556 | 3967 | 3111 | 1945 | 933 | 3345 |
| **Gulf of Alaska** | 415503 | 0 | 0 | 23 | 162 | 300 | 669 | 2238 | 5769 | 12668 | 23167 | 14191 | 461 | | 319213 | 46 | 5492 | 12576 | 7961 | 3830 | 2538 | 1569 | 1315 | 485 | 300 | 115 | 415 |
| **Gulf of Aqaba** | 3555 | 0 | 0 | 0 | 0 | 0 | 0 | 0 | 0 | 0 | 0 | 624 | 0 | | 2931 | 0 | 0 | 0 | 0 | 0 | 0 | 0 | 0 | 0 | 0 | 0 | 0 |
| **Gulf of Boni** | 33237 | 0 | 0 | 0 | 0 | 0 | 0 | 0 | 0 | 0 | 0 | 5295 | 81 | | 27616 | 81 | 163 | 0 | 0 | 0 | 0 | 0 | 0 | 0 | 0 | 0 | 0 |
| **Gulf of Bothnia** | 113419 | 0 | 0 | 0 | 0 | 18 | 0 | 1029 | 9400 | 26586 | 19314 | 3884 | 71 | | 52373 | 35 | 195 | 248 | 177 | 89 | 0 | 0 | 0 | 0 | 0 | 0 | 0 |
| **Gulf of California** | 180703 | 0 | 0 | 0 | 0 | 0 | 0 | 0 | 130 | 325 | 910 | 4485 | 65 | | 145082 | 520 | 11115 | 10530 | 5655 | 1235 | 260 | 130 | 130 | 65 | 0 | 0 | 65 |
| **Gulf of Finland** | 57077 | 467 | 0 | 0 | 0 | 0 | 0 | 0 | 537 | 1610 | 957 | 513 | 47 | | 51687 | 0 | 443 | 513 | 210 | 47 | 23 | 0 | 23 | 0 | 0 | 0 | 0 |
| **Gulf of Guinea** | 754844 | 0 | 0 | 330 | 1072 | 2309 | 2721 | 6020 | 6102 | 8576 | 13688 | 30675 | 660 | | 558505 | 412 | 39416 | 37355 | 17399 | 7669 | 4700 | 3134 | 2309 | 2144 | 1484 | 660 | 7504 |
| **Gulf of Mexico** | 1566759 | 0 | 0 | 0 | 0 | 0 | 605 | 3023 | 4971 | 8262 | 38625 | 85512 | 4635 | | 1293967 | 4165 | 108418 | 13166 | 672 | 269 | 269 | 67 | 0 | 67 | 0 | 0 | 67 |
| **Gulf of Oman** | 111892 | 0 | 0 | 0 | 0 | 0 | 0 | 0 | 68 | 272 | 204 | 68 | 68 | | 74278 | 0 | 747 | 2444 | 4685 | 6518 | 6586 | 5771 | 3666 | 2173 | 1290 | 747 | 2308 |
| **Gulf of Riga** | 18692 | 0 | 0 | 0 | 0 | 0 | 0 | 0 | 92 | 505 | 1332 | 505 | 46 | | 15776 | 0 | 161 | 207 | 46 | 23 | 0 | 0 | 0 | 0 | 0 | 0 | 0 |
| **Gulf of St Lawrence** | 290874 | 0 | 0 | 0 | 0 | 36 | 73 | 399 | 3738 | 12740 | 32739 | 19854 | 690 | | 202966 | 218 | 7295 | 5444 | 2033 | 762 | 835 | 145 | 145 | 181 | 254 | 73 | 254 |
| **Gulf of Suez** | 10438 | 0 | 0 | 0 | 0 | 0 | 0 | 0 | 0 | 0 | 0 | 0 | 0 | | 10438 | 0 | 0 | 0 | 0 | 0 | 0 | 0 | 0 | 0 | 0 | 0 | 0 |
| **Gulf of Thailand** | 297927 | 0 | 0 | 0 | 0 | 0 | 80 | 80 | 80 | 160 | 160 | 80 | 80 | | 296087 | 80 | 160 | 400 | 240 | 80 | 80 | 0 | 0 | 0 | 0 | 80 | 0 |
| **Gulf of Tomini** | 56739 | 0 | 0 | 0 | 0 | 0 | 0 | 0 | 0 | 0 | 0 | 164 | 0 | | 40786 | 0 | 10690 | 4358 | 658 | 82 | 0 | 0 | 0 | 0 | 0 | 0 | 0 |
| **Halmahera Sea** | 75295 | 0 | 0 | 0 | 0 | 0 | 0 | 0 | 326 | 0 | 82 | 163 | 0 | | 27736 | 326 | 15173 | 28796 | 2692 | 0 | 0 | 0 | 0 | 0 | 0 | 0 | 0 |
| **Hudson Bay** | 832649 | 0 | 0 | 0 | 0 | 0 | 21 | 1058 | 5037 | 25397 | 116761 | 99322 | 9439 | | 563445 | 487 | 5566 | 3090 | 2222 | 212 | 169 | 190 | 85 | 42 | 0 | 42 | 63 |
| **Hudson Strait** | 200039 | 0 | 0 | 0 | 0 | 91 | 127 | 564 | 1855 | 9966 | 31591 | 23025 | 1164 | | 128910 | 18 | 1091 | 982 | 418 | 73 | 109 | 0 | 55 | 0 | 0 | 0 | 0 |
| **Indian Ocean** | 58230954 | 0 | 0 | 0 | 0 | 0 | 0 | 29028 | 313346 | 1005898 | 4536160 | 7374615 | 78585 | | 40570698 | 26990 | 2390307 | 1444056 | 341877 | 78287 | 19684 | 8251 | 4672 | 3430 | 1243 | 1690 | 2137 |
| **Inner Seas off the West Coast of Scotland** | 44260 | 0 | 0 | 0 | 0 | 26 | 0 | 103 | 981 | 2013 | 3690 | 3768 | 26 | | 28517 | 77 | 697 | 1368 | 1523 | 723 | 284 | 103 | 52 | 155 | 0 | 0 | 155 |
| **Ionian Sea** | 171778 | 0 | 0 | 0 | 0 | 0 | 0 | 51 | 101 | 51 | 101 | 6673 | 708 | | 152922 | 202 | 10717 | 253 | 0 | 0 | 0 | 0 | 0 | 0 | 0 | 0 | 0 |
| **Irish Sea and St Georges Channel** | 45922 | 0 | 0 | 0 | 0 | 0 | 0 | 29 | 407 | 465 | 553 | 2501 | 29 | | 35074 | 29 | 2734 | 2414 | 1047 | 523 | 29 | 87 | 0 | 0 | 0 | 0 | 0 |
| **Japan Sea** | 1066307 | 0 | 0 | 0 | 93 | 1021 | 6821 | 29884 | 68399 | 160835 | 116009 | 47007 | 1392 | | 592016 | 1114 | 27378 | 11230 | 1810 | 464 | 510 | 93 | 0 | 139 | 93 | 0 | 0 |
| **Java Sea** | 566693 | 0 | 0 | 0 | 0 | 0 | 0 | 0 | 0 | 82 | 327 | 572 | 245 | | 543666 | 163 | 17801 | 3021 | 490 | 245 | 0 | 82 | 0 | 0 | 0 | 0 | 0 |
| **Kara Sea** | 896938 | 0 | 0 | 0 | 0 | 0 | 1007 | 7474 | 19423 | 25591 | 29529 | 26747 | 1579 | | 662197 | 343 | 13425 | 18690 | 10495 | 9843 | 8778 | 8126 | 7874 | 6421 | 5391 | 4967 | 29036 |
| **Kattegat** | 35447 | 0 | 0 | 0 | 0 | 0 | 26 | 306 | 1097 | 3037 | 3496 | 2220 | 179 | | 25035 | 0 | 0 | 51 | 0 | 0 | 0 | 0 | 0 | 0 | 0 | 0 | 0 |
| **Labrador Sea** | 866470 | 0 | 0 | 51 | 0 | 25 | 178 | 839 | 3026 | 23779 | 71083 | 50813 | 610 | | 686233 | 203 | 6485 | 7909 | 5493 | 4247 | 3154 | 1246 | 381 | 178 | 178 | 76 | 280 |
| **Laccadive Sea** | 846351 | 0 | 0 | 0 | 0 | 0 | 323 | 1210 | 2903 | 13144 | 53703 | 58219 | 1451 | | 664760 | 403 | 31206 | 14272 | 1451 | 887 | 1210 | 403 | 323 | 161 | 0 | 81 | 242 |
| **Laptev Sea** | 513667 | 0 | 0 | 0 | 0 | 0 | 0 | 2802 | 30287 | 68270 | 58071 | 35469 | 1395 | | 304262 | 321 | 2015 | 2325 | 2170 | 2381 | 1572 | 1163 | 565 | 255 | 55 | 177 | 111 |
| **Ligurian Sea** | 16949 | 0 | 0 | 0 | 0 | 0 | 0 | 0 | 0 | 0 | 0 | 85 | 0 | | 8008 | 42 | 5763 | 3008 | 42 | 0 | 0 | 0 | 0 | 0 | 0 | 0 | 0 |
| **Makassar Strait** | 198117 | 0 | 0 | 0 | 0 | 0 | 0 | 0 | 0 | 0 | 247 | 823 | 0 | | 132984 | 329 | 40183 | 22068 | 1235 | 247 | 0 | 0 | 0 | 0 | 0 | 0 | 0 |
| **Malacca Strait** | 195534 | 0 | 0 | 0 | 0 | 0 | 0 | 0 | 0 | 0 | 0 | 164 | 0 | | 159087 | 1642 | 6567 | 13134 | 9194 | 3940 | 1395 | 328 | 0 | 82 | 0 | 0 | 0 |
| **Mediterranean Sea - Eastern Basin** | 1173640 | 0 | 0 | 0 | 0 | 0 | 0 | 0 | 56 | 224 | 895 | 60099 | 3466 | | 1073903 | 1062 | 32482 | 1174 | 0 | 112 | 56 | 0 | 56 | 0 | 0 | 0 | 56 |
| **Mediterranean Sea - Western Basin** | 476707 | 0 | 0 | 0 | 0 | 0 | 0 | 0 | 0 | 49 | 1264 | 13415 | 243 | | 429075 | 1166 | 23913 | 6464 | 875 | 146 | 0 | 0 | 49 | 0 | 49 | 0 | 0 |
| **Molukka Sea** | 221671 | 0 | 0 | 0 | 0 | 0 | 0 | 0 | 0 | 0 | 0 | 164 | 0 | | 144740 | 329 | 14548 | 40274 | 16931 | 4356 | 329 | 0 | 0 | 0 | 0 | 0 | 0 |
| **Mozambique Channel** | 1394283 | 0 | 0 | 0 | 0 | 0 | 0 | 0 | 0 | 73 | 435 | 10369 | 725 | | 995171 | 1450 | 303323 | 79257 | 3191 | 218 | 0 | 73 | 0 | 0 | 0 | 0 | 0 |
| **North Atlantic Ocean** | 34507128 | 0 | 0 | 0 | 257 | 1182 | 3546 | 19119 | 233180 | 1398306 | 2948382 | 4313436 | 76681 | | 21669867 | 54633 | 1872375 | 820523 | 286939 | 216373 | 210720 | 158040 | 98165 | 54222 | 28524 | 16344 | 26314 |
| **North Pacific Ocean** | 65154681 | 0 | 0 | 0 | 2918 | 19930 | 50228 | 111508 | 199485 | 334524 | 1402354 | 5346107 | 274051 | | 48503129 | 73076 | 5083542 | 3043060 | 478813 | 114736 | 51346 | 26511 | 14715 | 9313 | 5029 | 2856 | 7450 |
| **North Sea** | 524493 | 0 | 0 | 0 | 0 | 49 | 25 | 617 | 2098 | 5183 | 14685 | 28482 | 5109 | | 401385 | 913 | 17277 | 19967 | 13402 | 7849 | 3751 | 1802 | 790 | 420 | 247 | 25 | 420 |
| **Norwegian Sea** | 1437096 | 0 | 0 | 10 | 31 | 115 | 367 | 1134 | 5427 | 40988 | 155220 | 78974 | 577 | | 1059994 | 220 | 19985 | 35310 | 22935 | 7998 | 3558 | 1795 | 892 | 252 | 346 | 126 | 840 |
| **Persian Gulf** | 244657 | 0 | 0 | 0 | 0 | 0 | 0 | 0 | 0 | 66 | 198 | 198 | 0 | | 229382 | 1646 | 1843 | 2239 | 2173 | 1778 | 1975 | 1185 | 724 | 461 | 395 | 263 | 132 |
| **Philippine Sea** | 5641996 | 0 | 0 | 0 | 0 | 0 | 0 | 0 | 937 | 10096 | 44781 | 192537 | 8149 | | 3824068 | 16009 | 1424489 | 103913 | 12259 | 1803 | 649 | 865 | 793 | 288 | 144 | 216 | 0 |
| **Red Sea** | 449811 | 0 | 0 | 0 | 0 | 0 | 0 | 0 | 0 | 72 | 645 | 30250 | 1362 | | 352680 | 1505 | 13046 | 15555 | 12401 | 6953 | 5376 | 2724 | 2294 | 1649 | 932 | 1075 | 1290 |
| **Rio de La Plata** | 31797 | 0 | 0 | 0 | 0 | 0 | 0 | 0 | 0 | 382 | 255 | 382 | 0 | | 30586 | 64 | 64 | 64 | 0 | 0 | 0 | 0 | 0 | 0 | 0 | 0 | 0 |
| **Savu Sea** | 106234 | 0 | 0 | 0 | 0 | 0 | 0 | 0 | 0 | 0 | 80 | 398 | 0 | | 96757 | 80 | 5097 | 3663 | 80 | 80 | 0 | 0 | 0 | 0 | 0 | 0 | 0 |
| **Sea of Azov** | 39547 | 0 | 0 | 0 | 0 | 0 | 158 | 594 | 277 | 554 | 198 | 119 | 0 | | 7403 | 673 | 3167 | 1821 | 2217 | 2613 | 1940 | 2217 | 2098 | 1386 | 1425 | 1544 | 9145 |
| **Sea of Marmara** | 11675 | 0 | 0 | 0 | 0 | 0 | 0 | 0 | 96 | 144 | 144 | 0 | 48 | | 6316 | 0 | 48 | 0 | 335 | 526 | 813 | 670 | 1053 | 431 | 431 | 48 | 574 |
| **Sea of Okhotsk** | 1613208 | 0 | 0 | 0 | 0 | 901 | 7431 | 18634 | 51117 | 81658 | 80279 | 34369 | 1070 | | 1172434 | 394 | 22913 | 36902 | 28965 | 20154 | 13990 | 11287 | 9007 | 5742 | 3856 | 3490 | 8613 |
| **Seto Naikai or Inland Sea** | 18131 | 0 | 0 | 0 | 0 | 0 | 0 | 170 | 227 | 283 | 283 | 283 | 113 | | 14108 | 170 | 793 | 1077 | 567 | 0 | 0 | 0 | 57 | 0 | 0 | 0 | 0 |
| **Singapore Strait** | 2684 | 0 | 0 | 0 | 0 | 0 | 0 | 0 | 0 | 0 | 0 | 0 | 0 | | 1944 | 93 | 93 | 370 | 93 | 93 | 0 | 0 | 0 | 0 | 0 | 0 | 0 |
| **Skagerrak** | 32040 | 0 | 0 | 0 | 0 | 0 | 0 | 47 | 140 | 140 | 1233 | 558 | 0 | | 26828 | 47 | 1001 | 861 | 512 | 279 | 93 | 116 | 23 | 70 | 0 | 47 | 47 |
| **Solomon Sea** | 744334 | 0 | 0 | 0 | 0 | 0 | 0 | 0 | 0 | 80 | 2085 | 23982 | 401 | | 532103 | 1364 | 123842 | 57830 | 2486 | 160 | 0 | 0 | 0 | 0 | 0 | 0 | 0 |
| **South Atlantic Ocean** | 40501812 | 0 | 0 | 49 | 148 | 1680 | 9686 | 83615 | 494131 | 1119319 | 2343504 | 5491434 | 84505 | | 27449660 | 19026 | 1503347 | 967309 | 380569 | 183638 | 107435 | 74621 | 43241 | 30787 | 24165 | 17939 | 72002 |
| **South China Sea** | 3362904 | 0 | 0 | 0 | 0 | 0 | 0 | 155 | 155 | 311 | 3572 | 99252 | 4815 | | 2779354 | 9086 | 346371 | 106319 | 9086 | 1864 | 699 | 233 | 78 | 155 | 155 | 0 | 1243 |
| **South Pacific Ocean** | 76681173 | 0 | 0 | 211 | 316 | 896 | 2741 | 9119 | 37002 | 896742 | 6668192 | 11969528 | 228020 | | 52057530 | 54765 | 3126975 | 1088973 | 365222 | 109477 | 34894 | 11280 | 5745 | 4375 | 3268 | 1634 | 4269 |
| **Southern Ocean** | 21695157 | 964 | 0 | 44 | 4292 | 18263 | 64883 | 443825 | 1171228 | 1770857 | 1893134 | 669833 | 3175 | | 14009561 | 1007 | 120504 | 228241 | 235774 | 203803 | 179321 | 146037 | 116234 | 91007 | 74978 | 52642 | 195548 |
| **Strait of Gibraltar** | 1664 | 0 | 0 | 0 | 0 | 0 | 0 | 0 | 0 | 0 | 0 | 0 | 0 | | 1331 | 0 | 222 | 111 | 0 | 0 | 0 | 0 | 0 | 0 | 0 | 0 | 0 |
| **Sulu Sea** | 337029 | 0 | 0 | 0 | 0 | 0 | 0 | 0 | 0 | 80 | 241 | 1447 | 80 | | 293946 | 482 | 29338 | 10289 | 965 | 80 | 80 | 0 | 0 | 0 | 0 | 0 | 0 |
| **Tasman Sea** | 3344624 | 0 | 0 | 0 | 95 | 0 | 0 | 286 | 620 | 4864 | 46350 | 85786 | 1860 | | 2539738 | 2861 | 348296 | 274860 | 36146 | 2480 | 191 | 48 | 0 | 48 | 95 | 0 | 0 |
| **The Coastal Waters of Southeast Alaska and British Columbia** | 124532 | 0 | 0 | 0 | 0 | 164 | 164 | 589 | 1605 | 2030 | 1899 | 1670 | 0 | | 101544 | 0 | 1768 | 4224 | 2816 | 2194 | 950 | 851 | 524 | 458 | 393 | 229 | 458 |
| **The Northwestern Passages** | 1062173 | 0 | 0 | 0 | 34 | 34 | 151 | 639 | 5246 | 16412 | 37515 | 69531 | 15605 | | 752923 | 7432 | 75064 | 53910 | 15218 | 5314 | 2220 | 1446 | 1043 | 790 | 757 | 286 | 605 |
| **Timor Sea** | 434186 | 0 | 0 | 0 | 0 | 0 | 0 | 0 | 0 | 0 | 159 | 4763 | 635 | | 325679 | 1270 | 48340 | 52547 | 794 | 0 | 0 | 0 | 0 | 0 | 0 | 0 | 0 |
| **Tyrrhenian Sea** | 217465 | 0 | 0 | 0 | 0 | 0 | 0 | 0 | 0 | 0 | 0 | 285 | 48 | | 171293 | 998 | 40614 | 4038 | 143 | 48 | 0 | 0 | 0 | 0 | 0 | 0 | 0 |
| **White Sea** | 90531 | 0 | 0 | 0 | 0 | 0 | 0 | 297 | 769 | 5410 | 12318 | 6220 | 135 | | 57138 | 13 | 1295 | 2307 | 1606 | 702 | 486 | 486 | 297 | 283 | 162 | 121 | 486 |
| **Yellow Sea** | 408198 | 53 | 0 | 0 | 0 | 53 | 53 | 53 | 106 | 3243 | 14089 | 11590 | 2552 | | 331800 | 3615 | 17651 | 12494 | 6380 | 2658 | 904 | 585 | 159 | 159 | 0 | 0 | 0 |

**Table S9| Changes in the depth of the photic zone (*Z_photic_*) across the global ocean and International Hydrographic Office (IHO) regions between 2003 and 2022 calculated using the 1% of surface irradiance definition of Photic Zone Depth.** *Z_photic_* was calculated using Beers Law (see methods). The annual average of monthly maximum zenith surface solar irradiance was modelled at 9km resolution. The depth of the photic zone was defined as 1% of surface irradiance, a relative quantity. *K_d_*(490) was taken as the modelled values for 2003 and 2022 given by quantile regression on the median, with serial autocorrelation removed using ARIMA models. Note that because photic zone depth is quantified using a relative irradiance value, photic zone depths are identical whether illumination is from the sun or moon.

| IHO region | Total Area (km^2^) | Area where *Z_photic_* is decreasing (km^2^) | | | | | | | | | | | | Area no change in *Z_photic_* (km^2^) | Area where *Z_photic_* is increasing (km^2^) | | | | | | | | | | | |
| --- | --- | --- | --- | --- | --- | --- | --- | --- | --- | --- | --- | --- | --- | --- | --- | --- | --- | --- | --- | --- | --- | --- | --- | --- | --- | --- |
|  |  | >100m | 90-100m | 80-90m | 70-80m | 60-70m | 50-60m | 40-50m | 30-40m | 20-30m | 10-20m | 1-10m | 0-1m |  | 0-1m | 1-10m | 10-20m | 20-30m | 30-40m | 40-50m | 50-60m | 60-70m | 70-80m | 80-90m | 90-100m | >100m |
| **Global Ocean** | **359351781** | **40489** | **46372** | **108303** | **204712** | **374651** | **641019** | **1255798** | **2971390** | **8565058** | **25762340** | **33138119** | **548120** | **250181325** | **235959** | **18588214** | **11855056** | **3163404** | **851741** | **356792** | **182737** | **106703** | **68462** | **45156** | **27527** | **32336** |
| Adriatic Sea | 139454 | 0 | 0 | 0 | 0 | 0 | 0 | 0 | 0 | 352 | 4844 | 13342 | 1629 | 118758 | 0 | 484 | 44 | 0 | 0 | 0 | 0 | 0 | 0 | 0 | 0 | 0 |
| Aegean Sea | 191305 | 0 | 0 | 0 | 0 | 0 | 0 | 0 | 0 | 253 | 5373 | 15004 | 304 | 139550 | 203 | 20377 | 10037 | 203 | 0 | 0 | 0 | 0 | 0 | 0 | 0 | 0 |
| Alboran Sea | 54711 | 0 | 0 | 0 | 0 | 0 | 0 | 0 | 0 | 0 | 641 | 2939 | 0 | 39751 | 160 | 10632 | 588 | 0 | 0 | 0 | 0 | 0 | 0 | 0 | 0 | 0 |
| Andaman or Burma Sea | 613099 | 0 | 0 | 0 | 0 | 0 | 0 | 0 | 0 | 80 | 31259 | 80336 | 1989 | 485754 | 1273 | 9306 | 2625 | 477 | 0 | 0 | 0 | 0 | 0 | 0 | 0 | 0 |
| Arabian Sea | 4241184 | 0 | 0 | 0 | 0 | 0 | 0 | 462 | 6080 | 59797 | 259966 | 229798 | 2847 | 3187468 | 6003 | 323380 | 153378 | 11621 | 385 | 0 | 0 | 0 | 0 | 0 | 0 | 0 |
| Arafura Sea | 1025736 | 0 | 0 | 0 | 0 | 0 | 0 | 0 | 0 | 0 | 159 | 3340 | 1352 | 854117 | 3340 | 84537 | 61554 | 16144 | 1113 | 80 | 0 | 0 | 0 | 0 | 0 | 0 |
| Baffin Bay | 529960 | 0 | 0 | 0 | 0 | 0 | 0 | 0 | 840 | 9785 | 62240 | 62188 | 579 | 377699 | 202 | 12005 | 3275 | 892 | 254 | 0 | 0 | 0 | 0 | 0 | 0 | 0 |
| Balearic (Iberian Sea) | 80078 | 0 | 0 | 0 | 0 | 0 | 0 | 0 | 0 | 0 | 0 | 332 | 0 | 62362 | 190 | 14296 | 2850 | 47 | 0 | 0 | 0 | 0 | 0 | 0 | 0 | 0 |
| Bali Sea | 39916 | 0 | 0 | 0 | 0 | 0 | 0 | 0 | 0 | 0 | 0 | 81 | 0 | 27852 | 81 | 11173 | 567 | 162 | 0 | 0 | 0 | 0 | 0 | 0 | 0 | 0 |
| Baltic Sea | 215863 | 0 | 0 | 0 | 0 | 0 | 0 | 0 | 0 | 0 | 0 | 55287 | 16998 | 141493 | 1539 | 546 | 0 | 0 | 0 | 0 | 0 | 0 | 0 | 0 | 0 | 0 |
| Banda Sea | 693842 | 0 | 0 | 0 | 0 | 0 | 0 | 0 | 0 | 81 | 569 | 3168 | 81 | 438626 | 325 | 90731 | 118023 | 37202 | 4711 | 325 | 0 | 0 | 0 | 0 | 0 | 0 |
| Barentsz Sea | 1408430 | 5545 | 3674 | 6279 | 9433 | 18024 | 26559 | 33788 | 37569 | 41248 | 55961 | 37077 | 1249 | 1018328 | 322 | 33081 | 43396 | 25151 | 8795 | 1696 | 927 | 232 | 51 | 45 | 0 | 0 |
| Bass Strait | 112699 | 0 | 0 | 0 | 0 | 0 | 0 | 0 | 0 | 147 | 832 | 2641 | 440 | 103699 | 636 | 4256 | 49 | 0 | 0 | 0 | 0 | 0 | 0 | 0 | 0 | 0 |
| Bay of Bengal | 2207565 | 0 | 0 | 0 | 0 | 0 | 0 | 0 | 233 | 85900 | 603322 | 365075 | 1556 | 1121134 | 856 | 18985 | 7081 | 2179 | 1089 | 156 | 0 | 0 | 0 | 0 | 0 | 0 |
| Bay of Biscay | 174437 | 0 | 0 | 0 | 0 | 0 | 0 | 0 | 0 | 404 | 19885 | 32737 | 162 | 114217 | 0 | 5133 | 1697 | 202 | 0 | 0 | 0 | 0 | 0 | 0 | 0 | 0 |
| Bay of Fundy | 16034 | 0 | 0 | 0 | 0 | 0 | 0 | 0 | 0 | 0 | 43 | 3172 | 391 | 10472 | 304 | 1651 | 0 | 0 | 0 | 0 | 0 | 0 | 0 | 0 | 0 | 0 |
| Beaufort Sea | 431132 | 0 | 0 | 0 | 0 | 0 | 126 | 775 | 2367 | 2954 | 9447 | 21701 | 2576 | 344537 | 1236 | 20612 | 16841 | 4692 | 1885 | 189 | 691 | 440 | 63 | 0 | 0 | 0 |
| Bering Sea | 2336912 | 0 | 0 | 0 | 0 | 288 | 1549 | 7036 | 17171 | 32350 | 97139 | 206382 | 7656 | 1665303 | 4403 | 122276 | 118492 | 44011 | 9847 | 1483 | 686 | 177 | 177 | 44 | 266 | 177 |
| Bismarck Sea | 345053 | 0 | 0 | 0 | 0 | 0 | 0 | 0 | 0 | 0 | 1062 | 9721 | 327 | 238368 | 327 | 50810 | 41825 | 2614 | 0 | 0 | 0 | 0 | 0 | 0 | 0 | 0 |
| Black Sea | 423026 | 0 | 0 | 0 | 0 | 0 | 0 | 0 | 0 | 0 | 1514 | 107703 | 1990 | 221245 | 2249 | 70591 | 16696 | 1038 | 0 | 0 | 0 | 0 | 0 | 0 | 0 | 0 |
| Bristol Channel | 5748 | 0 | 0 | 0 | 0 | 0 | 0 | 0 | 0 | 0 | 0 | 1051 | 35 | 4591 | 0 | 70 | 0 | 0 | 0 | 0 | 0 | 0 | 0 | 0 | 0 | 0 |
| Caribbean Sea | 2852792 | 0 | 0 | 0 | 0 | 0 | 76 | 0 | 76 | 4948 | 175916 | 433053 | 8906 | 1982575 | 3958 | 228363 | 13854 | 990 | 76 | 0 | 0 | 0 | 0 | 0 | 0 | 0 |
| Celebes Sea | 457342 | 0 | 0 | 0 | 0 | 0 | 0 | 0 | 0 | 0 | 0 | 246 | 246 | 307605 | 575 | 90516 | 55689 | 2464 | 0 | 0 | 0 | 0 | 0 | 0 | 0 | 0 |
| Celtic Sea | 215080 | 0 | 0 | 0 | 0 | 0 | 0 | 0 | 0 | 204 | 10175 | 43901 | 272 | 147799 | 102 | 10584 | 1974 | 68 | 0 | 0 | 0 | 0 | 0 | 0 | 0 | 0 |
| Ceram Sea | 161489 | 0 | 0 | 0 | 0 | 0 | 0 | 0 | 0 | 0 | 0 | 1320 | 0 | 94601 | 330 | 30021 | 31424 | 3794 | 0 | 0 | 0 | 0 | 0 | 0 | 0 | 0 |
| Chukchi Sea | 346259 | 0 | 0 | 0 | 0 | 0 | 0 | 0 | 131 | 5686 | 36534 | 54116 | 4627 | 237775 | 736 | 5716 | 917 | 20 | 0 | 0 | 0 | 0 | 0 | 0 | 0 | 0 |
| Coral Sea | 4125541 | 0 | 0 | 0 | 0 | 0 | 0 | 0 | 0 | 147 | 22229 | 106669 | 1027 | 3308429 | 2128 | 467979 | 204461 | 12178 | 293 | 0 | 0 | 0 | 0 | 0 | 0 | 0 |
| Davis Strait | 749737 | 0 | 0 | 0 | 0 | 30 | 0 | 59 | 148 | 5550 | 32812 | 77492 | 900 | 612717 | 546 | 14022 | 4000 | 1373 | 89 | 0 | 0 | 0 | 0 | 0 | 0 | 0 |
| East Siberian Sea | 633057 | 0 | 0 | 0 | 0 | 0 | 0 | 0 | 15 | 2110 | 11197 | 57778 | 5531 | 527387 | 6646 | 22077 | 316 | 0 | 0 | 0 | 0 | 0 | 0 | 0 | 0 | 0 |
| Eastern China Sea | 761356 | 0 | 0 | 0 | 0 | 0 | 0 | 64 | 1858 | 13137 | 38834 | 73758 | 1410 | 552449 | 2691 | 65235 | 11663 | 256 | 0 | 0 | 0 | 0 | 0 | 0 | 0 | 0 |
| English Channel | 81419 | 0 | 0 | 0 | 0 | 0 | 0 | 0 | 0 | 0 | 68 | 1523 | 102 | 63747 | 237 | 13711 | 2031 | 0 | 0 | 0 | 0 | 0 | 0 | 0 | 0 | 0 |
| Flores Sea | 102816 | 0 | 0 | 0 | 0 | 0 | 0 | 0 | 0 | 0 | 0 | 162 | 0 | 95224 | 162 | 6219 | 969 | 81 | 0 | 0 | 0 | 0 | 0 | 0 | 0 | 0 |
| Great Australian Bight | 1326209 | 0 | 0 | 0 | 0 | 0 | 0 | 0 | 522 | 31335 | 92961 | 98392 | 2089 | 1074274 | 261 | 19689 | 6685 | 0 | 0 | 0 | 0 | 0 | 0 | 0 | 0 | 0 |
| Greenland Sea | 1186456 | 2875 | 1401 | 1360 | 3145 | 6086 | 19731 | 51568 | 82979 | 95338 | 100449 | 58006 | 590 | 714301 | 336 | 22352 | 17978 | 6528 | 1057 | 164 | 41 | 41 | 25 | 25 | 41 | 41 |
| Gulf of Aden | 263754 | 0 | 0 | 0 | 0 | 0 | 0 | 0 | 0 | 0 | 622 | 2489 | 0 | 175317 | 311 | 15323 | 36090 | 23179 | 8400 | 1633 | 311 | 78 | 0 | 0 | 0 | 0 |
| Gulf of Alaska | 415503 | 0 | 0 | 0 | 0 | 0 | 0 | 0 | 138 | 1223 | 14122 | 42111 | 1131 | 320390 | 485 | 23559 | 9668 | 2354 | 323 | 0 | 0 | 0 | 0 | 0 | 0 | 0 |
| Gulf of Aqaba | 3555 | 0 | 0 | 0 | 0 | 0 | 0 | 0 | 0 | 0 | 62 | 561 | 0 | 2931 | 0 | 0 | 0 | 0 | 0 | 0 | 0 | 0 | 0 | 0 | 0 | 0 |
| Gulf of Boni | 33237 | 0 | 0 | 0 | 0 | 0 | 0 | 0 | 0 | 0 | 0 | 5865 | 0 | 27127 | 81 | 163 | 0 | 0 | 0 | 0 | 0 | 0 | 0 | 0 | 0 | 0 |
| Gulf of Bothnia | 113419 | 0 | 0 | 0 | 0 | 0 | 0 | 0 | 0 | 0 | 0 | 46467 | 13497 | 52781 | 638 | 35 | 0 | 0 | 0 | 0 | 0 | 0 | 0 | 0 | 0 | 0 |
| Gulf of California | 180703 | 0 | 0 | 0 | 0 | 0 | 0 | 0 | 0 | 0 | 65 | 5720 | 390 | 142872 | 845 | 26780 | 3965 | 65 | 0 | 0 | 0 | 0 | 0 | 0 | 0 | 0 |
| Gulf of Finland | 57077 | 187 | 0 | 47 | 47 | 0 | 140 | 47 | 0 | 0 | 0 | 257 | 3384 | 51710 | 1260 | 0 | 0 | 0 | 0 | 0 | 0 | 0 | 0 | 0 | 0 | 0 |
| Gulf of Guinea | 754844 | 0 | 0 | 247 | 82 | 82 | 577 | 1567 | 3628 | 7916 | 21440 | 39334 | 907 | 553805 | 247 | 45931 | 48982 | 18719 | 5195 | 2556 | 1484 | 990 | 330 | 165 | 165 | 495 |
| Gulf of Mexico | 1566759 | 0 | 0 | 0 | 0 | 0 | 0 | 739 | 1276 | 9807 | 53739 | 93573 | 6919 | 1262059 | 1747 | 102373 | 32848 | 1679 | 0 | 0 | 0 | 0 | 0 | 0 | 0 | 0 |
| Gulf of Oman | 111892 | 0 | 0 | 0 | 0 | 0 | 0 | 0 | 0 | 0 | 0 | 747 | 0 | 73735 | 204 | 18807 | 16567 | 1765 | 68 | 0 | 0 | 0 | 0 | 0 | 0 | 0 |
| Gulf of Riga | 18692 | 0 | 0 | 0 | 0 | 0 | 0 | 0 | 0 | 0 | 0 | 505 | 1837 | 15936 | 413 | 0 | 0 | 0 | 0 | 0 | 0 | 0 | 0 | 0 | 0 | 0 |
| Gulf of St Lawrence | 290874 | 0 | 0 | 0 | 0 | 0 | 0 | 0 | 0 | 0 | 4101 | 61703 | 4864 | 202385 | 1887 | 14301 | 1416 | 218 | 0 | 0 | 0 | 0 | 0 | 0 | 0 | 0 |
| Gulf of Suez | 10438 | 0 | 0 | 0 | 0 | 0 | 0 | 0 | 0 | 0 | 0 | 0 | 0 | 10438 | 0 | 0 | 0 | 0 | 0 | 0 | 0 | 0 | 0 | 0 | 0 | 0 |
| Gulf of Thailand | 297927 | 0 | 0 | 0 | 0 | 0 | 0 | 0 | 0 | 0 | 0 | 720 | 880 | 293287 | 480 | 2560 | 0 | 0 | 0 | 0 | 0 | 0 | 0 | 0 | 0 | 0 |
| Gulf of Tomini | 56739 | 0 | 0 | 0 | 0 | 0 | 0 | 0 | 0 | 0 | 0 | 164 | 0 | 40540 | 0 | 7401 | 7730 | 905 | 0 | 0 | 0 | 0 | 0 | 0 | 0 | 0 |
| Halmahera Sea | 75295 | 0 | 0 | 0 | 0 | 0 | 0 | 0 | 0 | 0 | 408 | 245 | 0 | 26512 | 0 | 24962 | 23168 | 0 | 0 | 0 | 0 | 0 | 0 | 0 | 0 | 0 |
| Hudson Bay | 832649 | 0 | 0 | 0 | 0 | 0 | 0 | 0 | 0 | 2032 | 79322 | 152358 | 4487 | 582450 | 1079 | 9905 | 1016 | 0 | 0 | 0 | 0 | 0 | 0 | 0 | 0 | 0 |
| Hudson Strait | 200039 | 0 | 0 | 0 | 0 | 0 | 0 | 0 | 36 | 782 | 13549 | 50596 | 727 | 131729 | 0 | 2128 | 491 | 0 | 0 | 0 | 0 | 0 | 0 | 0 | 0 | 0 |
| Indian Ocean | 58230954 | 0 | 0 | 398 | 9146 | 52440 | 135250 | 255439 | 463706 | 1364228 | 5256896 | 5753457 | 50849 | 40552853 | 12029 | 1912832 | 1735680 | 499743 | 127993 | 39218 | 8400 | 298 | 0 | 50 | 50 | 0 |
| Inner Seas off the West Coast of Scotland | 44260 | 0 | 0 | 0 | 0 | 0 | 0 | 0 | 0 | 0 | 568 | 9652 | 258 | 28724 | 258 | 3536 | 1239 | 26 | 0 | 0 | 0 | 0 | 0 | 0 | 0 | 0 |
| Ionian Sea | 171778 | 0 | 0 | 0 | 0 | 0 | 0 | 0 | 0 | 0 | 202 | 7128 | 455 | 152669 | 51 | 10566 | 708 | 0 | 0 | 0 | 0 | 0 | 0 | 0 | 0 | 0 |
| Irish Sea and St Georges Channel | 45922 | 0 | 0 | 0 | 0 | 0 | 0 | 0 | 0 | 0 | 0 | 3577 | 349 | 35219 | 698 | 6020 | 58 | 0 | 0 | 0 | 0 | 0 | 0 | 0 | 0 | 0 |
| Japan Sea | 1066307 | 0 | 0 | 0 | 0 | 0 | 232 | 2181 | 20835 | 122366 | 196565 | 88677 | 1578 | 588675 | 974 | 39721 | 4130 | 371 | 0 | 0 | 0 | 0 | 0 | 0 | 0 | 0 |
| Java Sea | 566693 | 0 | 0 | 0 | 0 | 0 | 0 | 0 | 0 | 0 | 0 | 4246 | 1062 | 531499 | 1470 | 26783 | 1551 | 82 | 0 | 0 | 0 | 0 | 0 | 0 | 0 | 0 |
| Kara Sea | 896938 | 0 | 0 | 0 | 0 | 57 | 114 | 172 | 1362 | 5196 | 20636 | 62640 | 13769 | 671273 | 3777 | 36064 | 30719 | 18415 | 17351 | 8710 | 4601 | 1534 | 401 | 137 | 11 | 0 |
| Kattegat | 35447 | 0 | 0 | 0 | 0 | 0 | 0 | 0 | 0 | 0 | 0 | 6150 | 3777 | 25469 | 26 | 26 | 0 | 0 | 0 | 0 | 0 | 0 | 0 | 0 | 0 | 0 |
| Labrador Sea | 866470 | 0 | 0 | 0 | 0 | 0 | 0 | 0 | 51 | 1475 | 41988 | 103737 | 2798 | 686843 | 254 | 14674 | 10783 | 3611 | 254 | 0 | 0 | 0 | 0 | 0 | 0 | 0 |
| Laccadive Sea | 846351 | 0 | 0 | 0 | 0 | 81 | 161 | 1129 | 3306 | 14756 | 58703 | 53300 | 1210 | 660728 | 484 | 31367 | 19030 | 1693 | 403 | 0 | 0 | 0 | 0 | 0 | 0 | 0 |
| Laptev Sea | 513667 | 0 | 0 | 0 | 0 | 55 | 221 | 1085 | 3156 | 10620 | 36964 | 126695 | 9568 | 312966 | 1152 | 10897 | 288 | 0 | 0 | 0 | 0 | 0 | 0 | 0 | 0 | 0 |
| Ligurian Sea | 16949 | 0 | 0 | 0 | 0 | 0 | 0 | 0 | 0 | 0 | 0 | 85 | 0 | 7966 | 42 | 6271 | 2585 | 0 | 0 | 0 | 0 | 0 | 0 | 0 | 0 | 0 |
| Makassar Strait | 198117 | 0 | 0 | 0 | 0 | 0 | 0 | 0 | 0 | 0 | 0 | 1153 | 0 | 130843 | 494 | 48088 | 17539 | 0 | 0 | 0 | 0 | 0 | 0 | 0 | 0 | 0 |
| Malacca Strait | 195534 | 0 | 0 | 0 | 0 | 0 | 0 | 0 | 0 | 0 | 0 | 164 | 82 | 148662 | 821 | 25365 | 19783 | 657 | 0 | 0 | 0 | 0 | 0 | 0 | 0 | 0 |
| Mediterranean Sea - Eastern Basin | 1173640 | 0 | 0 | 0 | 0 | 0 | 0 | 56 | 112 | 559 | 11125 | 52384 | 1454 | 1071052 | 224 | 30022 | 6597 | 56 | 0 | 0 | 0 | 0 | 0 | 0 | 0 | 0 |
| Mediterranean Sea - Western Basin | 476707 | 0 | 0 | 0 | 0 | 0 | 0 | 0 | 0 | 49 | 1507 | 13512 | 194 | 427277 | 389 | 27315 | 6124 | 340 | 0 | 0 | 0 | 0 | 0 | 0 | 0 | 0 |
| Molukka Sea | 221671 | 0 | 0 | 0 | 0 | 0 | 0 | 0 | 0 | 0 | 0 | 164 | 0 | 143589 | 247 | 14877 | 49973 | 12740 | 82 | 0 | 0 | 0 | 0 | 0 | 0 | 0 |
| Mozambique Channel | 1394283 | 0 | 0 | 0 | 0 | 0 | 0 | 0 | 0 | 0 | 145 | 11530 | 508 | 992488 | 508 | 223268 | 160182 | 5511 | 145 | 0 | 0 | 0 | 0 | 0 | 0 | 0 |
| North Atlantic Ocean | 34507128 | 0 | 0 | 51 | 51 | 257 | 1799 | 9097 | 63267 | 924906 | 3750351 | 4222672 | 44097 | 21634919 | 21278 | 1885069 | 1282308 | 494781 | 126381 | 33458 | 8686 | 2056 | 925 | 154 | 206 | 360 |
| North Pacific Ocean | 65154681 | 0 | 0 | 0 | 0 | 0 | 683 | 9065 | 69723 | 311179 | 1682862 | 5596503 | 81830 | 48475128 | 47621 | 4697548 | 3262412 | 825009 | 86052 | 8816 | 248 | 0 | 0 | 0 | 0 | 0 |
| North Sea | 524493 | 0 | 0 | 0 | 0 | 0 | 0 | 0 | 0 | 0 | 6812 | 38428 | 2912 | 410221 | 2616 | 51410 | 11427 | 642 | 25 | 0 | 0 | 0 | 0 | 0 | 0 | 0 |
| Norwegian Sea | 1437096 | 0 | 0 | 0 | 0 | 10 | 10 | 367 | 4965 | 31500 | 136347 | 108312 | 672 | 1061138 | 336 | 46730 | 40358 | 5269 | 462 | 504 | 115 | 0 | 0 | 0 | 0 | 0 |
| Persian Gulf | 244657 | 0 | 0 | 0 | 0 | 0 | 0 | 0 | 0 | 0 | 0 | 922 | 263 | 211606 | 1975 | 24031 | 5530 | 329 | 0 | 0 | 0 | 0 | 0 | 0 | 0 | 0 |
| Philippine Sea | 5641996 | 0 | 0 | 0 | 0 | 0 | 216 | 793 | 3966 | 19037 | 75429 | 153886 | 4110 | 3819308 | 2019 | 1028236 | 510044 | 23797 | 1010 | 72 | 72 | 0 | 0 | 0 | 0 | 0 |
| Red Sea | 449811 | 0 | 0 | 0 | 0 | 0 | 0 | 0 | 0 | 0 | 3369 | 29032 | 358 | 345441 | 1147 | 31612 | 32759 | 5376 | 717 | 0 | 0 | 0 | 0 | 0 | 0 | 0 |
| Rio de La Plata | 31797 | 0 | 0 | 0 | 0 | 0 | 0 | 0 | 0 | 0 | 0 | 701 | 510 | 30204 | 319 | 64 | 0 | 0 | 0 | 0 | 0 | 0 | 0 | 0 | 0 | 0 |
| Savu Sea | 106234 | 0 | 0 | 0 | 0 | 0 | 0 | 0 | 0 | 0 | 0 | 478 | 0 | 96678 | 239 | 7326 | 1513 | 0 | 0 | 0 | 0 | 0 | 0 | 0 | 0 | 0 |
| Sea of Azov | 39547 | 0 | 0 | 0 | 0 | 0 | 0 | 0 | 0 | 0 | 0 | 317 | 1583 | 6571 | 1900 | 29175 | 0 | 0 | 0 | 0 | 0 | 0 | 0 | 0 | 0 | 0 |
| Sea of Marmara | 11675 | 0 | 0 | 0 | 0 | 0 | 0 | 0 | 0 | 0 | 0 | 144 | 287 | 6268 | 48 | 3971 | 957 | 0 | 0 | 0 | 0 | 0 | 0 | 0 | 0 | 0 |
| Sea of Okhotsk | 1613208 | 0 | 0 | 0 | 0 | 0 | 0 | 0 | 1267 | 20182 | 99589 | 148539 | 4447 | 1174291 | 2871 | 96380 | 50217 | 11372 | 3322 | 732 | 0 | 0 | 0 | 0 | 0 | 0 |
| Seto Naikai or Inland Sea | 18131 | 0 | 0 | 0 | 0 | 0 | 0 | 0 | 0 | 0 | 0 | 1586 | 113 | 13768 | 0 | 2153 | 510 | 0 | 0 | 0 | 0 | 0 | 0 | 0 | 0 | 0 |
| Singapore Strait | 2684 | 0 | 0 | 0 | 0 | 0 | 0 | 0 | 0 | 0 | 0 | 0 | 0 | 1944 | 0 | 740 | 0 | 0 | 0 | 0 | 0 | 0 | 0 | 0 | 0 | 0 |
| Skagerrak | 32040 | 0 | 0 | 0 | 0 | 0 | 0 | 0 | 0 | 0 | 0 | 1373 | 745 | 27084 | 209 | 2629 | 0 | 0 | 0 | 0 | 0 | 0 | 0 | 0 | 0 | 0 |
| Solomon Sea | 744334 | 0 | 0 | 0 | 0 | 0 | 0 | 0 | 0 | 0 | 6898 | 19410 | 401 | 528894 | 1123 | 110688 | 71065 | 5855 | 0 | 0 | 0 | 0 | 0 | 0 | 0 | 0 |
| South Atlantic Ocean | 40501812 | 0 | 99 | 198 | 1384 | 7314 | 37558 | 169850 | 478121 | 1022611 | 3024541 | 4853854 | 50357 | 27428922 | 17346 | 1807371 | 1078157 | 296262 | 106990 | 54805 | 27575 | 12849 | 8549 | 5733 | 4497 | 6869 |
| South China Sea | 3362904 | 0 | 0 | 0 | 0 | 0 | 0 | 0 | 0 | 78 | 16930 | 110124 | 6135 | 2726079 | 7844 | 327266 | 155867 | 11727 | 699 | 78 | 0 | 78 | 0 | 0 | 0 | 0 |
| South Pacific Ocean | 76681173 | 0 | 0 | 0 | 0 | 0 | 4006 | 94402 | 749789 | 2909549 | 7930683 | 7997255 | 136517 | 52037923 | 32311 | 2871335 | 1418511 | 375606 | 100200 | 20240 | 2846 | 0 | 0 | 0 | 0 | 0 |
| Southern Ocean | 21695157 | 31883 | 41146 | 99723 | 181424 | 289927 | 411942 | 615351 | 950257 | 1381513 | 1470112 | 563256 | 3131 | 14008707 | 1029 | 197847 | 362825 | 313052 | 233803 | 181095 | 125803 | 87898 | 57942 | 38803 | 22292 | 24394 |
| Strait of Gibraltar | 1664 | 0 | 0 | 0 | 0 | 0 | 0 | 0 | 0 | 0 | 0 | 0 | 0 | 1220 | 0 | 388 | 55 | 0 | 0 | 0 | 0 | 0 | 0 | 0 | 0 | 0 |
| Sulu Sea | 337029 | 0 | 0 | 0 | 0 | 0 | 0 | 0 | 0 | 0 | 161 | 1608 | 80 | 288641 | 241 | 32232 | 13423 | 643 | 0 | 0 | 0 | 0 | 0 | 0 | 0 | 0 |
| Tasman Sea | 3344624 | 0 | 0 | 0 | 0 | 0 | 0 | 0 | 0 | 858 | 32569 | 107245 | 2194 | 2534397 | 3624 | 413530 | 231705 | 18311 | 191 | 0 | 0 | 0 | 0 | 0 | 0 | 0 |
| The Coastal Waters of Southeast Alaska and British Columbia | 124532 | 0 | 0 | 0 | 0 | 0 | 0 | 0 | 0 | 0 | 98 | 5698 | 2325 | 101544 | 2685 | 11494 | 655 | 33 | 0 | 0 | 0 | 0 | 0 | 0 | 0 | 0 |
| The Northwestern Passages | 1062173 | 0 | 0 | 0 | 0 | 0 | 67 | 706 | 2438 | 10829 | 33748 | 55406 | 1497 | 809170 | 1177 | 81134 | 55474 | 7500 | 2001 | 757 | 235 | 34 | 0 | 0 | 0 | 0 |
| Timor Sea | 434186 | 0 | 0 | 0 | 0 | 0 | 0 | 0 | 0 | 0 | 79 | 9366 | 794 | 285753 | 2699 | 72946 | 61596 | 953 | 0 | 0 | 0 | 0 | 0 | 0 | 0 | 0 |
| Tyrrhenian Sea | 217465 | 0 | 0 | 0 | 0 | 0 | 0 | 0 | 0 | 0 | 48 | 475 | 0 | 170675 | 380 | 39522 | 6128 | 238 | 0 | 0 | 0 | 0 | 0 | 0 | 0 | 0 |
| White Sea | 90531 | 0 | 0 | 0 | 0 | 0 | 0 | 0 | 0 | 0 | 202 | 18470 | 5734 | 58002 | 1889 | 5154 | 823 | 135 | 81 | 27 | 13 | 0 | 0 | 0 | 0 | 0 |
| Yellow Sea | 408198 | 0 | 53 | 0 | 0 | 0 | 0 | 0 | 0 | 0 | 3349 | 31899 | 2233 | 317818 | 5104 | 46891 | 851 | 0 | 0 | 0 | 0 | 0 | 0 | 0 | 0 | 0 |


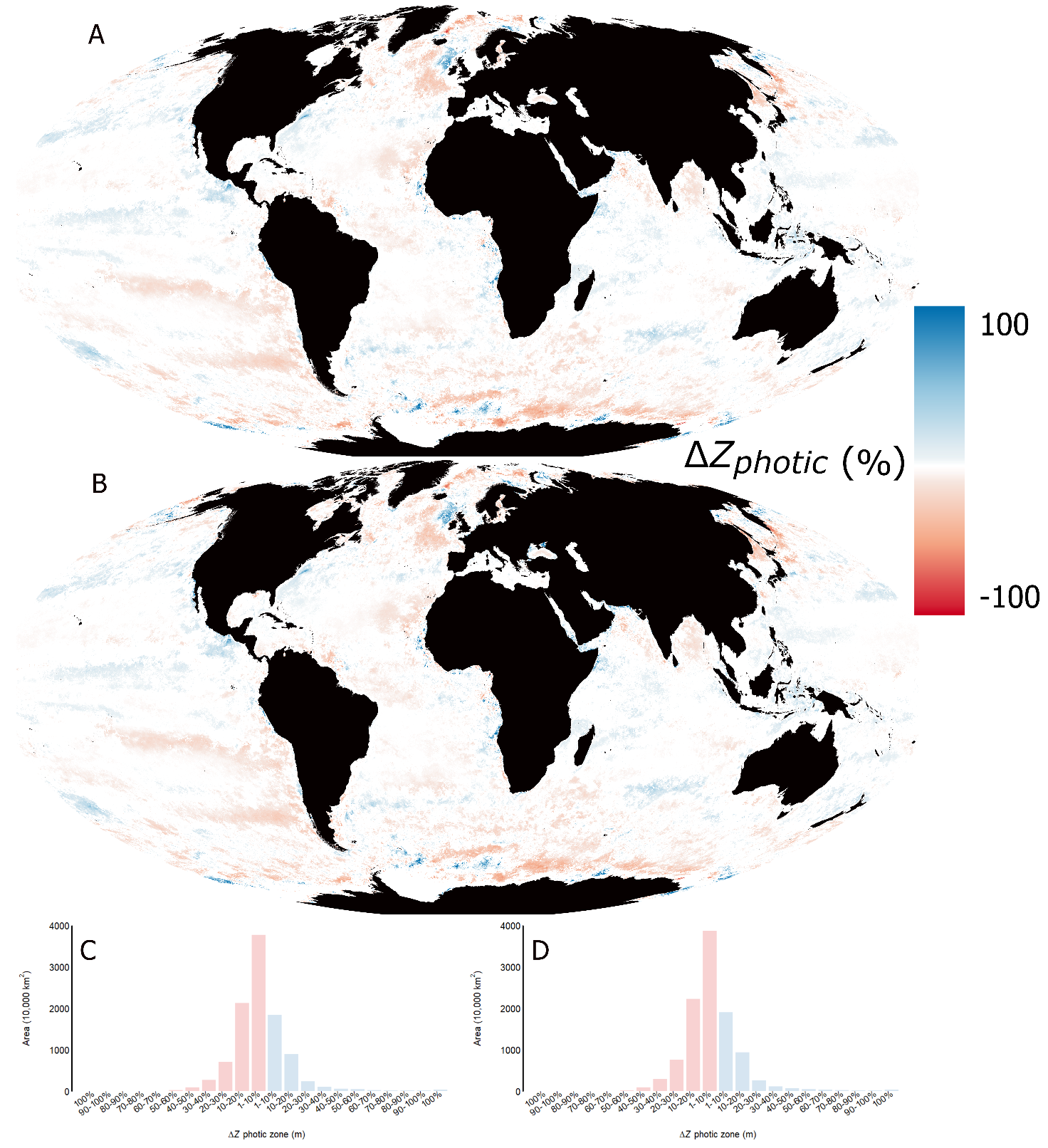


**Figure S1 | Loss of the global photic zone.** Percentage Change in photic zone depth (Δ*Z_photic_*) between 2003 and 2022 under sunlight (A,C) and full moon (B,D) irradiances. Changes in Δ*Z_photic_* expressed as a percentage difference to 2003 are given in Figure S1. The photic zone was defined as the depth at which the minimum irradiance of 490nm light occurs that elicits diel vertical migration in Calanus copepods. Surface irradiances at zenith were modelled at 9km resolution for the median day in each month of a typical year and used to quantify Δ*Z_photic_* with the annual average presented*_._* The area of the global ocean (in 10,000 km^2^) across which *Z_photic_* is reduced by 10 percentage point increment depths is presented in C and D. Red corresponds to reductions in *Z_photic_* while blue indicates increases. A breakdown of C and D by IHO region can be found in Tables S5 and S7.

1. [↑](#footnote-ref-1)
